# Supplementary material for: Satureja montana L. Essential Oils: Chemical Profiles/Phytochemical Screening, Antimicrobial Activity and O/W NanoEmulsion Formulations
Source: Pharmaceutics. 2019 Dec 19;12(1):7. doi: 10.3390/pharmaceutics12010007 (PMC7022231; doi:10.3390/pharmaceutics12010007)
Supplement: Supplementary file 1 [file pharmaceutics-12-00007-s001.zip › pharmaceutics-623925-SI-final/Supplementary Materials.docx]

Supplementary Materials: *Satureja montana* L. Essential Oils: Chemical Profiles/Phytochemical Screening, Antimicrobial Activity and O/W NanoEmulsion Formulations

Alessandro Maccelli ^1,^^†^, Luca Vitanza ^2,†^, Anna Imbriano ^1,†^, Caterina Fraschetti ^1^,
Antonello Filippi ^1^, Paola Goldoni ^2^, Linda Maurizi ^2^, Maria Grazia Ammendolia ^3^,
Maria Elisa Crestoni ^1^, Simonetta Fornarini ^1^, Luigi Menghini ^4^, Maria Carafa ^1^,
Carlotta Marianecci ^1^, Catia Longhi ^2,^* and Federica Rinaldi ^1,5^

^1^ Dipartimento di Chimica e Tecnologie del Farmaco, Sapienza Università di Roma-Piazzale Aldo Moro 5, 00185 Roma, Italy; [alessandro.maccelli@uniroma1.it](mailto:alessandro.maccelli@uniroma1.it) (A.M.); [anna.imbriano@uniroma1.it](mailto:anna.imbriano@uniroma1.it) (A.I.); [caterina.fraschetti@uniroma1.it](mailto:caterina.fraschetti@uniroma1.it) (C.F.); [antonello.filippi@uniroma1](mailto:antonello.filippi@uniroma1).it (A.F.); mariaelisa.crestoni@uniroma1.it (M.E.C.); [simonetta.fornarini@uniroma1.it](mailto:simonetta.fornarini@uniroma1.it) (S.F.); [carlotta.marianecci@uniroma1.it](mailto:carlotta.marianecci@uniroma1.it) (C.M.); [maria.carafa@uniroma1.it](mailto:maria.carafa@uniroma1.it) (M.C.); federica.rinaldi@uniroma1.it (F.R.)

^2^ Dipartimento di Sanità Pubblica e Malattie Infettive, Sapienza Università di Roma, Piazzale Aldo Moro 5, 00185 Roma, Italy; luca.vitanza@uniroma1.it (L.V.); paola.goldoni@uniroma1.it (P.G.); [lindamaurizi92@gmail.com](mailto:lindamaurizi92@gmail.com) (Linda.M.)

^3^ National Center of Innovative Technologies in Public Health, Italian National Institute of Health, Viale Regina Elena, 299, 00161 Rome, Italy; maria.ammendolia@iss.it

^4^ Dipartimento di Farmacia, Università G. d’Annunzio Chieti-Pescara, Via dei Vestini, 31, 66100 Chieti, Italy; luigi.menghini@unich.it (Luigi.M.)

^5^ Center for Life Nano Science@Sapienza, Fondazione Istituto Italiano di Tecnologia, Viale Regina Elena 291, 00161 Rome, Italy

***** Correspondence: [catia.longhi@uniroma1.it](mailto:catia.longhi@uniroma1.it); Tel.: +39-06-49914629

^†^ These equally contributed to the work.


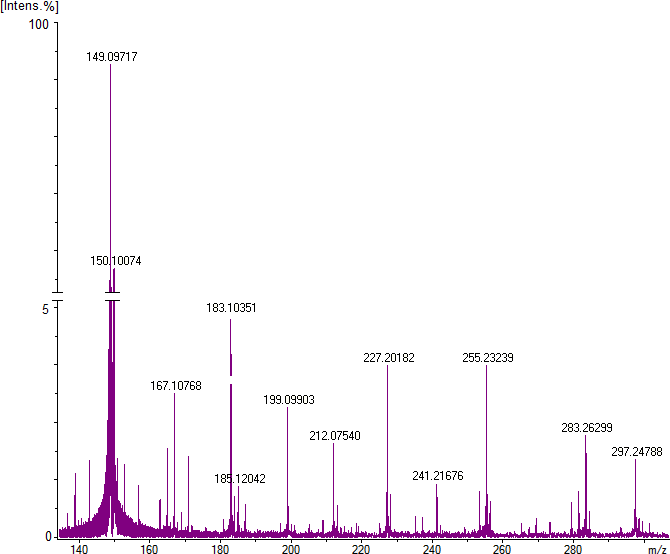


**Figure S1.** Portion of the ESI(-) FT-ICR mass spectrum of SEO 3 in the m/z 135-305 mass range. The prominent peak is assigned to a mixtures of carvacrol and thymol (m/z. 149.09717), while myristic, palmitic and stearic free fatty acids are recorded at m/z 227.20182, 255.23239 and 283.26299, respectively.


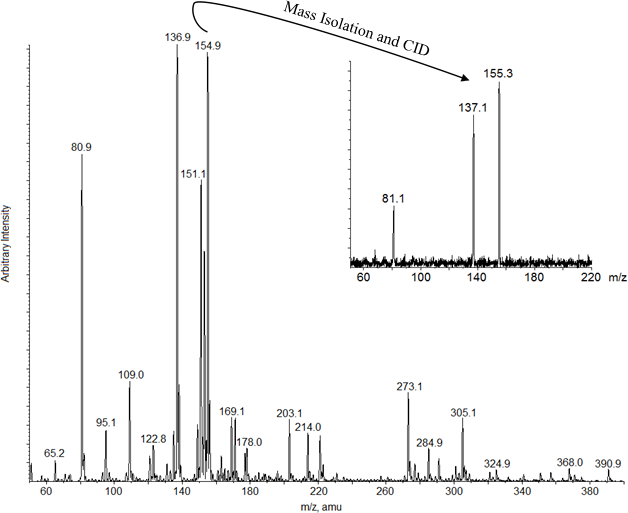


**Figure S2.** APCI(+)-MS spectrum of SEOT. This ionization source allowed to highlight less polar metabolites. The inset shows the CID spectrum of *m/z* 155 assigned to mixture of terpenenoids (see main text).

| 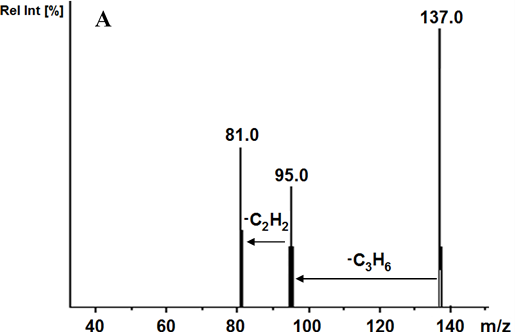 | 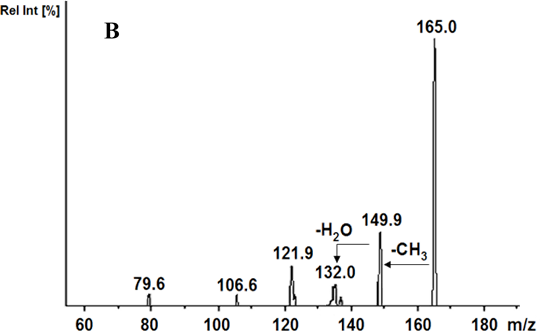 |
| --- | --- |
| (**A**) | (**B**) |

**Figure S3.** In panel A, CID spectra of protonated limonene (m/z 137) obtained by APCI(+) MS analysis; in panel B, CID of spectra of deprotonated limonene aldehyde (m/z 165) obtained by APCI(-) MS analysis.


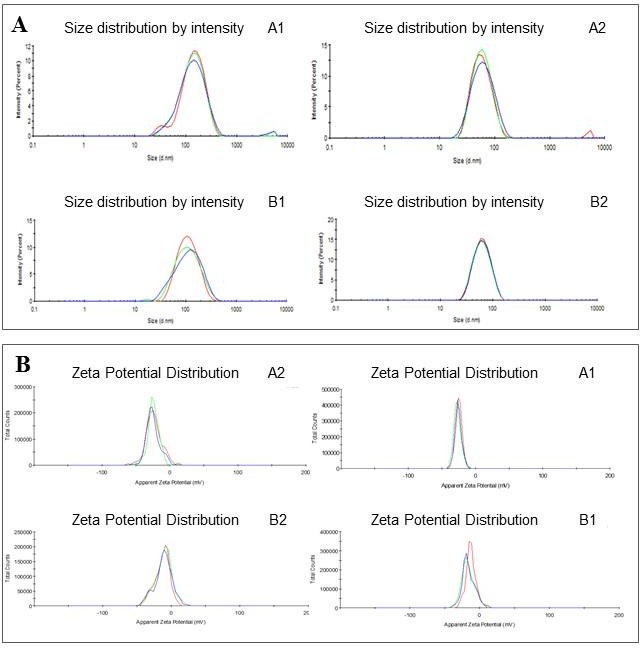


**Figure S4.** NEs DLS measurements.


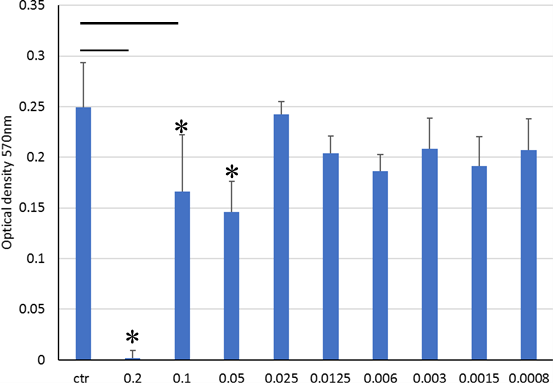


**Figure S5.** Cytotoxicity induced by SEO3 on T24 cell line after 24h exposition. Concentrations are expressed in mg/mL.

**Table S5.** Number of peaks recorded by ESI FT-ICR mass analysis of the sampled SEOs.

|  | **ESI(+)** | **ESI(-)** |
| --- | --- | --- |
| SEO1 | 2922 | 1232 |
| SEO2 | 702 | 1519 |
| SEO3 | 1536 | 1508 |
| SEOT | 1481 | 1534 |

**Table S6.** GC-MS analysis of *Satureja montana* L. essential oil (SEO 1).

| **Class** | **Compounds** | **Area %** | **KI^a^** | **KIL^b^** |
| --- | --- | --- | --- | --- |
|  | α-Pinene^c^ | 0.4 | 935 | 936 |
|  | Camphene^c^ | 0.1 | 949 | 953 |
|  | β-Pinene | 0.2 | 976 | 978 |
| **Terpene** | | | | |
|  | β-Myrcene | 0.1 | 985 | 991 |
|  | p-Cymene^c^ | 10.3 | 1024 | 1025 |
|  | Limonene^c^ | 1.3 | 1028 | 1030 |
|  | Eucalyptol | 6.2 | 1031 | 1038 |
|  | γ-Terpinene | 12.3 | 1059 | 1064 |
|  | Linaloxide (cis) | 0.4 | 1073 | 1069 |
|  | Linaloxide (trans) | 0.3 | 1089 | 1086 |
|  | Linalool | 16.1 | 1099 | 1100 |
| **Terpenoid** | Camphor | 0.2 | 1145 | 1141 |
|  | α-Terpineol | 1.5 | 1190 | 1195 |
|  | D-Carvone | 1.6 | 1244 | 1246 |
|  | Thymol^c^ | 16.5 | 1291 | 1293 |
|  | Carvacrol^c^ | 29.0 | 1300 | 1300 |
|  | Eugenol | 1.0 | 1358 | 1357 |
|  | E-Caryophyllene^c^ | 0.1 | 1423 | 1427 |
| **Sesquiterpene** | | | | |
|  | Caryophyllene oxide | 0.4 | 1591 | 1589 |
|  | Unidentified | 2.0 |  |  |
| Terpene |  | 12.4 |  |  |
| Terpenoid |  | 85.1 |  |  |
| Sesquiterpene |  | 0.5 |  |  |

aThe Kovats Index (KI) values have been experimentally measured by using *n*-alkanes mixtures (C_7_-C_40_). ^b^Literature values (see the main text). ^c^ Compounds identified by comparison with commercial standards analyzed in the same chromatographic conditions.

**Table S7.** GC-MS analysis of *Satureja montana* L. essential oil (SEO 2).

| **Class** | **Compounds** | **Area %** | **KI^a^** | **KIL^b^** |
| --- | --- | --- | --- | --- |
|  | α-Pinene^c^ | 0.4 | 934 | 936 |
|  | Camphene^c^ | 0.1 | 948 | 953 |
|  | β-Pinene | 0.2 | 976 | 978 |
| **Terpene** | | | | |
|  | β-Myrcene | 0.1 | 992 | 991 |
|  | p-Cymene^c^ | 9.4 | 1024 | 1025 |
|  | Limonene^c^ | 1.3 | 1028 | 1030 |
|  | Eucaliptol | 6.3 | 1031 | 1038 |
|  | γ-Terpinene | 14.7 | 1059 | 1064 |
|  | Linaloxide (cis) | 0.2 | 1074 | 1069 |
|  | Linaloxide (trans) | 0.2 | 1089 | 1086 |
|  | Linalool | 16.0 | 1099 | 1100 |
| **Terpenoid** | Camphor | 0.2 | 1145 | 1149 |
|  | α-Terpineol | 1.5 | 1190 | 1195 |
|  | D-Carvone | 1.5 | 1244 | 1246 |
|  | Thymol^c^ | 16.1 | 1291 | 1293 |
|  | Carvacrol^c^ | 28.7 | 1300 | 1300 |
|  | Eugenol | 0.8 | 1363 | 1357 |
|  | E-Caryophyllene^c^ | 0.2 | 1422 | 1427 |
| **Sesquiterpene** | | | | |
|  | Caryophyllene oxide | 0.2 | 1588 | 1589 |
|  | Unidentified | 1.9 |  |  |
| Terpene |  | 11.5 |  |  |
| Terpenoid |  | 86.2 |  |  |
| Sesquiterpene |  | 0.4 |  |  |

aThe Kovats Index (KI) values have been experimentally measured by using *n*-alkanes mixtures (C_7_-C_40_). ^b^Literature values (see the main text). ^c^Compounds identified by comparison with commercial standards analyzed in the same chromatographic conditions.

**Table S8.** GC-MS analysis of *Satureja montana* L. essential oil (SEO 3).

| **Class** | **Compounds** | **Area %** | **KI^a^** | **KIL^b^** |
| --- | --- | --- | --- | --- |
|  | α-Pinene^c^ | 0.4 | 936 | 936 |
|  | Camphene^c^ | 0.2 | 950 | 953 |
|  | β-Pinene | 0.2 | 977 | 978 |
| **Terpene** | | | | |
|  | β-Myrcene | 0.5 | 993 | 991 |
|  | p-Cymene^c^ | 12.3 | 1025 | 1025 |
|  | Limonene^c^ | 1.8 | 1029 | 1030 |
|  | Eucalyptol | 6.8 | 1032 | 1038 |
|  | Ocimene | 0.2 | 1050 | 1046 |
|  | γ-Terpinene | 15.0 | 1060 | 1064 |
|  | Linaloxide (cis) | 0.2 | 1076 | 1069 |
|  | Linaloxide (trans) | 0.1 | 1091 | 1086 |
|  | Linalool | 16.4 | 1101 | 1100 |
| **Terpenoid** | | | | |
|  | Camphor | 0.3 | 1146 | 1149 |
|  | Isoborneol | 0.5 | 1167 | 1160 |
|  | γ-Terpineol | 0.2 | 1199 | 1200 |
|  | D-Carvone | 1.6 | 1246 | 1246 |
|  | Thymol^c^ | 14.5 | 1292 | 1293 |
|  | Carvacrol^c^ | 23.9 | 1302 | 1300 |
|  | Eugenol | 0.4 | 1359 | 1357 |
|  | E-Caryophyllene^c^ | 0.4 | 1424 | 1427 |
| **Sesquiterpene** | | | | |
|  | α-Humulene | 0.1 | 1459 | 1454 |
|  | Unidentified | 4.0 |  |  |
| Terpene |  | 15.4 |  |  |
| Terpenoid |  | 80.1 |  |  |
| Sesquiterpene |  | 0.5 |  |  |

aThe Kovats Index (KI) values have been experimentally measured by using *n*-alkanes mixtures (C_7_-C_40_). ^b^Literature values (see the main text). ^c^Compounds identified by comparison with commercial standards analyzed in the same chromatographic conditions.
