# Supplementary material for: Satureja montana L. Essential Oils: Chemical Profiles/Phytochemical Screening, Antimicrobial Activity and O/W NanoEmulsion Formulations
Source: Pharmaceutics. 2019 Dec 19;12(1):7. doi: 10.3390/pharmaceutics12010007 (PMC7022231; doi:10.3390/pharmaceutics12010007)
Supplement: Supplementary file 1 [file pharmaceutics-12-00007-s001.zip › pharmaceutics-623925-SI-final/TableS1.docx]

Table S1. SEO 1 Untargeted ESI FT-ICR Annotations.

| No. | Compound (M)^a^ | Ion | Theor. m/z | Exp. m/z | Δppm | Formula |
| --- | --- | --- | --- | --- | --- | --- |
| 1 | Isopropylamine | [M+Na]+ | 82.06272 | 82.06291 | 2.3 | C3H9N |
| 2 | Urea | [M+Na]+ | 83.02158 | 83.02148 | -1.2 | CH4N2O |
| 3 | 2-Methylpropanal oxime | [M+H]+ | 88.07569 | 88.07565 | -0.5 | C4H9NO |
| 4 | D-Lactic acid | [M-H]- | 89.02442 | 89.02465 | 2.6 | C3H6O3 |
| 5 | 2-Methylpyridine | [M+H]+ | 94.06513 | 94.06504 | -0.9 | C6H7N |
| 6 | 3-Aminopropanal | [M+Na]+ | 96.04198 | 96.04193 | -0.6 | C3H7NO |
| 7 | (R)-Propane-1,2-diol | [M+Na]+ | 99.04165 | 99.04170 | 0.5 | C3H8O2 |
| 8 | Hexenal | [M-H]- | 99.08154 | 99.08160 | 0.6 | C6H12O |
| 9 | Ethanolamine | [M+K]+ | 100.01592 | 100.01598 | 0.6 | C2H7NO |
| 10 | Allyl isothiocyanate | [M+H]+ | 100.02155 | 100.02134 | -2.1 | C4H5NS |
| 11 | 2-Piperidinone | [M+H]+ | 100.07569 | 100.07566 | -0.3 | C5H9NO |
| 12 | 2-Methylpropanal O-methyloxime | [M+H]+ | 102.09134 | 102.09111 | -2.3 | C5H11NO |
| 13 | Hexylamine | [M+H]+ | 102.12773 | 102.12784 | 1.1 | C6H15N |
| 14 | 4-methylpirazole | [M+Na]+ | 105.04232 | 105.04226 | -0.6 | C4H6N2 |
| 15 | 2-Amino-2-methyl-1,3-propandiol | [M+H]+ | 106.08626 | 106.08651 | 2.4 | C4H11NO2 |
| 16 | 1,4-Lactone | [M+Na]+ | 109.02600 | 109.02612 | 1.1 | C4H6O2 |
| 17 | 3-Cresol | [M+H]+ | 109.06479 | 109.06485 | 0.5 | C7H8O |
| 18 | Creatinine | [M+H]+ | 114.06619 | 114.06605 | -1.2 | C4H7N3O |
| 19 | ε-Caprolactam | [M+H]+ | 114.09134 | 114.09138 | 0.3 | C6H11NO |
| 20 | 2,2-dimethyl-3-butenoic acid | [M+H]+ | 115.07536 | 115.07503 | -2.8 | C6H10O2 |
| 21 | 3-Amino-2-piperidone | [M+H]+ | 115.08659 | 115.08669 | 0.9 | C5H10N2O |
| 22 | Acetamidopropanal | [M+H]+ | 116.07060 | 116.07053 | -0.6 | C5H9NO2 |
| 23 | 5-Aminopentanamide | [M+H]+ | 117.10224 | 117.10212 | -1.0 | C5H12N2O |
| 24 | 1-Phenylethylamine | [M+H]+ | 122.09643 | 122.09648 | 0.4 | C8H11N |
| 25 | 2-Ethylacrylic acid | [M+Na]+ | 123.04165 | 123.04186 | 1.7 | C5H8O2 |
| 26 | D-Threitol | [M+H]+ | 123.06519 | 123.06498 | -1.7 | C4H10O4 |
| 27 | (S)-1-Phenylethanol | [M+H]+ | 123.08044 | 123.08064 | 1.6 | C8H10O |
| 28 | (S)-Methylmalonate semialdehyde | [M+Na]+ | 125.02091 | 125.02111 | 1.6 | C4H6O3 |
| 29 | Octanal | [M-H]- | 127.11284 | 127.11268 | -1.3 | C8H16O |
| 30 | (+/-)α-hydoxy butyric acid | [M+Na]+ | 127.03656 | 127.03641 | -1.2 | C4H8O3 |
| 31 | (4E)-2-Oxohexenoic acid | [M-H]- | 127.04007 | 127.03974 | -2.6 | C6H8O3 |
| 32 | Melamine | [M+H]+ | 127.07267 | 127.07271 | 0.3 | C3H6N6 |
| 33 | Diethylene glycol | [M+Na]+ | 129.05221 | 129.05235 | 1.0 | C4H10O3 |
| 34 | 4-Guanidinobutanal | [M+H]+ | 130.09749 | 130.09752 | 0.2 | C5H11N3O |
| 35 | Octylamine | [M+H]+ | 130.15903 | 130.15892 | -0.8 | C8H19N |
| 36 | Leucine | [M+H]+ | 132.10191 | 132.10172 | -1.4 | C6H13NO2 |
| 37 | Indoleamine | [M+H]+ | 133.07602 | 133.07595 | -0.6 | C8H8N2 |
| 38 | Hypoxanthine | [M-H]- | 135.03123 | 135.03157 | 2.5 | C5H4N4O |
| 39 | Cymene | [M+H]+ | 135.11683 | 135.11688 | 0.4 | C10H14 |
| 40 | Erythronic acid | [M+H]+ | 137.04445 | 137.04428 | -1.2 | C4H8O5 |
| 41 | Terpinene | [M+H]+ | 137.13248 | 137.13243 | -0.3 | C10H16 |
| 42 | 1-Indolizidinone | [M-H]- | 138.09244 | 138.09272 | 2.0 | C8H13NO |
| 43 | 2,4,6-Triaminotoluene | [M+H]+ | 138.10257 | 138.10226 | -2.3 | C7H11N3 |
| 44 | 4-Chlorobenzaldehyde | [M-H]- | 138.99562 | 138.99525 | -2.6 | C7H5ClO |
| 45 | 4-Nitroaniline | [M+H]+ | 139.05020 | 139.05036 | 1.1 | C6H6N2O2 |
| 46 | Nona-2,6-dienal | [M+H]+ | 139.11174 | 139.11166 | -0.6 | C9H14O |
| 47 | Tetrazolyl-glycine | [M-H]- | 142.03705 | 142.03707 | 0.2 | C3H5N5O2 |
| 48 | 2,4-Diaminotoluene | [M+Na]+ | 145.07362 | 145.07333 | -2.0 | C7H10N2 |
| 49 | Dehydrothiomorpholine-3-carboxylate | [M+H]+ | 146.02703 | 146.02736 | 2.3 | C5H7NO2S |
| 50 | Carvacrol | [M-H]- | 149.09609 | 149.09641 | 2.1 | C10H14O |
| 51 | Triethanolamine | [M+H]+ | 150.11247 | 150.11283 | 2.4 | C6H15NO3 |
| 52 | (+)-(S)-Carvone | [M+H]+ | 151.11174 | 151.11177 | 0.2 | C10H14O |
| 53 | 3-Methylene-indolenine | [M+Na]+ | 152.04707 | 152.04731 | 1.6 | C9H7N |
| 54 | (+)-Camphor | [M+H]+ | 153.12739 | 153.12747 | 0.5 | C10H16O |
| 55 | Bis(3-aminopropyl)amine | [M+Na]+ | 154.13147 | 154.13104 | -2.8 | C6H17N3 |
| 56 | (+)-Borneol | [M+H]+ | 155.14304 | 155.14342 | 2.4 | C10H18O |
| 57 | S,S-Dimethyl-β-propiothetin | [M+Na]+ | 157.02937 | 157.02953 | 1.0 | C5H10O2S |
| 58 | Purine | [M+K]+ | 159.00676 | 159.00630 | -2.9 | C5H4N4 |
| 59 | Tetrahydropteridine | [M+Na]+ | 159.06412 | 159.06421 | 0.6 | C6H8N4 |
| 60 | δ-Guanidinovaleric acid | [M+H]+ | 160.10805 | 160.10818 | 0.8 | C6H13N3O2 |
| 61 | Hydralazine | [M+H]+ | 161.08217 | 161.08178 | -2.4 | C8H8N4 |
| 62 | 2,3-Dihydroxytoluene | [M+K]+ | 163.01559 | 163.01569 | 0.6 | C7H8O2 |
| 63 | Methomyl | [M+H]+ | 163.05358 | 163.05349 | -0.5 | C5H10N2O2S |
| 64 | (1S,2S)-1,2-Dihydronaphthalene-1,2-diol | [M+H]+ | 163.07536 | 163.07583 | 2.9 | C10H10O2 |
| 65 | 1,1-dimethyl-3-phenylurea | [M-H]- | 163.08769 | 163.08796 | 1.7 | C9H12N2O |

| 66 | (-)-Anabasine | [M+H]+ | 163.12297 | 163.12273 | -1.5 | C10H14N2 |
| --- | --- | --- | --- | --- | --- | --- |
| 67 | Imidazole-4-acetate | [M+K]+ | 165.00609 | 165.00654 | 2.7 | C5H6N2O2 |
| 68 | 2-decene-4,6,8-triyn-1-al | [M+Na]+ | 165.03109 | 165.03132 | 1.4 | C10H6O |
| 69 | 2-heptenedial | [M+K]+ | 165.03124 | 165.03132 | 0.5 | C7H10O2 |
| 70 | 1-Hexenyl acetate | [M+Na]+ | 165.08860 | 165.08870 | 0.6 | C8H14O2 |
| 71 | 3-(2-Hydroxyphenyl)propanoate | [M+H]+ | 167.07027 | 167.06983 | -2.6 | C9H10O3 |
| 72 | (+)-Bornane-2,5-dione | [M+H]+ | 167.10666 | 167.10648 | -1.1 | C10H14O2 |
| 73 | (R)-3-Hydroxy-3-methyl-2-oxopentanoate | [M+Na]+ | 169.04713 | 169.04723 | 0.6 | C6H10O4 |
| 74 | (+)-Iridodial | [M+H]+ | 169.12231 | 169.12231 | 0.0 | C10H16O2 |
| 75 | Fagomine | [M+Na]+ | 170.07876 | 170.07926 | 2.9 | C6H13NO3 |
| 76 | Lupinine | [M+H]+ | 170.15394 | 170.15375 | -1.1 | C10H19NO |
| 77 | Ectocarpen | [M+Na]+ | 171.11442 | 171.11396 | -2.7 | C11H16 |
| 78 | (+)-Neomatatabiol | [M+H]+ | 171.13796 | 171.13821 | 1.5 | C10H18O2 |
| 79 | Decanamide | [M+H]+ | 172.16959 | 172.16995 | 2.1 | C10H21NO |
| 80 | 2-Phenylpropionate | [M+Na]+ | 173.05730 | 173.05780 | 2.9 | C9H10O2 |
| 81 | Pyrogallol 1,3-dimethyl ether | [M+Na]+ | 177.05221 | 177.05206 | -0.9 | C8H10O3 |
| 82 | (R)-6-Hydroxynicotine | [M+H]+ | 179.11789 | 179.11752 | -2.1 | C10H14N2O |
| 83 | 2-Hydroxy-3-(4-hydroxyphenyl)propenoate | [M+H]+ | 181.04954 | 181.04909 | -2.5 | C9H8O4 |
| 84 | 4-Guanidinobutanamide | [M+K]+ | 183.06427 | 183.06375 | -2.8 | C5H12N4O |
| 85 | 10-undecynoic acid | [M+H]+ | 183.13796 | 183.13780 | -0.9 | C11H18O2 |
| 86 | 2,4-decadiynoic acid | [M+Na]+ | 187.07295 | 187.07306 | 0.6 | C10H12O2 |
| 87 | 10-amino-decanoic acid | [M+H]+ | 188.16451 | 188.16447 | -0.2 | C10H21NO2 |
| 88 | 1-Phenyl-5-heptene-1,3-diyne | [M+Na]+ | 189.06747 | 189.06724 | -1.2 | C13H10 |
| 89 | N-(o)-Hydroxyarginine | [M+H]+ | 191.11387 | 191.11437 | 2.6 | C6H14N4O3 |
| 90 | Linalool oxide | [M+Na]+ | 193.11990 | 193.11975 | -0.8 | C10H18O2 |
| 91 | 2-Oxoadipic acid | [M+Cl]- | 195.00657 | 195.00678 | 1.1 | C6H8O5 |
| 92 | 2,3-Dihydroxy-p-cumate | [M-H]- | 195.06628 | 195.06592 | -1.9 | C10H12O4 |
| 93 | 4-Hexyloxyphenol | [M+H]+ | 195.13796 | 195.13755 | -2.1 | C12H18O2 |
| 94 | 1,3-Dimethyluric acid | [M+H]+ | 197.06692 | 197.06652 | -2.0 | C7H8N4O3 |
| 95 | 2-Propylglutaric acid | [M+Na]+ | 197.07843 | 197.07813 | -1.5 | C8H14O4 |
| 96 | Jasmone | [M+Cl]- | 199.08952 | 199.08936 | -0.8 | C11H16O |
| 97 | 1-Hexylglycerol | [M+Na]+ | 199.13047 | 199.13037 | -0.5 | C9H20O3 |
| 98 | 2-methyl-undecanoic acid | [M-H]- | 199.17035 | 199.17073 | 1.9 | C12H24O2 |
| 99 | 6-Methylthiohexanaldoxime | [M+K]+ | 200.05059 | 200.05032 | -1.4 | C7H15NOS |
| 100 | Ephedrine | [M+Cl]- | 200.08477 | 200.08449 | -1.4 | C10H15NO |
| 101 | Ecgonine methyl ester | [M+H]+ | 200.12812 | 200.12803 | -0.4 | C10H17NO3 |
| 102 | N-Methylnicotinium | [M+Na]+ | 200.12839 | 200.12803 | -1.8 | C11H17N2 |
| 103 | 1,1-Dimethylethyl benzoate | [M+Na]+ | 201.08860 | 201.08825 | -1.7 | C11H14O2 |
| 104 | 2,4-Diamino-6-nitrotoluene | [M+Cl]- | 202.03888 | 202.03888 | 0.0 | C7H9N3O2 |
| 105 | Fluoren-9-one | [M+Na]+ | 203.04674 | 203.04670 | -0.2 | C13H8O |
| 106 | Chlorphenesin | [M+H]+ | 203.04695 | 203.04670 | -1.2 | C9H11ClO3 |
| 107 | Hydroxy-L-homoarginine | [M-H]- | 203.11496 | 203.11466 | -1.5 | C7H16N4O3 |
| 108 | 5-Ureido-4-imidazole carboxylate | [M+Cl]- | 205.01339 | 205.01356 | 0.8 | C5H6N4O3 |
| 109 | (-)-5-oxo-1,2-campholide | [M+Na]+ | 205.08352 | 205.08377 | 1.2 | C10H14O3 |
| 110 | Tecostanine | [M+Na]+ | 206.15153 | 206.15176 | 1.1 | C11H21NO |
| 111 | Toluene-4-sulfonate | [M+Cl]- | 206.98882 | 206.98937 | 2.7 | C7H8O3S |
| 112 | 1,3-Dimethyl-8-isoquinolinol | [M+Cl]- | 208.05347 | 208.05361 | 0.7 | C11H11NO |
| 113 | Dihydrolipoamide | [M+H]+ | 208.08243 | 208.08242 | -0.1 | C8H17NOS2 |
| 114 | 3-Fluorocyclohexadiene-cis,cis-1,2-diol-1-carboxylate | [M+Cl]- | 209.00224 | 209.00227 | 0.2 | C7H7FO4 |
| 115 | Calligonine | [M+Na]+ | 209.10492 | 209.10545 | 2.5 | C12H14N2 |
| 116 | Hydroxydecanoic acid | [M+Na]+ | 211.13047 | 211.13054 | 0.4 | C10H20O3 |
| 117 | N-Heptanoylhomoserine lactone | [M-H]- | 212.12922 | 212.12937 | 0.7 | C11H19NO3 |
| 118 | Farfugin A | [M-H]- | 213.12849 | 213.12862 | 0.6 | C15H18O |
| 119 | (+)-(1R,2R)-1,2-Diphenylethane-1,2-diol | [M+H]+ | 215.10666 | 215.10620 | -2.1 | C14H14O2 |
| 120 | Diisopropyl phosphate | [M+Cl]- | 217.04020 | 217.04031 | 0.5 | C6H15O4P |
| 121 | 8-Hydroxyalanylclavam | [M+H]+ | 217.08190 | 217.08202 | 0.6 | C8H12N2O5 |
| 122 | γ-L-Glutamyl-D-alanine | [M-H]- | 217.08300 | 217.08245 | -2.5 | C8H14N2O5 |
| 123 | O-Propanoylcarnitine | [M+H]+ | 218.13868 | 218.13897 | 1.3 | C10H19NO4 |
| 124 | 5-Sulfosalicylate | [M+H]+ | 218.99579 | 218.99593 | 0.7 | C7H6O6S |
| 125 | L-β-aspartyl-L-serine | [M-H]- | 219.06226 | 219.06164 | -2.8 | C7H12N2O6 |
| 126 | 2-Succinylbenzoate | [M-H]- | 221.04555 | 221.04600 | 2.0 | C11H10O5 |
| 127 | 1,2-Dihydroxyfluorene | [M+Na]+ | 221.05730 | 221.05718 | -0.5 | C13H10O2 |
| 128 | Trimethyl-L-histidine | [M+Na]+ | 221.11347 | 221.11392 | 2.0 | C9H16N3O2 |
| 129 | Metoxadiazone | [M+H]+ | 223.07133 | 223.07192 | 2.6 | C10H10N2O4 |
| 130 | 2-tridecene-4,7-diynal | [M+Cl]- | 223.08952 | 223.08899 | -2.4 | C13H16O |
| 131 | 2E-Decenedioic acid | [M+Na]+ | 223.09408 | 223.09411 | 0.1 | C10H16O4 |
| 132 | 3,4-Diphenyltetrahydrofuran | [M-H]- | 223.11284 | 223.11333 | 2.2 | C16H16O |

| 133 | Capryloylglycine | [M+Na]+ | 224.12571 | 224.12624 | 2.3 | C10H19NO3 |
| --- | --- | --- | --- | --- | --- | --- |
| 134 | Anhalonidine | [M+H]+ | 224.12812 | 224.12817 | 0.2 | C12H17NO3 |
| 135 | 3-Hydroxy-L-kynurenine | [M+H]+ | 225.08698 | 225.08706 | 0.3 | C10H12N2O4 |
| 136 | (3R)-6-Hydroxy-3-isopropenyl-heptanoate | [M+K]+ | 225.08875 | 225.08836 | -1.7 | C10H18O3 |
| 137 | Diethyl adipate | [M+Na]+ | 225.10973 | 225.10970 | -0.1 | C10H18O4 |
| 138 | Propanoylagmatine | [M+K]+ | 225.11122 | 225.11068 | -2.4 | C8H18N4O |
| 139 | 1,8-Diazacyclotetradecane-2,9-dione | [M+H]+ | 227.17540 | 227.17507 | -1.5 | C12H22N2O2 |
| 140 | Myristic Acid | [M-H]- | 227.20165 | 227.20130 | -1.5 | C14H2802 |
| 141 | Actinamine | [M+Na]+ | 229.11588 | 229.11643 | 2.4 | C8H18N2O4 |
| 142 | 13-hydroxy-tridecanoic acid | [M-H]- | 229.18092 | 229.18070 | -1.0 | C13H26O3 |
| 143 | Xestoaminol C | [M+H]+ | 230.24784 | 230.24738 | -2.0 | C14H31NO |
| 144 | Cyano-nitroquinoxaline-dione | [M+H]+ | 233.03053 | 233.03024 | -1.2 | C9H4N4O4 |
| 145 | Goniothalenol | [M+H]+ | 233.08084 | 233.08045 | -1.7 | C13H12O4 |
| 146 | 10-tridecynoic acid | [M+Na]+ | 233.15120 | 233.15059 | -2.6 | C13H22O2 |
| 147 | 2,6-Diamino-7-hydroxy-azelaic acid | [M+H]+ | 235.12885 | 235.12936 | 2.2 | C9H18N2O5 |
| 148 | 10-hydroxy-11-dodecenoic acid | [M+Na]+ | 237.14612 | 237.14551 | -2.6 | C12H22O3 |
| 149 | Allenolic acid | [M+Na]+ | 239.06786 | 239.06758 | -1.2 | C13H12O3 |
| 150 | 3,4,5-Trimethoxycinnamic acid | [M+H]+ | 239.09140 | 239.09082 | -2.4 | C12H14O5 |
| 151 | 10E,12E-tetradecadiene-4,6-diynoic acid | [M+Na]+ | 239.10425 | 239.10470 | 1.9 | C14H16O2 |
| 152 | 2-hydroxy-10-undecenoic acid | [M+K]+ | 239.10440 | 239.10470 | 1.2 | C11H20O3 |
| 153 | Slaframine | [M-H]- | 239.14012 | 239.13981 | -1.3 | C12H20N2O3 |
| 154 | Cryptomeridiol | [M-H]- | 239.20165 | 239.20096 | -2.9 | C15H28O2 |
| 155 | Dinitro-(1-methylpropyl)phenol | [M+H]+ | 241.08190 | 241.08234 | 1.8 | C10H12N2O5 |
| 156 | 6-Hydroxytremetone | [M+Na]+ | 241.08352 | 241.08296 | -2.3 | C13H14O3 |
| 157 | Arginine-OEt | [M+K]+ | 241.10614 | 241.10572 | -1.7 | C8H18N4O2 |
| 158 | (+)-12-methyl myristic acid | [M-H]- | 241.21730 | 241.21732 | 0.1 | C15H30O2 |
| 159 | 1,2-Dimethoxy-4-[2-(2-propenyloxy)ethenyl]-benzene | [M+Na]+ | 243.09917 | 243.09934 | 0.7 | C13H16O3 |
| 160 | (+)-2-Sterpurene-6-ol | [M+Na]+ | 243.17194 | 243.17152 | -1.7 | C15H24O |
| 161 | N4-Phosphoagmatine | [M+Cl]- | 245.05758 | 245.05780 | 0.9 | C5H15N4O3P |
| 162 | Floxuridine | [M-H]- | 245.05792 | 245.05780 | -0.5 | C9H11FN2O5 |
| 163 | Dodecatetraenedioic acid | [M+Na]+ | 245.07843 | 245.07910 | 2.7 | C12H14O4 |
| 164 | Dihydroxy-4-methoxybenzophenone | [M+H]+ | 245.08084 | 245.08074 | -0.4 | C14H12O4 |
| 165 | 2-Methylbutyroylcarnitine | [M+H]+ | 246.16998 | 246.16989 | -0.4 | C12H23NO4 |
| 166 | 3,3'-Dimethylbenzidine | [M+Cl]- | 247.10075 | 247.10012 | -2.5 | C14H16N2 |
| 167 | Hinokiresinol | [M-H]- | 251.10775 | 251.10765 | -0.4 | C17H16O2 |
| 168 | N-Methyl-tetrahydroprotoberberine | [M+H]+ | 251.16685 | 251.16664 | -0.8 | C18H20N |
| 169 | 2,3-Dihydroxy-2'-carboxybiphenyl | [M+Na]+ | 253.04713 | 253.04768 | 2.2 | C13H10O4 |
| 170 | 3,4-Benzfluoranthene | [M+H]+ | 253.10118 | 253.10109 | -0.3 | C20H12 |
| 171 | Palmitoleic acid | [M-H]- | 253.21730 | 253.21729 | -0.1 | C16H30O2 |
| 172 | 4-Nitrophenyl phosphate | [M+Cl]- | 253.96268 | 253.96208 | -2.3 | C6H6NO6P |
| 173 | Succinyl proline | [M+K]+ | 254.04253 | 254.04278 | 1.0 | C9H13NO5 |
| 174 | Thiorphan | [M+H]+ | 254.08454 | 254.08474 | 0.8 | C12H15NO3S |
| 175 | Hydroxysepiapterin | [M+H]+ | 254.08838 | 254.08802 | -1.4 | C9H11N5O4 |
| 176 | Undecanedioic acid | [M+K]+ | 255.09932 | 255.09953 | 0.8 | C11H20O4 |
| 177 | Palmitic acid | [M-H]- | 255.23295 | 255.23304 | 0.3 | C16H32O2 |
| 178 | Streptamine phosphate | [M-H]- | 257.05441 | 257.05433 | -0.3 | C6H15N2O7P |
| 179 | (-)-Kanshone A | [M+Na]+ | 259.16685 | 259.16755 | 2.7 | C15H24O2 |
| 180 | 5-Acetylamino-6-formylamino-3-methyluracil | [M+Cl]- | 261.03961 | 261.04029 | 2.6 | C8H10N4O4 |
| 181 | Maclurin | [M-H]- | 261.04046 | 261.04029 | -0.7 | C13H10O6 |
| 182 | 2'-Hydroxyflavone | [M+Na]+ | 261.05221 | 261.05296 | 2.9 | C15H10O3 |
| 183 | (2S)-Flavan-4-ol | [M+Cl]- | 261.06878 | 261.06877 | 0.0 | C15H14O2 |
| 184 | Diazoxide | [M+Cl]- | 264.96108 | 264.96158 | 1.9 | C8H7ClN2O2S |
| 185 | Dehydrofalcarinol | [M+Na]+ | 265.15629 | 265.15588 | -1.5 | C17H22O |
| 186 | Myristoleic acid | [M+K]+ | 265.15644 | 265.15588 | -2.1 | C14H26O2 |
| 187 | 2-Hydroxy-4-methoxybenzophenone | [M+K]+ | 267.04180 | 267.04206 | 1.0 | C14H12O3 |
| 188 | (+)-Blennin D | [M+H]+ | 267.15909 | 267.15888 | -0.8 | C15H22O4 |
| 189 | Leucyl-leucine | [M+Na]+ | 267.16791 | 267.16864 | 2.7 | C12H24N2O3 |
| 190 | 12-heptadecynoic acid | [M+H]+ | 267.23186 | 267.23169 | -0.6 | C17H30O2 |
| 191 | omega-Cyclohexylundecanoic acid | [M-H]- | 267.23295 | 267.23342 | 1.7 | C17H32O2 |
| 192 | Agaritine | [M+H]+ | 268.12918 | 268.12864 | -2.0 | C12H17N3O4 |
| 193 | Ellipticine | [M+Na]+ | 269.10492 | 269.10514 | 0.8 | C17H14N2 |
| 194 | Camoensine | [M+K]+ | 269.10507 | 269.10514 | 0.3 | C14H18N2O |
| 195 | Methyl palmitate | [M-H]- | 269.24860 | 269.24887 | 1.0 | C17H34O2 |
| 196 | Deoxynupharidine | [M+K]+ | 272.14112 | 272.14127 | 0.5 | C15H23NO |
| 197 | Phospho-dehydro-gluconate | [M-H]- | 273.00171 | 273.00200 | 1.1 | C6H11O10P |
| 198 | (+)-Armillarin | [M+Na]+ | 273.14612 | 273.14586 | -0.9 | C15H22O3 |
| 199 | 4-[1-Ethyl-2-(4-fluorophenyl)butyl]phenol | [M+H]+ | 273.16492 | 273.16568 | 2.8 | C18H21FO |

| 200 | N(6)-(Octanoyl)lysine | [M+H]+ | 273.21727 | 273.21787 | 2.2 | C14H28N2O3 |
| --- | --- | --- | --- | --- | --- | --- |
| 201 | L-Tyrosine methyl ester 4-sulfate | [M-H]- | 274.03908 | 274.03899 | -0.3 | C10H13NO6S |
| 202 | Benzphetamine | [M+Cl]- | 274.13680 | 274.13732 | 1.9 | C17H21N |
| 203 | (5-L-Glutamyl)-L-glutamate | [M+H]+ | 277.10303 | 277.10347 | 1.6 | C10H16N2O7 |
| 204 | Palmitic amide | [M+Na]+ | 278.24544 | 278.24627 | 3.0 | C16H33NO |
| 205 | 3,3'-Dimethylbisphenol A | [M+Na]+ | 279.13555 | 279.13606 | 1.8 | C17H20O2 |
| 206 | 3-oxo-2-pentyl-cyclopentanebutanoic acid | [M+K]+ | 279.13570 | 279.13606 | 1.3 | C14H24O3 |
| 207 | (Acetyloxy)-(pyridinyl)-benzopyranone | [M-H]- | 280.06153 | 280.06148 | -0.2 | C16H11NO4 |
| 208 | 8-Oxocoformycin | [M-H]- | 281.08914 | 281.08878 | -1.3 | C11H14N4O5 |
| 209 | 1,9Z,16-heptadecatrien-4,6-diyn-3,8-diol | [M+Na]+ | 281.15120 | 281.15073 | -1.7 | C17H22O2 |
| 210 | 10-keto myristic acid | [M+K]+ | 281.15135 | 281.15073 | -2.2 | C14H26O3 |
| 211 | Oleic acid | [M-H]- | 281.24860 | 281.24859 | 0.0 | C18H34O2 |
| 212 | Cadiamine | [M+H]+ | 283.20162 | 283.20227 | 2.3 | C15H26N2O3 |
| 213 | Stearic acid | [M-H]- | 283.26425 | 283.26486 | 2.1 | C18H36O2 |
| 214 | 5-propylideneisolongifolane | [M+K]+ | 285.19791 | 285.19876 | 3.0 | C18H30 |
| 215 | 11-cis retro-γ-retinal | [M+H]+ | 285.22129 | 285.22195 | 2.3 | C20H28O |
| 216 | 3-oxo-2-pentyl-cyclopentanehexanoic acid | [M+Na]+ | 291.19307 | 291.19340 | 1.1 | C16H28O3 |
| 217 | L-γ-Glutamyl-L-hypoglycin | [M+Na]+ | 293.11079 | 293.11026 | -1.8 | C12H18N2O5 |
| 218 | Embelin | [M-H]- | 293.17583 | 293.17608 | 0.8 | C17H26O4 |
| 219 | Axerophthene | [M+Na]+ | 293.22397 | 293.22349 | -1.6 | C20H30 |
| 220 | 11Z-heptadecen-1-ol | [M+K]+ | 293.22413 | 293.22349 | -2.2 | C17H34O |
| 221 | 5-Fluorouridine | [M+Cl]- | 297.02952 | 297.02902 | -1.7 | C9H11FN2O6 |
| 222 | (S)-N-Methylcoclaurine | [M-H]- | 298.14487 | 298.14547 | 2.0 | C18H21NO3 |
| 223 | 1-(4-Hydroxyphenyl)-1-decene-3,5-dione | [M+K]+ | 299.10440 | 299.10483 | 1.4 | C16H20O3 |
| 224 | Allylestrenol | [M-H]- | 299.23804 | 299.23838 | 1.1 | C21H32O |
| 225 | 18-fluoro-9Z-octadecenoic acid | [M-H]- | 299.23918 | 299.23838 | -2.7 | C18H33FO2 |
| 226 | Dimepiperate | [M+K]+ | 302.09754 | 302.09720 | -1.1 | C15H21NOS |
| 227 | (R)-laballenic acid | [M+Na]+ | 303.22945 | 303.23004 | 1.9 | C18H32O2 |
| 228 | 2-(Formamido)-N1-(5'-phosphoribosyl)acetamidine | [M-H]- | 312.06022 | 312.05933 | -2.9 | C8H16N3O8P |
| 229 | Acetylcaranine | [M-H]- | 312.12413 | 312.12444 | 1.0 | C18H19NO4 |
| 230 | Wedelolactone | [M-H]- | 313.03538 | 313.03599 | 2.0 | C16H10O7 |
| 231 | 1-Dehydroprogesterone | [M+H]+ | 313.21621 | 313.21700 | 2.5 | C21H28O2 |
| 232 | Methyl-epoxy-trimethyltridecadienoate | [M+Cl]- | 315.17325 | 315.17389 | 2.0 | C17H28O3 |
| 233 | Lecanoric acid | [M-H]- | 317.06668 | 317.06644 | -0.7 | C16H14O7 |
| 234 | C17H17NO3 | [M+Cl]- | 318.09024 | 318.08996 | -0.9 | C17H17NO3 |
| 235 | Methyltrienolone | [M+Cl]- | 319.14703 | 319.14750 | 1.5 | C19H24O2 |
| 236 | Fluoro-dihydroxyandrosta-dienone | [M+H]+ | 321.18605 | 321.18647 | 1.3 | C19H25FO3 |
| 237 | L-Octanoylcarnitine | [M+Cl]- | 322.17906 | 322.17946 | 1.2 | C15H29NO4 |
| 238 | 1-Methylestradiol | [M+K]+ | 325.15644 | 325.15703 | 1.8 | C19H26O2 |
| 239 | Trichodermin | [M+Cl]- | 327.13686 | 327.13694 | 0.2 | C17H24O4 |
| 240 | 4-(3,5-Diphenylcyclohexyl)phenol | [M-H]- | 327.17544 | 327.17566 | 0.7 | C24H24O |
| 241 | (+)-Serradiol | [M+Na]+ | 327.22945 | 327.22985 | 1.2 | C20H32O2 |
| 242 | (Methyl-dioxolo-benzodiazepin-yl)benzenamine | [M+Cl]- | 328.08583 | 328.08559 | -0.7 | C17H15N3O2 |
| 243 | (5Z,9E,14Z)-icosa-5,9,14-trienoic acid | [M+Na]+ | 329.24510 | 329.24607 | 2.9 | C20H34O2 |
| 244 | Bucharaine | [M-H]- | 330.17108 | 330.17063 | -1.4 | C19H25NO4 |
| 245 | 7'-carboxy-gama-tocotrienol | [M-H]- | 330.18366 | 330.18328 | -1.1 | C20H27O4 |
| 246 | Hydroxy-methylenedioxycoumestan | [M+Cl]- | 331.00149 | 331.00181 | 1.0 | C16H8O6 |
| 247 | Sulochrin | [M-H]- | 331.08233 | 331.08247 | 0.4 | C17H16O7 |
| 248 | 5'-Methylthioadenosine | [M+Cl]- | 332.05896 | 332.05881 | -0.5 | C11H15N5O3S |
| 249 | p-Coumaryl alcohol 4-O-glucoside | [M+Na]+ | 335.11012 | 335.11074 | 1.8 | C15H20O7 |
| 250 | Nocodazole | [M+Cl]- | 336.02151 | 336.02088 | -1.9 | C14H11N3O3S |
| 251 | 2,3-Dehydro-gibberellin A9 | [M+Na]+ | 337.14103 | 337.14159 | 1.7 | C19H22O4 |
| 252 | Tetranor-PGD1 | [M+K]+ | 337.14118 | 337.14159 | 1.2 | C16H26O5 |
| 253 | Pergolide | [M+Na]+ | 337.17089 | 337.17148 | 1.7 | C19H26N2S |
| 254 | Dihydroxyoctadecenoic acid | [M+Na]+ | 337.23493 | 337.23527 | 1.0 | C18H34O4 |
| 255 | Arachidyl alcohol | [M+K]+ | 337.28673 | 337.28683 | 0.3 | C20H42O |
| 256 | Olopatadine | [M+H]+ | 338.17507 | 338.17606 | 2.9 | C21H23NO3 |
| 257 | Pentadecanoylglycine | [M+K]+ | 338.20920 | 338.20897 | -0.7 | C17H33NO3 |
| 258 | Hydroperoxy-eicosadienoic acid | [M-H]- | 339.25408 | 339.25416 | 0.2 | C20H36O4 |
| 259 | Isoprothiolane sulfoxide | [M+Cl]- | 341.02897 | 341.02821 | -2.2 | C12H18O5S2 |
| 260 | Phaseollidin hydrate | [M-H]- | 341.13945 | 341.14031 | 2.5 | C20H22O5 |
| 261 | (+)-18-Hydroxy-sacculatadienedial | [M+Na]+ | 341.20872 | 341.20853 | -0.5 | C20H30O3 |
| 262 | MG(0:0/14:0/0:0) | [M+K]+ | 341.20887 | 341.20853 | -1.0 | C17H34O4 |
| 263 | (+)-Beyerol | [M+Na]+ | 343.22437 | 343.22361 | -2.2 | C20H32O3 |
| 264 | 16,17-epoxy-DHA | [M+H]+ | 343.22677 | 343.22641 | -1.1 | C22H30O3 |
| 265 | 11,14-eicosadienoic acid | [M+Cl]- | 343.24093 | 343.24132 | 1.1 | C20H36O2 |
| 266 | Spiredine | [M+H]+ | 354.20637 | 354.20643 | 0.2 | C22H27NO3 |

| 267 | Dimercaptoandrostane-diol | [M-H]- | 355.17710 | 355.17744 | 1.0 | C19H32O2S2 |
| --- | --- | --- | --- | --- | --- | --- |
| 268 | Hydroxyprogesterone | [M+Na]+ | 355.18798 | 355.18888 | 2.5 | C20H28O4 |
| 269 | PA(12:0/0:0) | [M+H]+ | 355.18802 | 355.18888 | 2.4 | C15H31O7P |
| 270 | Dimethoxyestra-pentaene-carboxylic acid methyl ester | [M+H]+ | 355.19039 | 355.19035 | -0.1 | C22H26O4 |
| 271 | β-Citryl-L-glutamic acid | [M+Cl]- | 356.03900 | 356.03999 | 2.8 | C11H15NO10 |
| 272 | Thiocyanato-androstenetrione | [M-H]- | 356.13259 | 356.13264 | 0.1 | C20H23NO3S |
| 273 | Coumeroic acid | [M-H]- | 357.07282 | 357.07228 | -1.5 | C17H14N2O7 |
| 274 | Dihydroxy-19-serrulatanoic acid | [M+Na]+ | 357.20363 | 357.20454 | 2.5 | C20H30O4 |
| 275 | Galipine | [M+Cl]- | 358.12155 | 358.12257 | 2.9 | C20H21NO3 |
| 276 | Lobelanine | [M+Na]+ | 358.17775 | 358.17853 | 2.2 | C22H25NO2 |
| 277 | Fructose 1,6-bisphosphate | [M+Cl]- | 358.97054 | 358.97044 | -0.3 | C6H14O11P2 |
| 278 | Acetohexamide | [M+Cl]- | 359.08378 | 359.08367 | -0.3 | C15H20N2O4S |
| 279 | Tetrahydroxyandrostan-17-one | [M+Na]+ | 361.19854 | 361.19752 | -2.8 | C19H30O5 |
| 280 | Justicidin B | [M-H]- | 363.08741 | 363.08803 | 1.7 | C21H16O6 |
| 281 | Cardiopetalidine | [M+H]+ | 364.24824 | 364.24766 | -1.6 | C21H33NO4 |
| 282 | (2-Hydroxy-3-methylbut-3-enyl)-trihydroxychalcone | [M+Na]+ | 365.09956 | 365.10001 | 1.2 | C19H18O6 |
| 283 | Hinokitiol glucoside | [M+K]+ | 365.09971 | 365.10001 | 0.8 | C16H22O7 |
| 284 | Carnosol | [M+Cl]- | 365.15251 | 365.15229 | -0.6 | C20H26O4 |
| 285 | Eicosanedioic acid | [M+Na]+ | 365.26623 | 365.26704 | 2.2 | C20H38O4 |
| 286 | C25 6,7-Epoxy highly branched isoprenoid | [M-H]- | 365.37889 | 365.37967 | 2.1 | C25H50O |
| 287 | Sannamycin B | [M+Cl]- | 367.21176 | 367.21160 | -0.4 | C15H32N4O4 |
| 288 | Prostaglandin G2 | [M-H]- | 367.21261 | 367.21160 | -2.8 | C20H32O6 |
| 289 | Tetracosanoic acid | [M-H]- | 367.35815 | 367.35735 | -2.2 | C24H48O2 |
| 290 | Dihydro-20-dihydroxy-LTB4 | [M-H]- | 368.22044 | 368.22138 | 2.6 | C20H33O6 |
| 291 | 17-O-Acetylajmaline | [M+H]+ | 369.21727 | 369.21656 | -1.9 | C22H28N2O3 |
| 292 | Fortimicin B | [M+Na]+ | 371.22649 | 371.22616 | -0.9 | C15H32N4O5 |
| 293 | 8-O-Methylsterigmatocystin | [M+Cl]- | 373.04844 | 373.04917 | 2.0 | C19H14O6 |
| 294 | Glucocochlearin | [M-H]- | 374.05850 | 374.05761 | -2.4 | C11H21NO9S2 |
| 295 | Cellobiose-1,5-lactone | [M+Cl]- | 375.06996 | 375.07088 | 2.4 | C12H20O11 |
| 296 | Hydroxy-9,15-dioxoprostenoic acid | [M+Na]+ | 375.21419 | 375.21399 | -0.5 | C20H32O5 |
| 297 | 7-Methylguanosine 5'-phosphate | [M-H]- | 377.07420 | 377.07451 | 0.8 | C11H17N5O8P |
| 298 | Streptidine 6-phosphate | [M+Cl]- | 377.07469 | 377.07451 | -0.5 | C8H19N6O7P |
| 299 | (Cyclopentadienylidene)-androstanol | [M+K]+ | 377.22413 | 377.22479 | 1.8 | C24H34O |
| 300 | (+)-20-methyl-docosanoic acid | [M+Na]+ | 377.33900 | 377.33971 | 1.9 | C23H46O2 |
| 301 | Pentacosanoic acid | [M-H]- | 381.37380 | 381.37387 | 0.2 | C25H50O2 |
| 302 | Glucolepidiin | [M+Cl]- | 382.00387 | 382.00401 | 0.4 | C9H17NO9S2 |
| 303 | 4,21-Dehydrogeissoschizine | [M+Cl]- | 386.14027 | 386.13921 | -2.7 | C21H23N2O3 |
| 304 | Shanzhiside | [M-H]- | 391.12459 | 391.12510 | 1.3 | C16H24O11 |
| 305 | N-stearoyl taurine | [M+H]+ | 392.28291 | 392.28388 | 2.5 | C20H41NO4S |
| 306 | 19-oic-deoxycorticosterone | [M+Cl]- | 394.15525 | 394.15451 | -1.9 | C21H27O5 |
| 307 | O-Acetylcypholophine | [M+Cl]- | 395.17431 | 395.17521 | 2.3 | C20H28N2O4 |
| 308 | Trimethyl-epoxy-methylene-(methylpentenyl)-pentadecene | [M+Cl]- | 395.30862 | 395.30828 | -0.9 | C25H44O |
| 309 | Hexacosanoic acid | [M-H]- | 395.38945 | 395.38872 | -1.9 | C26H52O2 |
| 310 | Quinoline-3-carboxamides | [M+K]+ | 398.07016 | 398.07102 | 2.1 | C21H14FN3O2 |
| 311 | Phytosphingosine-1-P | [M+H]+ | 398.26660 | 398.26682 | 0.5 | C18H40NO6P |
| 312 | N-palmitoyl taurine | [M+K]+ | 402.20749 | 402.20839 | 2.2 | C18H37NO4S |
| 313 | Vomicine | [M+Na]+ | 403.16283 | 403.16345 | 1.5 | C22H24N2O4 |
| 314 | 4,4'-Diaponeurosporene | [M+H]+ | 403.33593 | 403.33558 | -0.9 | C30H42 |
| 315 | Pramanicin | [M+Cl]- | 404.18454 | 404.18424 | -0.7 | C19H31NO6 |
| 316 | Tetranitro-azoxytoluene | [M-H]- | 405.04365 | 405.04456 | 2.2 | C14H10N6O9 |
| 317 | Digalacturonate | [M+Cl]- | 405.04414 | 405.04456 | 1.0 | C12H18O13 |
| 318 | 5'-Prenylhomoeriodictyol | [M+Cl]- | 405.11104 | 405.11051 | -1.3 | C21H22O6 |
| 319 | 3-Deoxyvitamin D3 | [M+K]+ | 407.30746 | 407.30817 | 1.7 | C27H44 |
| 320 | Glucosyloxyanthraquinone | [M+Na]+ | 409.08939 | 409.08902 | -0.9 | C20H18O8 |
| 321 | Bleekerine | [M+H]+ | 409.17580 | 409.17575 | -0.1 | C23H24N2O5 |
| 322 | N-palmitoyl methionine | [M+Na]+ | 410.26994 | 410.27012 | 0.4 | C21H41NO3S |
| 323 | Portulacaxanthin II | [M+K]+ | 413.07456 | 413.07479 | 0.6 | C18H18N2O7 |
| 324 | Resistomycin | [M+K]+ | 415.05785 | 415.05898 | 2.7 | C22H16O6 |
| 325 | (22R)-22,25-dihydroxyvitamin D3 | [M-H]- | 415.32177 | 415.32295 | 2.8 | C27H44O3 |
| 326 | LPA(P-16:0e/0:0) | [M+Na]+ | 417.23765 | 417.23651 | -2.7 | C19H39O6P |
| 327 | 1-hexacosanol | [M+Cl]- | 417.38687 | 417.38739 | 1.3 | C26H54O |
| 328 | 15-hydroxy-pentacosanoic acid | [M+Na]+ | 421.36522 | 421.36418 | -2.5 | C25H50O3 |
| 329 | Oleandolide | [M+K]+ | 425.19361 | 425.19410 | 1.1 | C20H34O7 |
| 330 | Medroxyprogesterone acetate | [M+K]+ | 425.20887 | 425.20974 | 2.0 | C24H34O4 |
| 331 | Hexacosanedioic acid | [M-H]- | 425.36363 | 425.36260 | -2.4 | C26H50O4 |
| 332 | (24R,24'R)-Fucosterol epoxide | [M-H]- | 427.35815 | 427.35817 | 0.0 | C29H48O2 |

| 333 | Fluoro-hydroxypregnenedione acetate | [M+K]+ | 429.18380 | 429.18482 | 2.4 | C23H31FO4 |
| --- | --- | --- | --- | --- | --- | --- |
| 334 | 1(3)-glyceryl-PGF2α | [M+H]+ | 429.28468 | 429.28403 | -1.5 | C23H40O7 |
| 335 | Novobiocic acid | [M+Cl]- | 430.10629 | 430.10707 | 1.8 | C22H21NO6 |
| 336 | N-arachidonoyl D-serine | [M+K]+ | 430.23542 | 430.23652 | 2.6 | C23H37NO4 |
| 337 | Geranylgeranylcysteine | [M+Na]+ | 430.23864 | 430.23833 | -0.7 | C23H37NO3S |
| 338 | Hydroxybenzylkaempferol | [M+K]+ | 431.05276 | 431.05384 | 2.5 | C22H16O7 |
| 339 | Trimethyl-cholestan-ol | [M+H]+ | 431.42474 | 431.42528 | 1.2 | C30H54O |
| 340 | Undecylprodigiosin | [M+K]+ | 432.24117 | 432.24177 | 1.4 | C25H35N3O |
| 341 | Usambarensine | [M+H]+ | 433.23867 | 433.23840 | -0.6 | C29H28N4 |
| 342 | Thiethylperazine | [M+Cl]- | 434.14969 | 434.14929 | -0.9 | C22H29N3S2 |
| 343 | Mundulone | [M+H]+ | 435.18022 | 435.18086 | 1.5 | C26H26O6 |
| 344 | 17-Hydroxypregnenolone sulfate | [M+Na]+ | 435.18118 | 435.18086 | -0.7 | C21H32O6S |
| 345 | Heptadecanoyl carnitine | [M+Na]+ | 436.33973 | 436.34086 | 2.6 | C24H47NO4 |
| 346 | Phlorizin | [M+H]+ | 437.14422 | 437.14505 | 1.9 | C21H24O10 |
| 347 | 2-Phytyl-naphthoquinone | [M+H]+ | 437.34141 | 437.34236 | 2.2 | C30H44O2 |
| 348 | (25R)-cholestane-tetrol | [M+H]+ | 437.36254 | 437.36312 | 1.3 | C27H48O4 |
| 349 | Oxo-22Z-octacosenoic acid | [M+H]+ | 437.39892 | 437.39808 | -1.9 | C28H52O3 |
| 350 | Mycolipenic acid (C29) | [M+H]+ | 437.43531 | 437.43516 | -0.3 | C29H56O2 |
| 351 | Uridine 5'-diphosphate | [M+Cl]- | 438.97160 | 438.97188 | 0.6 | C9H14N2O12P2 |
| 352 | (5Z)-4,4-difluorovitamin D3 | [M+Na]+ | 443.30959 | 443.30923 | -0.8 | C27H42F2O |
| 353 | Octacosanal | [M+Cl]- | 443.40252 | 443.40141 | -2.5 | C28H56O |
| 354 | Tetrahydrofolate | [M-H]- | 444.16371 | 444.16495 | 2.8 | C19H23N7O6 |
| 355 | 1,4-Bis(2-ethylhexyl) sulfosuccinate | [M+Na]+ | 445.22305 | 445.22405 | 2.3 | C20H38O7S |
| 356 | Mycolipanolic acid | [M+Na]+ | 449.39652 | 449.39630 | -0.5 | C27H54O3 |
| 357 | Tricosanoylglycine | [M+K]+ | 450.33440 | 450.33546 | 2.3 | C25H49NO3 |
| 358 | 14-Demethyllanosterol | [M+K]+ | 451.33368 | 451.33459 | 2.0 | C29H48O |
| 359 | Heteroartonin A | [M+H]+ | 453.19078 | 453.19129 | 1.1 | C26H28O7 |
| 360 | Ptilosteroid B | [M+Na]+ | 453.19175 | 453.19129 | -1.0 | C21H34O7S |
| 361 | 3'-Demethylstaurosporine | [M+H]+ | 453.19212 | 453.19129 | -1.8 | C27H24N4O3 |
| 362 | Cinegalline | [M+Na]+ | 453.19961 | 453.19913 | -1.1 | C23H30N2O6 |
| 363 | PA(18:4/0:0) | [M+Na]+ | 453.20126 | 453.20169 | 0.9 | C21H35O7P |
| 364 | Knipholone | [M+Na]+ | 457.08939 | 457.08917 | -0.5 | C24H18O8 |
| 365 | Aloin | [M+K]+ | 457.08954 | 457.08917 | -0.8 | C21H22O9 |
| 366 | Phytyl diphosphate | [M+H]+ | 457.24785 | 457.24810 | 0.5 | C20H42O7P2 |
| 367 | Dihydro-hydroxy-leukotriene E4 | [M+H]+ | 457.24926 | 457.24810 | -2.5 | C23H38NO6S |
| 368 | Hydroxycampestanol | [M+K]+ | 457.34424 | 457.34381 | -0.9 | C28H50O2 |
| 369 | N-oleoyl histidine | [M+K]+ | 458.27795 | 458.27724 | -1.6 | C24H41N3O3 |
| 370 | Epigallocatechin 3-gallate | [M+H]+ | 459.09219 | 459.09314 | 2.1 | C22H18O11 |
| 371 | Isoscoparine | [M-H]- | 461.10894 | 461.10939 | 1.0 | C22H22O11 |
| 372 | N6-(1,2-Dicarboxyethyl)-AMP | [M-H]- | 462.06677 | 462.06552 | -2.7 | C14H18N5O11P |
| 373 | PC(P-15:0/0:0) | [M-H]- | 464.31465 | 464.31400 | -1.4 | C23H48NO6P |
| 374 | Psychotrine | [M+H]+ | 465.27478 | 465.27598 | 2.6 | C28H36N2O4 |
| 375 | Hydroxy-γ-tocotrienol | [M+K]+ | 465.27655 | 465.27598 | -1.2 | C28H42O3 |
| 376 | MG(0:0/24:0/0:0) | [M+Na]+ | 465.39143 | 465.39155 | 0.3 | C27H54O4 |
| 377 | Androsterone 3-glucuronide | [M+H]+ | 467.26394 | 467.26442 | 1.0 | C25H38O8 |
| 378 | Cephaeline | [M+H]+ | 467.29043 | 467.29040 | -0.1 | C28H38N2O4 |
| 379 | Epigallocatechin 3-O-caffeate | [M+H]+ | 469.11292 | 469.11382 | 1.9 | C24H20O10 |
| 380 | Solanocapsine | [M+K]+ | 469.31909 | 469.31787 | -2.6 | C27H46N2O2 |
| 381 | N-arachidonoyl glutamine | [M+K]+ | 471.26197 | 471.26336 | 3.0 | C25H40N2O4 |
| 382 | Hentriacontan-16-one | [M+Na]+ | 473.46929 | 473.47050 | 2.6 | C31H62O |
| 383 | 4-O-α-Cadinylangolensin | [M+H]+ | 477.29994 | 477.30116 | 2.6 | C31H40O4 |
| 384 | 27-Norcholestanehexol | [M+Na]+ | 477.31866 | 477.31973 | 2.2 | C26H46O6 |
| 385 | (-)-Asbestinine 2 | [M+H]+ | 477.32107 | 477.31973 | -2.8 | C28H44O6 |
| 386 | Dihydrophaseic acid 4-O-β-D-glucoside | [M+Cl]- | 479.16895 | 479.16840 | -1.1 | C21H32O10 |
| 387 | 24-methylene-cholesterol sulfate | [M+H]+ | 479.31896 | 479.31960 | 1.3 | C28H46O4S |
| 388 | Difluoro-dihydroxy-dimethylvitamin D3 | [M+H]+ | 481.34878 | 481.34736 | -2.9 | C29H46F2O3 |
| 389 | PA(20:3/0:0) | [M+Na]+ | 483.24821 | 483.24961 | 2.9 | C23H41O7P |
| 390 | PA(22:6/0:0) | [M+H]+ | 483.25062 | 483.24961 | -2.1 | C25H39O7P |
| 391 | 25-dihydroxyvitamin D3 26,23-lactone | [M+K]+ | 483.25073 | 483.24961 | -2.3 | C27H40O5 |
| 392 | Hydrocortisone caproate | [M+Na]+ | 483.27171 | 483.27292 | 2.5 | C27H40O6 |
| 393 | PG(16:1/0:0) | [M+H]+ | 483.27175 | 483.27292 | 2.4 | C22H43O9P |
| 394 | Stigmatellin Y | [M+H]+ | 485.28977 | 485.28950 | -0.5 | C29H40O6 |
| 395 | Triacontatrienoic acid | [M+K]+ | 485.37554 | 485.37632 | 1.6 | C30H54O2 |
| 396 | Hydroxy-(2R)-glutathionyl-1,2-dihydronaphthalene | [M+Cl]- | 486.11072 | 486.10970 | -2.1 | C20H25N3O7S |
| 397 | PC(O-14:1(1E)/0:0) | [M+Cl]- | 486.27568 | 486.27525 | -0.9 | C22H46NO6P |
| 398 | N-stearoyl tyrosine | [M+K]+ | 486.29802 | 486.29752 | -1.0 | C27H45NO4 |
| 399 | (-)-Sanggenone K | [M-H]- | 487.21261 | 487.21399 | 2.8 | C30H32O6 |

| 400 | 15β-Hydroxynicandrin B | [M+H]+ | 487.26903 | 487.26923 | 0.4 | C28H38O7 |
| --- | --- | --- | --- | --- | --- | --- |
| 401 | Myxothiazol A | [M+H]+ | 488.20361 | 488.20290 | -1.5 | C25H33N3O3S2 |
| 402 | Chaksine | [M+K]+ | 489.25861 | 489.25827 | -0.7 | C22H38N6O4 |
| 403 | Hydrocortisone butyrate propionate | [M+H]+ | 489.28468 | 489.28458 | -0.2 | C28H40O7 |
| 404 | C17 sphingosine-1-phosphocholine | [M+K]+ | 489.28542 | 489.28458 | -1.7 | C22H47N2O5P |
| 405 | LysoSM(d18:0) | [M+Na]+ | 489.34278 | 489.34139 | -2.8 | C23H51N2O5P |
| 406 | hentriacontanoic acid | [M+Na]+ | 489.46420 | 489.46415 | -0.1 | C31H62O2 |
| 407 | 3,17-Androstanediol glucuronide | [M+Na]+ | 491.26154 | 491.26186 | 0.7 | C25H40O8 |
| 408 | Ximaosteroid D | [M+H]+ | 491.30033 | 491.30119 | 1.8 | C28H42O7 |
| 409 | Ubiquinone Q4 | [M+K]+ | 493.27147 | 493.27193 | 0.9 | C29H42O4 |
| 410 | Hydroxy-[m-(hydroxy-methylethyl)-benzyloxy]-pentanorvitamin D3 | [M-H]- | 493.33233 | 493.33179 | -1.1 | C32H46O4 |
| 411 | Geneticin | [M-H]- | 495.26717 | 495.26662 | -1.1 | C20H40N4O10 |
| 412 | Difluoro-trihydroxyvitamin D3 | [M+K]+ | 495.26828 | 495.26879 | 1.0 | C26H42F2O4 |
| 413 | Makisterone A | [M+H]+ | 495.33163 | 495.33230 | 1.4 | C28H46O7 |
| 414 | Agrimophol | [M+Na]+ | 497.21459 | 497.21334 | -2.5 | C26H34O8 |
| 415 | N-(4-benzenesulfonamide) arachidonoyl amine | [M+K]+ | 497.22347 | 497.22252 | -1.9 | C26H38N2O3S |
| 416 | 1-Palmitoylglycerophosphocholine | [M+H]+ | 497.34759 | 497.34670 | -1.8 | C24H51NO7P |
| 417 | Carpaine | [M+Na]+ | 501.36628 | 501.36619 | -0.2 | C28H50N2O4 |
| 418 | (-)-Jolkinol A | [M+Na]+ | 503.24041 | 503.24036 | -0.1 | C29H36O6 |
| 419 | Sapelin A | [M+Cl]- | 509.34031 | 509.33921 | -2.2 | C30H50O4 |
| 420 | Luciferyl sulfate | [M+Na]+ | 510.10941 | 510.10929 | -0.2 | C26H21N3O5S |
| 421 | Leinamycin | [M+H]+ | 511.10258 | 511.10222 | -0.7 | C22H26N2O6S3 |
| 422 | Dotriacontatetraenoic acid | [M+K]+ | 511.39119 | 511.39046 | -1.4 | C32H56O2 |
| 423 | 12-O-Octadienoyl-deoxyphorbol 13-acetate | [M+H]+ | 513.28468 | 513.28369 | -1.9 | C30H40O7 |
| 424 | Vitexin 2''-O-(2'''-methylbutyryl) | [M-H]- | 515.15589 | 515.15512 | -1.5 | C26H28O11 |
| 425 | L-Olivosyl-oleandolide | [M-H]- | 515.28617 | 515.28468 | -2.9 | C26H44O10 |
| 426 | Sativanine B | [M-H]- | 517.28203 | 517.28151 | -1.0 | C30H38N4O4 |
| 427 | 27-nor-campestanonol | [M+H]+ | 517.33711 | 517.33557 | -3.0 | C27H48O9 |
| 428 | Cucurbitacin P | [M+H]+ | 521.34728 | 521.34815 | 1.7 | C30H48O7 |
| 429 | Linoleoylglycerophosphocholine | [M+H]+ | 521.34759 | 521.34815 | 1.1 | C26H51NO7P |
| 430 | Dihydroxy-(hydroxymethylphenyl)-didehydrovitamin D3 | [M+H]+ | 521.36254 | 521.36208 | -0.9 | C34H48O4 |
| 431 | Hexafluoro-dihydroxy-didehydrovitamin D3 | [M+H]+ | 523.26414 | 523.26517 | 2.0 | C27H36F6O3 |
| 432 | Malonyldaidzin | [M+Na]+ | 525.10035 | 525.09978 | -1.1 | C24H22O12 |
| 433 | Heterophyllin | [M+Na]+ | 527.20402 | 527.20275 | -2.4 | C30H32O7 |
| 434 | 25-acetoxy-ergosta-3β,5α,6β-triol | [M+Cl]- | 527.35088 | 527.35090 | 0.0 | C30H52O5 |
| 435 | Chlorobactene | [M-H]- | 531.39963 | 531.40022 | 1.1 | C40H52 |
| 436 | Microlenin | [M+K]+ | 533.19361 | 533.19404 | 0.8 | C29H34O7 |
| 437 | DG(14:0/14:1/0:0) | [M+Na]+ | 533.41765 | 533.41643 | -2.3 | C31H58O5 |
| 438 | 11-O-Demethylpradimicinone I | [M-H]- | 534.10418 | 534.10341 | -1.4 | C27H21NO11 |
| 439 | PG(20:3/0:0) | [M+H]+ | 535.30305 | 535.30388 | 1.6 | C26H47O9P |
| 440 | zeta-Carotene | [M-H]- | 539.46223 | 539.46282 | 1.1 | C40H60 |
| 441 | Poly-D-glutamate | [M+Na]+ | 541.17524 | 541.17444 | -1.5 | C20H30N4O12 |
| 442 | Hexafluoro-dihydroxy-tetradehydrovitamin D3 | [M+Na]+ | 543.23043 | 543.23041 | 0.0 | C27H34F6O3 |
| 443 | PE(10:0/10:0) | [M+Na]+ | 546.31662 | 546.31721 | 1.1 | C25H50NO8P |
| 444 | PC(O-1:0/O-18:0) | [M+Na]+ | 546.38940 | 546.39076 | 2.5 | C27H58NO6P |
| 445 | Flavonol 3-O-rutinoside | [M+H]+ | 547.18100 | 547.18144 | 0.8 | C27H30O12 |
| 446 | Trihydroxy-geranyl-(hydroxy-dimethyldihydropyrano)-flavanone | [M+K]+ | 547.20926 | 547.20939 | 0.2 | C30H36O7 |
| 447 | UDP-2-deoxyglucose | [M-H]- | 549.05283 | 549.05294 | 0.2 | C15H24N2O16P2 |
| 448 | Phaseolotoxin | [M+Cl]- | 551.16860 | 551.16976 | 2.1 | C14H33N10O7PS |
| 449 | Terpenoid EA-I | [M+Na]+ | 551.26154 | 551.26312 | 2.9 | C30H40O8 |
| 450 | Trihydroxyecdysone | [M+K]+ | 551.26169 | 551.26312 | 2.6 | C27H44O9 |
| 451 | β-isorenieratane | [M-H]- | 551.55613 | 551.55635 | 0.4 | C40H72 |
| 452 | L-Oleandrosyl-oleandolide | [M+Na]+ | 553.29832 | 553.29766 | -1.2 | C27H46O10 |
| 453 | 3'-Deoxymaysin | [M-H]- | 559.14571 | 559.14501 | -1.3 | C27H28O13 |
| 454 | Pikromycin | [M+Cl]- | 560.29957 | 560.29986 | 0.5 | C28H47NO8 |
| 455 | Baliospermin | [M+H]+ | 563.35785 | 563.35902 | 2.1 | C32H50O8 |
| 456 | Bonafousine | [M+H]+ | 565.31732 | 565.31745 | 0.2 | C35H40N4O3 |
| 457 | Acinospesigenin A | [M+Cl]- | 565.33014 | 565.32960 | -1.0 | C32H50O6 |
| 458 | N-(tetradecanoyl)-deoxysphing-4-enine-1-sulfonate | [M-H]- | 572.43542 | 572.43676 | 2.3 | C32H63NO5S |
| 459 | 1-(O-α-D-glucopyranosyl)-(1,3R,25R)-hexacosanetriol | [M+H]+ | 577.46740 | 577.46894 | 2.7 | C32H64O8 |
| 460 | dTDP-L-epivancosamine | [M+Cl]- | 580.08696 | 580.08789 | 1.6 | C17H29N3O13P2 |
| 461 | PS(22:0/0:0) | [M+H]+ | 582.37655 | 582.37682 | 0.5 | C28H56NO9P |
| 462 | Pubescenol | [M+H]+ | 585.26942 | 585.27047 | 1.8 | C32H40O10 |
| 463 | Bilirubin | [M+H]+ | 585.27076 | 585.27047 | -0.5 | C33H36N4O6 |
| 464 | DG(14:0/18:3/0:0) | [M+Na]+ | 585.44895 | 585.44890 | -0.1 | C35H62O5 |

| 465 | SQMG(16:1(9Z)/0:0) | [M+Cl]- | 588.23766 | 588.23837 | 1.2 | C25H45O11S |
| --- | --- | --- | --- | --- | --- | --- |
| 466 | PG(21:0/0:0) | [M+K]+ | 593.32153 | 593.32231 | 1.3 | C27H55O9P |
| 467 | 13-Deoxytedanolide | [M+H]+ | 595.34767 | 595.34930 | 2.7 | C32H50O10 |
| 468 | L-Urobilin | [M+H]+ | 595.34901 | 595.34930 | 0.5 | C33H46N4O6 |
| 469 | Dalpanol O-glucoside | [M+Na]+ | 597.19425 | 597.19343 | -1.4 | C29H34O12 |
| 470 | DG(15:0/18:4/0:0) | [M+Na]+ | 597.44895 | 597.45071 | 3.0 | C36H62O5 |
| 471 | PA(12:0/15:1) | [M+Na]+ | 599.36833 | 599.36793 | -0.7 | C30H57O8P |
| 472 | 4-Ketomyxol | [M+H]+ | 599.40949 | 599.41058 | 1.8 | C40H54O4 |
| 473 | PA(12:0/14:1(9Z)) | [M+K]+ | 601.32661 | 601.32728 | 1.1 | C29H55O8P |
| 474 | (acetoxymethylphenyl)-dihydroxy-didehydrovitamin D3 | [M+Cl]- | 601.32898 | 601.32728 | -2.8 | C36H50O5 |
| 475 | PA(12:0/15:0) | [M+Na]+ | 601.38398 | 601.38540 | 2.4 | C30H59O8P |
| 476 | OH-Diaponeurosporene glucoside ester | [M+Na]+ | 605.38126 | 605.38261 | 2.2 | C36H54O6 |
| 477 | DG(15:0/18:0/0:0) | [M+Na]+ | 605.51155 | 605.51065 | -1.5 | C36H70O5 |
| 478 | 7''-O-Phosphohygromycin | [M-H]- | 606.19169 | 606.19209 | 0.7 | C20H38N3O16P |
| 479 | PS(22:2(13Z,16Z)/0:0) | [M+Cl]- | 612.30737 | 612.30742 | 0.1 | C28H52NO9P |
| 480 | Uvarinol | [M+K]+ | 613.16231 | 613.16341 | 1.8 | C36H30O7 |
| 481 | PI(O-20:0/0:0) | [M-H]- | 613.37222 | 613.37338 | 1.9 | C29H59O11P |
| 482 | PA(O-16:0/12:0) | [M+Cl]- | 613.40054 | 613.40000 | -0.9 | C31H63O7P |
| 483 | Dihydroxyneurosporene | [M+K]+ | 613.43814 | 613.43789 | -0.4 | C40H62O2 |
| 484 | Debromoaplysiatoxin | [M+Na]+ | 615.31397 | 615.31281 | -1.9 | C32H48O10 |
| 485 | Kurilensoside H | [M+H]+ | 615.37389 | 615.37334 | -0.9 | C32H54O11 |
| 486 | 2'-Norberbamunine | [M+Cl]- | 617.24239 | 617.24356 | 1.9 | C35H38N2O6 |
| 487 | PA(12:0/18:2) | [M+H]+ | 617.41768 | 617.41593 | -2.8 | C33H61O8P |
| 488 | Leukotriene C4 | [M-H]- | 624.29602 | 624.29472 | -2.1 | C30H47N3O9S |
| 489 | all-trans-Hexaprenyl diphosphate | [M+K]+ | 625.28199 | 625.28194 | -0.1 | C30H52O7P2 |
| 490 | Kolaflavanone | [M+K]+ | 627.08994 | 627.09099 | 1.7 | C31H24O12 |
| 491 | PA(14:1/14:1) | [M+K]+ | 627.34226 | 627.34411 | 2.9 | C31H57O8P |
| 492 | PA(12:0/17:1) | [M+Na]+ | 627.39963 | 627.40118 | 2.5 | C32H61O8P |
| 493 | PA(13:0/18:4) | [M+H]+ | 627.40203 | 627.40118 | -1.4 | C34H59O8P |
| 494 | UDP-6-sulfoquinovose | [M-H]- | 628.00182 | 628.00267 | 1.4 | C15H23N2O19P2S |
| 495 | Perhydroazepino-N-carbonyl-L-Leu-D-Trp-D-Trp | [M+H]+ | 629.34460 | 629.34380 | -1.3 | C35H44N6O5 |
| 496 | PA(P-16:0/16:1) | [M-H]- | 629.45516 | 629.45518 | 0.0 | C35H67O7P |
| 497 | Delphinine | [M+Cl]- | 634.27883 | 634.27809 | -1.2 | C33H45NO9 |
| 498 | PA(O-16:0/14:1(9Z)) | [M+K]+ | 643.40995 | 643.40833 | -2.5 | C33H65O7P |
| 499 | Cladrin 7-O-laminaribioside | [M+Na]+ | 645.17899 | 645.18058 | 2.5 | C29H34O15 |
| 500 | 6-Methoxykaempferol 3-rhamnoside-7-(4'''-acetylrhamnoside) | [M+H]+ | 651.19196 | 651.19290 | 1.4 | C30H34O16 |
| 501 | PA(13:0/20:5) | [M+H]+ | 653.41768 | 653.41830 | 0.9 | C36H61O8P |
| 502 | DG(17:2/22:5/0:0 | [M+H]+ | 653.51395 | 653.51352 | -0.7 | C42H68O5 |
| 503 | 12-O-Tetradecanoylphorbol 13-acetate | [M+K]+ | 655.36068 | 655.35907 | -2.5 | C36H56O8 |
| 504 | PE(12:0/15:1(9Z)) | [M+K]+ | 658.38446 | 658.38349 | -1.5 | C32H62NO8P |
| 505 | PA(18:0/12:0) | [M+K]+ | 659.40487 | 659.40537 | 0.8 | C33H65O8P |
| 506 | PE(O-16:0/12:0) | [M+K]+ | 660.43650 | 660.43507 | -2.2 | C33H68NO7P |
| 507 | PE-NMe(O-14:0/O-14:0) | [M+K]+ | 660.47289 | 660.47113 | -2.7 | C34H72NO6P |
| 508 | PA(O-20:0/14:1) | [M+H]+ | 661.51667 | 661.51779 | 1.7 | C37H73O7P |
| 509 | DG(14:0/22:1/0:0) | [M+K]+ | 661.51678 | 661.51779 | 1.5 | C39H74O5 |
| 510 | Dauricine | [M+K]+ | 663.28310 | 663.28254 | -0.8 | C38H44N2O6 |
| 511 | PG(14:1/14:1) | [M+H]+ | 663.42316 | 663.42124 | -2.9 | C34H63O10P |
| 512 | Amphibine B | [M+H]+ | 666.36500 | 666.36334 | -2.5 | C39H47N5O5 |
| 513 | DG(16:0/22:4/0:0) | [M+Na]+ | 667.52720 | 667.52904 | 2.8 | C41H72O5 |
| 514 | DG(18:1/22:6/0:0) | [M+H]+ | 667.52960 | 667.52904 | -0.8 | C43H70O5 |
| 515 | Enterochelin | [M-H]- | 668.13694 | 668.13840 | 2.2 | C30H27N3O15 |
| 516 | PI(22:4/0:0) | [M+Na]+ | 671.31668 | 671.31596 | -1.1 | C31H53O12P |
| 517 | Thapsigargin | [M+Na]+ | 673.31945 | 673.31774 | -2.5 | C34H50O12 |
| 518 | Hexadecanoyl-(octadecenoyl)-sn-glycero-phosphate | [M+H]+ | 675.49593 | 675.49609 | 0.2 | C37H71O8P |
| 519 | PC(O-14:0/15:0) | [M-H]- | 676.52866 | 676.52998 | 1.9 | C37H76NO7P |
| 520 | PA(P-16:0/20:5) | [M-H]- | 677.45516 | 677.45358 | -2.3 | C39H67O7P |
| 521 | Trihydroxy-trimethoxyflavone galactosyl-glucoside | [M-H]- | 683.18289 | 683.18291 | 0.0 | C30H36O18 |
| 522 | Cyanidin 3-(6''-malonylsambubioside) | [M+Na]+ | 690.14026 | 690.14017 | -0.1 | C29H31O18 |
| 523 | PG(12:0/15:0) | [M+K]+ | 691.39469 | 691.39400 | -1.0 | C33H65O10P |
| 524 | Thalicarpine | [M-H]- | 695.33379 | 695.33501 | 1.8 | C41H48N2O8 |
| 525 | Adenosylhopane | [M+Cl]- | 696.46249 | 696.46426 | 2.5 | C40H63N5O3 |
| 526 | PG(12:0/16:1) | [M+K]+ | 703.39469 | 703.39502 | 0.5 | C34H65O10P |
| 527 | PG(P-16:0/16:1) | [M-H]- | 703.49194 | 703.49217 | 0.3 | C38H73O9P |
| 528 | PA(14:0/20:3) | [M+K]+ | 709.42052 | 709.42063 | 0.2 | C37H67O8P |
| 529 | PA(18:4/18:4) | [M+Na]+ | 711.39963 | 711.39822 | -2.0 | C39H61O8P |
| 530 | Premithramycin A2' | [M+K]+ | 713.18423 | 713.18361 | -0.9 | C33H38O15 |

| 531 | PE-NMe(O-16:0/O-16:0) | [M+K]+ | 716.53549 | 716.53584 | 0.5 | C38H80NO6P |
| --- | --- | --- | --- | --- | --- | --- |
| 532 | Elatine | [M+Na]+ | 717.33577 | 717.33675 | 1.4 | C38H50N2O10 |
| 533 | Nigericin | [M-H]- | 723.46889 | 723.46893 | 0.1 | C40H68O11 |
| 534 | PA(16:1/22:2) | [M-H]- | 725.51268 | 725.51157 | -1.5 | C41H75O8P |
| 535 | Prunin 3'',6''-di-p-coumarate | [M+H]+ | 727.20213 | 727.20266 | 0.7 | C39H34O14 |
| 536 | PA(15:1/22:6) | [M+Na]+ | 727.43093 | 727.43198 | 1.4 | C40H65O8P |
| 537 | Pelargonidin 3-sambubioside-5-glucoside | [M+H]+ | 728.21583 | 728.21464 | -1.6 | C32H39O19 |
| 538 | PA(15:0/22:6) | [M+Na]+ | 729.44658 | 729.44547 | -1.5 | C40H67O8P |
| 539 | PG(P-16:0/15:1) | [M+K]+ | 729.44673 | 729.44547 | -1.7 | C37H71O9P |
| 540 | PI(12:0/13:0) | [M+H]+ | 730.45010 | 730.45196 | 2.5 | C34H68NO13P |
| 541 | PA(15:1/22:4) | [M+Na]+ | 731.46223 | 731.46109 | -1.6 | C40H69O8P |
| 542 | PG(O-16:0/15:1) | [M+K]+ | 731.46238 | 731.46109 | -1.8 | C37H73O9P |
| 543 | PE(14:0/20:5) | [M+Na]+ | 732.45748 | 732.45684 | -0.9 | C39H68NO8P |
| 544 | PS(O-16:0/14:0) | [M+K]+ | 732.45763 | 732.45684 | -1.1 | C36H72NO9P |
| 545 | PE(14:1/22:6) | [M-H]- | 732.46098 | 732.46300 | 2.8 | C41H68NO8P |
| 546 | 2-demethylmenaquinone-8 | [M+Cl]- | 737.50698 | 737.50679 | -0.3 | C50H70O2 |
| 547 | PE(15:0/18:3) | [M+K]+ | 738.44706 | 738.44646 | -0.8 | C38H70NO8P |
| 548 | PI(O-16:0/12:0) | [M-H]- | 739.47669 | 739.47517 | -2.1 | C37H73O12P |
| 549 | PG(12:0/19:1) | [M+K]+ | 745.44164 | 745.44082 | -1.1 | C37H71O10P |
| 550 | Pfaffoside A | [M-H]- | 747.39612 | 747.39499 | -1.5 | C40H60O13 |
| 551 | PA(O-16:0/22:4) | [M+K]+ | 749.48820 | 749.48851 | 0.4 | C41H75O7P |
| 552 | PA(15:1/22:2) | [M+K]+ | 751.46747 | 751.46751 | 0.1 | C40H73O8P |
| 553 | PG(17:0/14:1(9Z)) | [M+Cl]- | 758.47444 | 758.47430 | -0.2 | C37H74NO10P |
| 554 | Pentahydroxychalcone 4'-O-(2''-O-caffeoyl-6''-O-p-  coumaroylglucoside) | [M+H]+ | 759.19196 | 759.19073 | -1.6 | C39H34O16 |
| 555 | SM(d18:1/20:0) | [M+H]+ | 759.63745 | 759.63880 | 1.8 | C43H87N2O6P |
| 556 | Apigenin 7-(2'',6''-di-p-coumarylglucoside) | [M+K]+ | 763.14237 | 763.14322 | 1.1 | C39H32O14 |
| 557 | PA(19:0/22:6) | [M+H]+ | 763.52723 | 763.52785 | 0.8 | C44H75O8P |
| 558 | PG(O-16:0/20:1) | [M+H]+ | 763.58475 | 763.58502 | 0.4 | C42H83O9P |
| 559 | Fumonisin A1 | [M+H]+ | 764.40631 | 764.40694 | 0.8 | C36H61NO16 |
| 560 | Amaranthin | [M+K]+ | 765.13874 | 765.13967 | 1.2 | C30H34N2O19 |
| 561 | Triiodothyronine sulfate | [M+Cl]- | 765.71627 | 765.71443 | -2.4 | C15H12I3NO7S |
| 562 | 1-(ladderane-hexanoyl)-(ladderane-octanyl)-  glycerophosphoethanolamine | [M+Na]+ | 766.47821 | 766.47948 | 1.7 | C43H70NO7P |
| 563 | PC(14:0/18:3) | [M+K]+ | 766.47836 | 766.47948 | 1.5 | C40H74NO8P |
| 564 | PC(15:0/P-18:0) | [M+Cl]- | 766.55229 | 766.55042 | -2.4 | C41H82NO7P |
| 565 | PG(13:0/20:4) | [M+K]+ | 767.42599 | 767.42683 | 1.1 | C39H69O10P |
| 566 | Tautomycin | [M+H]+ | 767.45762 | 767.45783 | 0.3 | C41H66O13 |
| 567 | PA(17:1/22:4) | [M+K]+ | 775.46747 | 775.46674 | -0.9 | C42H73O8P |
| 568 | PA(18:1/22:2) | [M+Na]+ | 777.54048 | 777.54126 | 1.0 | C43H79O8P |
| 569 | PA(20:2/22:4) | [M+H]+ | 777.54288 | 777.54126 | -2.1 | C45H77O8P |
| 570 | PPA(18:1/18:1) | [M+H]+ | 781.47791 | 781.47813 | 0.3 | C39H74O11P2 |
| 571 | Deoxymyxol 2'-(2,4-di-O-methyl-α-L-fucoside) | [M+K]+ | 781.48040 | 781.47813 | -2.9 | C48H70O6 |
| 572 | PS(O-16:0/19:1) | [M+Na]+ | 784.54629 | 784.54860 | 2.9 | C41H80NO9P |
| 573 | PE(18:0/20:4(12OH[S])) | [M+H]+ | 784.54870 | 784.54860 | -0.1 | C43H78NO9P |
| 574 | Phalloidin | [M-H]- | 787.30905 | 787.30795 | -1.4 | C35H48N8O11S |
| 575 | PA(22:0/20:0) | [M-H]- | 787.62223 | 787.62441 | 2.8 | C45H89O8P |
| 576 | SM(d18:0/22:1) | [M+H]+ | 787.66875 | 787.67006 | 1.7 | C45H91N2O6P |
| 577 | Tetranactin | [M+H]+ | 793.50965 | 793.51092 | 1.6 | C44H72O12 |
| 578 | PS(15:1/22:4) | [M-H]- | 794.49776 | 794.49662 | -1.4 | C43H74NO10P |
| 579 | PG(16:0/20:3) | [M+Na]+ | 795.51466 | 795.51417 | -0.6 | C42H77O10P |
| 580 | PC(O-16:0/19:0) | [M+Cl]- | 796.59924 | 796.59811 | -1.4 | C43H88NO7P |
| 581 | Myxol 2'-(2,4-di-O-methyl-α-L-fucoside) | [M+K]+ | 797.47531 | 797.47677 | 1.8 | C48H70O7 |
| 582 | PI(O-18:0/14:0) | [M+H]+ | 797.55384 | 797.55228 | -2.0 | C41H81O12P |
| 583 | Epimedoside | [M+K]+ | 799.22101 | 799.22065 | -0.4 | C37H44O17 |
| 584 | Hyaluronan | [M+Na]+ | 799.22271 | 799.22065 | -2.6 | C28H44N2O23 |
| 585 | SM(d18:0/20:0) | [M+K]+ | 799.60898 | 799.60849 | -0.6 | C43H89N2O6P |
| 586 | SM(d17:1/24:1) | [M+H]+ | 799.66875 | 799.66830 | -0.6 | C46H91N2O6P |
| 587 | PS(O-18:0/20:3) | [M+H]+ | 800.58000 | 800.58013 | 0.2 | C44H82NO9P |
| 588 | Quercetin 3-(2'''-feruloylsophoroside) | [M+H]+ | 803.20292 | 803.20377 | 1.1 | C37H38O20 |
| 589 | Stevioside | [M-H]- | 803.37069 | 803.36896 | -2.2 | C38H60O18 |
| 590 | SM(d18:0/23:0) | [M+H]+ | 803.70005 | 803.69956 | -0.6 | C46H95N2O6P |
| 591 | PI(O-16:0/15:0) | [M+Na]+ | 805.52014 | 805.52087 | 0.9 | C40H79O12P |
| 592 | PI(P-16:0/17:2) | [M+H]+ | 805.52254 | 805.52087 | -2.1 | C42H77O12P |
| 593 | PE(18:2/22:6) | [M+Na]+ | 810.50443 | 810.50542 | 1.2 | C45H74NO8P |
| 594 | PS(O-18:0/18:3) | [M+K]+ | 810.50458 | 810.50542 | 1.0 | C42H78NO9P |
| 595 | PE(20:5/22:6) | [M+H]+ | 810.50683 | 810.50542 | -1.7 | C47H72NO8P |

| 596 | N,N'-Diacetylchitobiosyldiphosphodolichol | [M+K]+ | 819.21146 | 819.21313 | 2.0 | C28H50N2O19P2 |
| --- | --- | --- | --- | --- | --- | --- |
| 597 | PI(O-16:0/15:1) | [M+K]+ | 819.47842 | 819.48079 | 2.9 | C40H77O12P |
| 598 | PI(O-20:0/14:0) | [M-H]- | 823.57059 | 823.57228 | 2.1 | C43H85O12P |
| 599 | PC(22:0/P-18:0) | [M+H]+ | 830.69972 | 830.69848 | -1.5 | C48H96NO7P |
| 600 | Leucomycin A13 | [M+Cl]- | 834.44121 | 834.44152 | 0.4 | C41H69NO14 |
| 601 | Roxithromycin | [M+H]+ | 837.53185 | 837.52993 | -2.3 | C41H76N2O15 |
| 602 | PE(20:2/22:6) | [M+Na]+ | 838.53573 | 838.53502 | -0.8 | C47H78NO8P |
| 603 | PG(19:0/22:4) | [M-H]- | 839.58076 | 839.57986 | -1.1 | C47H85O10P |
| 604 | PI(O-18:0/17:0) | [M+H]+ | 839.60079 | 839.60019 | -0.7 | C44H87O12P |
| 605 | PI(13:0/20:3) | [M+Na]+ | 841.48375 | 841.48379 | 0.0 | C42H75O13P |
| 606 | PI(13:0/22:6) | [M+H]+ | 841.48616 | 841.48379 | -2.8 | C44H73O13P |
| 607 | PG(O-18:0/22:1) | [M+Na]+ | 841.62929 | 841.62764 | -2.0 | C46H91O9P |
| 608 | PG(P-20:0/22:2) | [M+H]+ | 843.64735 | 843.64816 | 1.0 | C48H91O9P |
| 609 | Rifabutin | [M-H]- | 845.43423 | 845.43649 | 2.7 | C46H62N4O11 |
| 610 | PGP(18:3/18:3) | [M-H]- | 845.43754 | 845.43649 | -1.2 | C42H72O13P2 |
| 611 | PG(19:0/22:2) | [M+H]+ | 845.62661 | 845.62820 | 1.9 | C47H89O10P |
| 612 | SM(d18:1/24:0) | [M+Cl]- | 850.67000 | 850.67229 | 2.7 | C47H96N2O6P |
| 613 | PG(18:1/22:2) | [M+Na]+ | 851.57726 | 851.57514 | -2.5 | C46H85O10P |
| 614 | Hydroxymethylbilane | [M+H]+ | 855.29307 | 855.29375 | 0.8 | C40H46N4O17 |
| 615 | Cyanidin 3-(6''-malonylsophoroside)-5-glucoside | [M-H]- | 858.20715 | 858.20607 | -1.3 | C36H43O24 |
| 616 | β-hydroarchaetidylglycerol | [M+K]+ | 861.63453 | 861.63271 | -2.1 | C46H95O9P |
| 617 | 2-Methylacetoacetyl-CoA | [M-H]- | 864.14471 | 864.14420 | -0.6 | C26H42N7O18P3S |
| 618 | PS(18:3/22:6) | [M+Cl]- | 864.45879 | 864.45685 | -2.2 | C46H72NO10P |
| 619 | PI(14:0/22:1) | [M+H]+ | 865.58006 | 865.57908 | -1.1 | C45H85O13P |
| 620 | PI(15:0/20:3) | [M+Na]+ | 869.51505 | 869.51652 | 1.7 | C44H79O13P |
| 621 | PI(15:0/22:6) | [M+H]+ | 869.51746 | 869.51652 | -1.1 | C46H77O13P |
| 622 | Helieianeoside C | [M-H]- | 873.23063 | 873.23236 | 2.0 | C37H46O24 |
| 623 | Glucosylceramide (d18:1/26:0) | [M+Cl]- | 874.69082 | 874.69161 | 0.9 | C50H97NO8 |
| 624 | Didodecanoyl-sn-glycero-3-cytidine-5'-diphosphate | [M+Cl]- | 876.35849 | 876.36046 | 2.2 | C36H65N3O15P2 |
| 625 | PG(21:0/22:0) | [M+H]+ | 877.68921 | 877.69053 | 1.5 | C49H97O10P |
| 626 | Palargonidin 3-(6''-ferulylglucoside)-5-(6'''-malonylglucoside) | [M+Na]+ | 878.22399 | 878.22394 | -0.1 | C41H43O20 |
| 627 | PGP(16:0/22:4) | [M+H]+ | 879.51469 | 879.51514 | 0.5 | C44H80O13P2 |
| 628 | PC(o-22:0/20:4(8Z,11Z,14Z,17Z)) | [M+Cl]- | 886.64619 | 886.64428 | -2.2 | C50H94NO7P |
| 629 | PI(17:2/22:4) | [M+H]+ | 897.54876 | 897.55006 | 1.5 | C48H81O13P |
| 630 | PI(P-20:0/17:2) | [M+K]+ | 899.54102 | 899.54267 | 1.8 | C46H85O12P |
| 631 | Megalomicin A | [M+Na]+ | 899.54509 | 899.54267 | -2.7 | C44H80N2O15 |
| 632 | PI(17:0/22:0) | [M-H]- | 907.62810 | 907.62718 | -1.0 | C48H93O13P |
| 633 | PG(22:2/22:6)) | [M+Cl]- | 909.54179 | 909.54208 | 0.3 | C50H83O10P |
| 634 | Kaempferol 3-(6'''-rhamnosyl-2'''-(6-malyl-glucosyl)-glucoside) | [M+K]+ | 911.18541 | 911.18419 | -1.3 | C37H44O24 |
| 635 | (3S)-3-Carboxy-3-hydroxypropanoyl-CoA | [M+Cl]- | 918.09557 | 918.09566 | 0.1 | C25H40N7O20P3S |
| 636 | Precorrin 5 | [M-H]- | 921.34112 | 921.34190 | 0.8 | C45H54N4O17 |
| 637 | Galabiosylceramide (d18:1/18:0) | [M+Cl]- | 924.61844 | 924.61571 | -3.0 | C48H91NO13 |
| 638 | PI(O-20:0/22:1) | [M+H]+ | 935.69469 | 935.69240 | -2.4 | C51H99O12P |
| 639 | PI(O-20:0/22:0) | [M+H]+ | 937.71034 | 937.71201 | 1.8 | C51H101O12P |
| 640 | β-D-Mannosylphosphodecaprenol | [M-H]- | 939.64844 | 939.64688 | -1.7 | C56H93O9P |
| 641 | Acetylmalonylawobanin | [M+K]+ | 940.16702 | 940.16922 | 2.3 | C41H41O23 |
| 642 | 6-Carboxyhexanoyl-CoA | [M+Cl]- | 944.14760 | 944.14905 | 1.5 | C28H46N7O19P3S |
| 643 | Kaempferol 3-[2'''-(E)-ferulylsophoroside]-7-glucoside | [M+H]+ | 949.26083 | 949.26029 | -0.6 | C43H48O24 |
| 644 | PI(18:0/22:4) | [M+Cl]- | 949.55783 | 949.55797 | 0.1 | C49H87O13P |
| 645 | Salvianin | [M+Na]+ | 952.18800 | 952.18933 | 1.4 | C42H41O24 |
| 646 | TG(17:1/20:4/20:4) | [M+K]+ | 953.69950 | 953.69911 | -0.4 | C60H98O6 |
| 647 | Phenylitaconyl-CoA | [M+H]+ | 956.16983 | 956.17239 | 2.7 | C32H44N7O19P3S |
| 648 | PI(22:2/22:4) | [M+H]+ | 967.62701 | 967.62768 | 0.7 | C53H91O13P |
| 649 | PI(O-20:0/22:2) | [M+Cl]- | 967.64117 | 967.63848 | -2.8 | C51H97O12P |
| 650 | 4,8-Dimethylnonanoyl-CoA | [M+Cl]- | 970.23603 | 970.23344 | -2.7 | C32H56N7O17P3S |
| 651 | Guanosine pentaphosphate adenosine | [M+K]+ | 970.97267 | 970.97095 | -1.8 | C20H29N10O23P5 |
| 652 | TG(20:5/20:5/20:5) | [M+Cl]- | 979.65879 | 979.66118 | 2.4 | C63H92O6 |
| 653 | TG(17:0/20:5/22:3) | [M+K]+ | 983.74645 | 983.74461 | -1.9 | C62H104O6 |
| 654 | TG(18:3/20:4/22:6) | [M+K]+ | 987.68385 | 987.68165 | -2.2 | C63H96O6 |
| 655 | PIP2(16:0/18:2) | [M-H]- | 993.45121 | 993.44876 | -2.5 | C43H81O19P3 |
| 656 | PC(20:4/32:0) | [M-H]- | 1004.80528 | 1004.80374 | -1.5 | C60H112NO8P |
| 657 | PC(34:0/16:0) | [M+Cl]- | 1020.81326 | 1020.81314 | -0.1 | C58H116NO8P |
| 658 | TG(22:5/20:5/22:6) | [M+Na]+ | 1021.72556 | 1021.72392 | -1.6 | C67H98O6 |
| 659 | Farnesoyl-CoA | [M+K]+ | 1024.24543 | 1024.24596 | 0.5 | C36H58N7O17P3S |
| 660 | Temsirolimus | [M+H]+ | 1030.60976 | 1030.60797 | -1.7 | C56H87NO16 |

| 661 | β-D-Glucosyl-1-phosphoundecaprenol | [M+Na]+ | 1031.70754 | 1031.70952 | 1.9 | C61H101O9P |
| --- | --- | --- | --- | --- | --- | --- |
| 662 | Glochiin C1 | [M+Cl]- | 1083.14510 | 1083.14673 | 1.5 | C48H40O27 |
| 663 | Leucodelphinidin 4-O-[2,4-bisgalloyl-6-(3-galloylgalloyl)-β-D-  glucopyranoside] | [M+H]+ | 1093.17280 | 1093.17012 | -2.5 | C49H40O29 |
| 664 | PIP3(18:0/18:1) | [M-H]- | 1103.46449 | 1103.46187 | -2.4 | C45H88O22P4 |
| 665 | Ciguatoxin I | [M+Cl]- | 1145.54573 | 1145.54736 | 1.4 | C60H86O19 |
| a Cer: Ceramide; GalCer: Galactosylceramide; GlcCer: Glucosylceramide; ; LacCer: Lactosylceramide; MG: Monoacylglycerol; DG: Diacylglycerol; TG: Triacylglycerol; MGDG: Monoacyldiacylglycerol; PA: Phosphatidic acid; PC: Phosphatidylcholine; PE: Phosphatidylethanolamine; PG(P): Glycerophospholipids; PI: Phosphatidylinositol; PS: Phosphatidylserine; SM: Sphingomyelin; CDP: Cytidine diphosphate; UDP: Uridine diphospate; SQMG:  sulfoquinovosylmonoacylglycerols | | | | | | |
