# Supplementary material for: Satureja montana L. Essential Oils: Chemical Profiles/Phytochemical Screening, Antimicrobial Activity and O/W NanoEmulsion Formulations
Source: Pharmaceutics. 2019 Dec 19;12(1):7. doi: 10.3390/pharmaceutics12010007 (PMC7022231; doi:10.3390/pharmaceutics12010007)
Supplement: Supplementary file 1 [file pharmaceutics-12-00007-s001.zip › pharmaceutics-623925-SI-final/TableS2.docx]

Table S2. SEO 2 Untargeted ESI FT-ICR Annotations.

| No. | Compound (M)^a^ | Ion | Theor. m/z | Exp. m/z | Δppm | Formula |
| --- | --- | --- | --- | --- | --- | --- |
| 1 | Phosphinic acid | [M-H]- | 64.97979 | 64.97962 | -2.6 | H3PO2 |
| 2 | Thiourea | [M-H]- | 75.00224 | 75.00227 | 0.4 | CH4N2S |
| 3 | Piperazine | [M+H]+ | 87.09167 | 87.09161 | -0.7 | C4H10N2 |
| 4 | Lactic acid | [M-H]- | 89.02442 | 89.02442 | 0.0 | C3H6O3 |
| 5 | Hexenal | [M-H]- | 99.08154 | 99.08150 | -0.4 | C6H12O |
| 6 | Choline | [M]+ | 104.10699 | 104.10718 | 1.8 | C5H14NO |
| 7 | 2-Aminotoluene | [M+H]+ | 108.08078 | 108.08066 | -1.1 | C7H9N |
| 8 | Cytosine | [M-H]- | 110.03599 | 110.03590 | -0.8 | C4H5N3O |
| 9 | 2-Methylpropanal oxime | [M+Na]+ | 110.05763 | 110.05732 | -2.9 | C4H9NO |
| 10 | 5-Methyl-2-furaldehyde | [M+H]+ | 111.04406 | 111.04399 | -0.6 | C6H6O2 |
| 11 | 2,4-heptadienal | [M+H]+ | 111.08044 | 111.08067 | 2.1 | C7H10O |
| 12 | 3-Amino-2-piperidone | [M-H]- | 113.07204 | 113.07214 | 0.9 | C5H10N2O |
| 13 | ε-Caprolactam | [M+H]+ | 114.09134 | 114.09111 | -2.0 | C6H11NO |
| 14 | 5-Aminopentanamide | [M+H]+ | 117.10224 | 117.10228 | 0.3 | C5H12N2O |
| 15 | 3-Hydroxybenzaldehyde | [M-H]- | 121.02950 | 121.02970 | 1.6 | C7H6O2 |
| 16 | 1-Phenylethylamine | [M+H]+ | 122.09643 | 122.09616 | -2.2 | C8H11N |
| 17 | Octanal | [M-H]- | 127.11284 | 127.11298 | 1.1 | C8H16O |
| 18 | 2-heptenedial | [M+H]+ | 127.07536 | 127.07499 | -2.9 | C7H10O2 |
| 19 | Octylamine | [M+H]+ | 130.15903 | 130.15940 | 2.9 | C8H19N |
| 20 | (3R)-β-Leucine | [M+H]+ | 132.10191 | 132.10199 | 0.6 | C6H13NO2 |
| 21 | Chavicol | [M+H]+ | 135.08044 | 135.08050 | 0.4 | C9H10O |
| 22 | Cymene | [M+H]+ | 135.11683 | 135.11649 | -2.5 | C10H14 |
| 23 | Terpinene | [M+H]+ | 137.13248 | 137.13248 | 0.0 | C10H16 |
| 24 | 2,4,6-Triaminotoluene | [M+H]+ | 138.10257 | 138.10237 | -1.5 | C7H11N3 |
| 25 | Pinidine | [M+H]+ | 140.14338 | 140.14378 | 2.9 | C9H17N |
| 26 | (-)-Hygrine | [M+H]+ | 142.12264 | 142.12236 | -2.0 | C8H15NO |
| 27 | 4-Oxocyclohexanecarboxylate | [M+H]+ | 143.07027 | 143.07066 | 2.7 | C7H10O3 |
| 28 | o-Benzosemiquinone | [M+Cl]- | 143.99836 | 143.99798 | -2.6 | C6H5O2 |
| 29 | Tetrahydrothiopheneacetic acid | [M-H]- | 145.03287 | 145.03255 | -2.2 | C6H10O2S |
| 30 | 1-(3-Aminopropyl)-4-aminobutanal | [M+H]+ | 145.13354 | 145.13372 | 1.2 | C7H16N2O |
| 31 | O-Carbamoyl-L-serine | [M-H]- | 147.04113 | 147.04096 | -1.2 | C4H8N2O4 |
| 32 | Actinidine | [M+H]+ | 148.11208 | 148.11197 | -0.7 | C10H13N |
| 33 | Carvacrol | [M-H]- | 149.09719 | 149.09716 | -0.2 | C10H14O |
| 34 | Triethanolamine | [M+H]+ | 150.11247 | 150.11230 | -1.1 | C6H15NO3 |
| 35 | 2-Methylacetoacetic acid | [M+Cl]- | 151.01675 | 151.01707 | 2.1 | C5H8O3 |
| 36 | 1-Oxa-2-oxo-3-methylcycloheptane | [M+Na]+ | 151.07295 | 151.07320 | 1.7 | C7H12O2 |
| 37 | Octane | [M+K]+ | 153.10401 | 153.10392 | -0.6 | C8H18 |
| 38 | (+)-Camphor | [M+H]+ | 153.12739 | 153.12729 | -0.7 | C10H16O |
| 39 | (+)-Borneol | [M+H]+ | 155.14304 | 155.14315 | 0.7 | C10H18O |
| 40 | Pyrazinamide | [M+Cl]- | 158.01266 | 158.01251 | -1.0 | C5H5N3O |
| 41 | D-Alanyl-D-alanine | [M-H]- | 159.07752 | 159.07740 | -0.7 | C6H12N2O3 |
| 42 | 7-Mercaptoheptanoic acid | [M-H]- | 161.06417 | 161.06378 | -2.4 | C7H14O2S |
| 43 | 3 Hydroxycoumarin | [M+H]+ | 163.03897 | 163.03888 | -0.6 | C9H6O3 |
| 44 | 2-Methyl-6-oxohepta-2,4-dienal | [M+Na]+ | 163.07295 | 163.07315 | 1.2 | C8H12O2 |
| 45 | (-)-Anabasine | [M+H]+ | 163.12297 | 163.12305 | 0.5 | C10H14N2 |
| 46 | (R)-2-Methylimino-1-phenylpropan-1-ol | [M+H]+ | 164.10699 | 164.10721 | 1.3 | C10H13NO |
| 47 | 1-Hexenyl acetate | [M+Na]+ | 165.08860 | 165.08853 | -0.4 | C8H14O2 |
| 48 | 2,4-decadiynoic acid | [M+H]+ | 165.09101 | 165.09145 | 2.7 | C10H12O2 |

| 49 | (+)-Bornane-2,5-dione | [M-H]- | 165.09210 | 165.09247 | 2.2 | C10H14O2 |
| --- | --- | --- | --- | --- | --- | --- |
| 50 | 4-Trimethylammoniobutanal | [M+Cl]- | 165.09259 | 165.09247 | -0.7 | C7H16NO |
| 51 | Fenuron | [M+H]+ | 165.10224 | 165.10250 | 1.6 | C9H12N2O |
| 52 | Proline betaine | [M+Na]+ | 166.08385 | 166.08347 | -2.3 | C7H13NO2 |
| 53 | 3-Formylsalicylic acid | [M+H]+ | 167.03389 | 167.03390 | 0.1 | C8H6O4 |
| 54 | Cyromazine | [M+H]+ | 167.10397 | 167.10427 | 1.8 | C6H10N6 |
| 55 | 2,2-dimethyl-hexanoic acid | [M+Na]+ | 167.10425 | 167.10427 | 0.1 | C8H16O2 |
| 56 | (+)-Iridodial | [M+H]+ | 169.12231 | 169.12230 | 0.0 | C10H16O2 |
| 57 | Furfural diethyl acetal | [M+H]+ | 171.10157 | 171.10181 | 1.4 | C9H14O3 |
| 58 | 2,6-nonadienoic acid | [M+Na]+ | 177.08860 | 177.08900 | 2.3 | C9H14O2 |
| 59 | (+)-Borneol | [M+Na]+ | 177.12499 | 177.12457 | -2.3 | C10H18O |
| 60 | L-Carbocisteine | [M-H]- | 178.01795 | 178.01787 | -0.5 | C5H9NO4S |
| 61 | Adrenochrome o-semiquinone | [M-H]- | 179.05879 | 179.05857 | -1.2 | C9H10NO3 |
| 62 | 4-Hydroxy-enol-phenylpyruvate | [M+H]+ | 181.04954 | 181.04970 | 0.9 | C9H8O4 |
| 63 | 1-Ethyl-2-benzimidazolinone | [M+Na]+ | 185.06853 | 185.06811 | -2.3 | C9H10N2O |
| 64 | Propanoylagmatine | [M+H]+ | 187.15534 | 187.15490 | -2.3 | C8H18N4O |
| 65 | 5-Methoxytryptophol | [M-H]- | 190.08735 | 190.08740 | 0.3 | C11H13NO2 |
| 66 | 6,8-Dihydroxypurine | [M+K]+ | 190.99658 | 190.99632 | -1.4 | C5H4N4O2 |
| 67 | 2,4-Dihydroxyheptenedioate | [M+H]+ | 191.05501 | 191.05466 | -1.9 | C7H10O6 |
| 68 | Octopamine | [M+K]+ | 192.04214 | 192.04251 | 1.9 | C8H11NO2 |
| 69 | N-Butyl-pyrazolopyrimidin-4-amine | [M+H]+ | 192.12437 | 192.12406 | -1.6 | C9H13N5 |
| 70 | Linalool oxide | [M+Na]+ | 193.11990 | 193.11967 | -1.2 | C10H18O2 |
| 71 | 3-caproyl propionic acid | [M+Na]+ | 195.09917 | 195.09963 | 2.4 | C9H16O3 |
| 72 | Phosphoguanidinoacetate | [M-H]- | 196.01288 | 196.01246 | -2.1 | C3H8N3O5P |
| 73 | Coryneine | [M+H]+ | 197.14103 | 197.14132 | 1.5 | C11H18NO2 |
| 74 | 1-Hexylglycerol | [M+Na]+ | 199.13047 | 199.13010 | -1.8 | C9H20O3 |
| 75 | Trimethyl-L-histidine | [M+H]+ | 199.13153 | 199.13179 | 1.3 | C9H16N3O2 |
| 76 | 2-methyl-undecanoic acid | [M-H]- | 199.17035 | 199.17059 | 1.2 | C12H24O2 |
| 77 | Guanethidine | [M+H]+ | 199.19172 | 199.19164 | -0.4 | C10H22N4 |
| 78 | Cyclohexylsulfamate | [M+Na]+ | 202.05084 | 202.05064 | -1.0 | C6H13NO3S |
| 79 | 2-Deoxy-D-gluconate | [M+Na]+ | 203.05261 | 203.05222 | -1.9 | C6H12O6 |
| 80 | 5-L-Glutamylglycine | [M+H]+ | 205.08190 | 205.08187 | -0.1 | C7H12N2O5 |
| 81 | Naphthalene-1-sulfonate | [M-H]- | 207.01214 | 207.01208 | -0.3 | C10H8O3S |
| 82 | (3R)-3-Isopropenyl-6-oxoheptanoate | [M+Na]+ | 207.09917 | 207.09900 | -0.8 | C10H16O3 |
| 83 | Echinorine | [M+Cl]- | 209.06129 | 209.06151 | 1.0 | C11H12NO |
| 84 | Formyl-5-hydroxykynurenamine | [M+H]+ | 209.09207 | 209.09181 | -1.2 | C10H12N2O3 |
| 85 | (3R)-6-Hydroxy-3-isopropenyl-heptanoate | [M+Na]+ | 209.11482 | 209.11480 | -0.1 | C10H18O3 |
| 86 | (R)-3-Hydroxydecanoic acid | [M+Na]+ | 211.13047 | 211.13031 | -0.7 | C10H20O3 |
| 87 | N,N-Diethyl-m-toluamide | [M+Na]+ | 214.12023 | 214.12087 | 3.0 | C12H17NO |
| 88 | N-Acetyl-L-arginine | [M+H]+ | 217.12952 | 217.12958 | 0.3 | C8H16N4O3 |
| 89 | 1-Hydroxypyrene | [M+H]+ | 219.08044 | 219.08057 | 0.6 | C16H10O |
| 90 | (+)-2-Sterpurene-6-ol | [M-H]- | 219.17544 | 219.17545 | 0.1 | C15H24O |
| 91 | 2E-Decenedioic acid | [M+Na]+ | 223.09408 | 223.09373 | -1.6 | C10H16O4 |
| 92 | (+/-)-6-Hydroxy-3-oxo-α-ionone | [M+H]+ | 223.13287 | 223.13290 | 0.1 | C13H18O3 |
| 93 | 12-hydroxyjasmonic acid | [M+H]+ | 227.12779 | 227.12803 | 1.1 | C12H18O4 |
| 94 | Myristic acid | [M-H]- | 227.20165 | 227.20133 | -1.4 | C14H28O2 |
| 95 | Deoxyuridine | [M+H]+ | 229.08190 | 229.08145 | -2.0 | C9H12N2O5 |
| 96 | N-Acetyl-L-2-amino-6-oxopimelate | [M-H]- | 230.06701 | 230.06663 | -1.7 | C9H13NO6 |
| 97 | 4-(Glutamylamino) butanoate | [M+H]+ | 233.11320 | 233.11268 | -2.2 | C9H16N2O5 |
| 98 | 2,6-Diamino-7-hydroxy-azelaic acid | [M+H]+ | 235.12885 | 235.12929 | 1.9 | C9H18N2O5 |

| 99 | (+)-7-epi-9,10-dihydrojasmonic acid | [M+Na]+ | 235.13047 | 235.13044 | -0.1 | C12H20O3 |
| --- | --- | --- | --- | --- | --- | --- |
| 100 | 10-hydroxy-11-dodecenoic acid | [M+Na]+ | 237.14612 | 237.14631 | 0.8 | C12H22O3 |
| 101 | Succinyl proline | [M+Na]+ | 238.06859 | 238.06900 | 1.7 | C9H13NO5 |
| 102 | Undecanedioic acid | [M+Na]+ | 239.12538 | 239.12467 | -3.0 | C11H20O4 |
| 103 | 10Z-Tridecenyl acetate | [M-H]- | 239.20165 | 239.20158 | -0.3 | C15H28O2 |
| 104 | α-Amylcinnamaldehyde | [M+K]+ | 241.09892 | 241.09822 | -2.9 | C14H18O |
| 105 | (+)-12-methyl myristic acid | [M-H]- | 241.21730 | 241.21740 | 0.4 | C15H30O2 |
| 106 | (+)-Mayurone | [M+K]+ | 245.13022 | 245.13090 | 2.8 | C14H22O |
| 107 | Aspergillic acid | [M+Na]+ | 247.14170 | 247.14172 | 0.1 | C12H20N2O2 |
| 108 | 2-Amino-3-oxo-4-phosphonooxybutyrate | [M+Cl]- | 247.97324 | 247.97341 | 0.7 | C4H8NO7P |
| 109 | 2-[2-(4-Pyridinyl)-1-butenyl]phenol | [M+Na]+ | 248.10458 | 248.10423 | -1.4 | C15H15NO |
| 110 | 1,8-Diazacyclotetradecane-2,9-dione | [M+Na]+ | 249.15735 | 249.15776 | 1.7 | C12H22N2O2 |
| 111 | (-)-11-hydroxy-9,10-dihydrojasmonic acid | [M+Na]+ | 251.12538 | 251.12470 | -2.7 | C12H20O4 |
| 112 | Palmitoleic acid | [M-H]- | 253.21730 | 253.21655 | -3.0 | C16H30O2 |
| 113 | N-ribosylnicotinamide | [M-H]- | 254.09082 | 254.09093 | 0.4 | C11H15N2O5 |
| 114 | Darlingine | [M+Cl]- | 254.09533 | 254.09536 | 0.1 | C13H17NO2 |
| 115 | Palmitic Acid | [M-H]- | 255.23295 | 255.23225 | -2.8 | C16H32O2 |
| 116 | Dodecatrienyl acetate | [M+K]+ | 261.12514 | 261.12516 | 0.1 | C14H22O2 |
| 117 | 10-methyl-9-hexadecenoic acid | [M-H]- | 267.23295 | 267.23322 | 1.0 | C17H32O2 |
| 118 | Deoxycoformycin | [M+H]+ | 269.12443 | 269.12401 | -1.6 | C11H16N4O4 |
| 119 | 17α-Dihydroequilenin | [M+H]+ | 269.15361 | 269.15296 | -2.4 | C18H20O2 |
| 120 | (+)-14-methyl palmitic acid | [M-H]- | 269.24860 | 269.24839 | -0.8 | C17H34O2 |
| 121 | Anatalline | [M+Cl]- | 274.11165 | 274.11101 | -2.3 | C15H17N3 |
| 122 | 1-(3,4-Dihydroxyphenyl)-1-decene-3,5-dione | [M+H]+ | 277.14344 | 277.14281 | -2.3 | C16H20O4 |
| 123 | 2-Ethylhexyl phthalate | [M-H]- | 277.14453 | 277.14440 | -0.5 | C16H22O4 |
| 124 | Metalaxyl | [M-H]- | 278.13978 | 278.13982 | 0.1 | C15H21NO4 |
| 125 | Methyl nigakinone | [M-H]- | 279.07752 | 279.07714 | -1.3 | C16H12N2O3 |
| 126 | 2-N-Undecyltetrahydrothiophene | [M+K]+ | 281.16998 | 281.16998 | 0.0 | C15H30S |
| 127 | (11E)-Octadecenoic acid | [M-H]- | 281.24860 | 281.24847 | -0.5 | C18H34O2 |
| 128 | (+)-Isostearic acid | [M-H]- | 283.26425 | 283.26500 | 2.6 | C18H36O2 |
| 129 | Embelin | [M-H]- | 293.17583 | 293.17641 | 2.0 | C17H26O4 |
| 130 | Doisynoestrol | [M-H]- | 297.14962 | 297.14974 | 0.4 | C19H22O3 |
| 131 | (-)-Sparticarpin | [M-H]- | 299.09250 | 299.09176 | -2.5 | C17H16O5 |
| 132 | (S)-3'-Hydroxycoclaurine | [M+H]+ | 302.13868 | 302.13917 | 1.6 | C17H19NO4 |
| 133 | Arachidonoyl amine | [M-H]- | 302.24894 | 302.24844 | -1.6 | C20H33NO |
| 134 | 11-Octadecen-1-ol | [M+Cl]- | 303.24602 | 303.24632 | 1.0 | C18H36O |
| 135 | Phenylbutazone | [M+H]+ | 309.15975 | 309.15954 | -0.7 | C19H20N2O2 |
| 136 | 5-O-Methylembelin | [M+H]+ | 309.20604 | 309.20601 | -0.1 | C18H28O4 |
| 137 | L-Tyrosine methyl ester 4-sulfate | [M+Cl]- | 310.01576 | 310.01659 | 2.7 | C10H13NO6S |
| 138 | 2-Ethylidene-1,5-dimethyl-3,3-diphenylpyrrolidine | [M+Cl]- | 312.15245 | 312.15254 | 0.3 | C20H23N |
| 139 | 5-(5-(4-(4,5-Dihydro-2-oxazoly)phenoxy)pentyl)-3-methylisoxazole | [M-H]- | 313.15577 | 313.15605 | 0.9 | C18H22N2O3 |
| 140 | (2S)-4'-Hydroxy-5,7,3'-trimethoxyflavan | [M-H]- | 315.12380 | 315.12366 | -0.4 | C18H20O5 |
| 141 | Adenocarpine | [M+Na]+ | 319.17808 | 319.17890 | 2.6 | C19H24N2O |
| 142 | 1-Methylestradiol | [M+Cl]- | 321.16268 | 321.16184 | -2.6 | C19H26O2 |
| 143 | Fluoroandrost-en-ol | [M+Cl]- | 327.18965 | 327.18969 | 0.1 | C19H29FO |
| 144 | 11,12,15-trihydroxy palmitic acid | [M+Cl]- | 339.19438 | 339.19401 | -1.1 | C16H32O5 |
| 145 | Clavepictine B | [M+Cl]- | 340.24127 | 340.24037 | -2.6 | C20H35NO |
| 146 | tricosahexaenoic acid | [M-H]- | 341.24860 | 341.24872 | 0.3 | C23H34O2 |
| 147 | 8-Epiiridotrial glucoside | [M-H]- | 343.13984 | 343.13894 | -2.6 | C16H24O8 |

| 148 | Fulvine | [M+Cl]- | 344.12702 | 344.12706 | 0.1 | C16H23NO5 |
| --- | --- | --- | --- | --- | --- | --- |
| 149 | Callytriol C | [M+H]+ | 349.17982 | 349.17935 | -1.3 | C23H24O3 |
| 150 | N-(Heptadecanoyl)-ethanolamine | [M+K]+ | 352.26124 | 352.26175 | 1.5 | C19H39NO2 |
| 151 | 5-hydroperoxy-7-[3,5-epidioxy-2-(2-octenyl)-cyclopentyl]-6-  heptenoic acid | [M-H]- | 353.19696 | 353.19730 | 1.0 | C19H30O6 |
| 152 | Docosapentaynoic acid | [M+Cl]- | 355.14703 | 355.14664 | -1.1 | C22H24O2 |
| 153 | 3-hydroxy-docosanoic acid | [M-H]- | 355.32177 | 355.32156 | -0.6 | C22H44O3 |
| 154 | 19-oic-deoxycorticosterone | [M-H]- | 358.17857 | 358.17825 | -0.9 | C21H27O5 |
| 155 | Lycofawcine | [M+Cl]- | 358.17906 | 358.17825 | -2.3 | C18H29NO4 |
| 156 | 1-Methylethyl glucosinolate | [M-H]- | 360.04285 | 360.04299 | 0.4 | C10H19NO9S2 |
| 157 | Gibberellin A24 | [M+Cl]- | 381.14743 | 381.14820 | 2.0 | C20H26O5 |
| 158 | S-Adenosylmethioninamine | [M+Cl]- | 390.12464 | 390.12414 | -1.3 | C14H23N6O3S |
| 159 | 10-epi-Eupatoroxin | [M-H]- | 391.13984 | 391.13925 | -1.5 | C20H24O8 |
| 160 | N-stearoyl serine | [M+Na]+ | 394.29278 | 394.29178 | -2.5 | C21H41NO4 |
| 161 | (+)-Dysideapalaunic acid | [M+Na]+ | 395.29205 | 395.29228 | 0.6 | C25H40O2 |
| 162 | Thyrotropin releasing hormone | [M+Cl]- | 397.13965 | 397.13998 | 0.8 | C16H22N6O4 |
| 163 | Isopentenyladenine-9-N-glucoside | [M+Cl]- | 398.16006 | 398.15944 | -1.5 | C17H25N5O4 |
| 164 | (Ac)2-L-Lys-D-Ala-D-Ala | [M+Cl]- | 407.17029 | 407.16956 | -1.8 | C16H28N4O6 |
| 165 | (+)-24-methyl-hexacosanoic acid | [M-H]- | 409.40510 | 409.40623 | 2.7 | C27H54O2 |
| 166 | 3,5-Dihydroxy-6,7,8-trimethoxy-3',4'-methylenedioxyflavone | [M+Na]+ | 411.06865 | 411.06859 | -0.2 | C19H16O9 |
| 167 | Geniposide | [M+Na]+ | 411.12617 | 411.12651 | 0.8 | C17H24O10 |
| 168 | N5-Dinitrophenyl-L-ornithine methyl ester | [M+Na]+ | 411.12751 | 411.12651 | -2.4 | C18H20N4O6 |
| 169 | Hydroxy-3-oxo-cholenoic Acid | [M+Na]+ | 411.25058 | 411.25023 | -0.9 | C24H36O4 |
| 170 | 1-Palmitoylglycerol 3-phosphate | [M+H]+ | 411.25062 | 411.25023 | -0.9 | C19H39O7P |
| 171 | Gonyautoxin 1 | [M+H]+ | 412.08812 | 412.08756 | -1.4 | C10H17N7O9S |
| 172 | (amino-hydroxy-oxopropyl)sulfanyl-dihydroxyicosatetraenoic acid | [M+H]+ | 412.14245 | 412.14296 | 1.2 | C19H25NO7S |
| 173 | Androsta-5,16-dieno-quinolin-ol | [M+K]+ | 412.20372 | 412.20300 | -1.8 | C26H31NO |
| 174 | Acetophenazine | [M+H]+ | 412.20532 | 412.20480 | -1.3 | C23H29N3O2S |
| 175 | Curacin A | [M+K]+ | 412.20710 | 412.20758 | 1.2 | C23H35NOS |
| 176 | Sphingofungin B | [M+Na]+ | 412.26696 | 412.26762 | 1.6 | C20H39NO6 |
| 177 | 2'-Carboxy-[bis(chloroethyl)amino]-2-methylazobenzene | [M+Cl]- | 414.05483 | 414.05470 | -0.3 | C18H19Cl2N3O2 |
| 178 | S-Hexyl-glutathione | [M+Na]+ | 414.16693 | 414.16784 | 2.2 | C16H29N3O6S |
| 179 | Flavoxate | [M+Na]+ | 414.16758 | 414.16784 | 0.6 | C24H25NO4 |
| 180 | N-arachidonoyl D-serine | [M+Na]+ | 414.26148 | 414.26207 | 1.4 | C23H37NO4 |
| 181 | N-stearoyl taurine | [M+Na]+ | 414.26485 | 414.26401 | -2.0 | C20H41NO4S |
| 182 | 7-Epiloganic acid | [M+K]+ | 415.10011 | 415.10024 | 0.3 | C16H24O10 |
| 183 | 3'-Deoxyderhamnosylmaysin | [M+H]+ | 415.10236 | 415.10200 | -0.9 | C21H18O9 |
| 184 | Ovaliflavanone A | [M+K]+ | 415.16700 | 415.16653 | -1.1 | C25H28O3 |
| 185 | 1-Methyl-2-hydroxyetyl glucosinolate | [M+K]+ | 416.00820 | 416.00784 | -0.9 | C10H19NO10S2 |
| 186 | C19 Sphingosine-1-phosphate | [M+Na]+ | 416.25363 | 416.25355 | -0.2 | C19H40NO5P |
| 187 | [Bis(2-hydroxyethyl)amino]androsten-ol | [M+K]+ | 416.25615 | 416.25558 | -1.4 | C23H39NO3 |
| 188 | Picrasin C | [M-H]- | 421.22318 | 421.22434 | 2.8 | C23H34O7 |
| 189 | DG(18:0e/2:0/0:0) | [M+Cl]- | 421.30901 | 421.30806 | -2.3 | C23H46O4 |
| 190 | Hydroxymethylcholesta-dienol | [M+Na]+ | 436.33118 | 436.33245 | 2.9 | C28H45O2 |
| 191 | N'-5Z,8Z,11Z,14Z-eicosatetraenoyl-N''-diethyl-ethylenediamine | [M+Cl]- | 437.33042 | 437.33128 | 2.0 | C26H46N2O |
| 192 | 5,7,2',6'-Tetrahydroxyflavone 2'-O-glucoside | [M-H]- | 447.09328 | 447.09215 | -2.5 | C21H20O11 |
| 193 | Eupacunolin | [M+Cl]- | 455.14782 | 455.14717 | -1.4 | C22H28O8 |
| 194 | 5-Hydroxypseudobaptigenin 7-O-glucoside | [M-H]- | 459.09328 | 459.09454 | 2.7 | C22H20O11 |

| 195 | (R)-nonacosan-10-ol | [M+Cl]- | 459.43382 | 459.43517 | 2.9 | C29H60O |
| --- | --- | --- | --- | --- | --- | --- |
| 196 | Trihydroxy-oxo-cholanoic Acid | [M+K]+ | 461.23000 | 461.22884 | -2.5 | C24H38O6 |
| 197 | PA(8:0/8:0) | [M+K]+ | 463.18576 | 463.18626 | 1.1 | C19H37O8P |
| 198 | (+)-Plicamine | [M+H]+ | 463.18636 | 463.18626 | -0.2 | C26H26N2O6 |
| 199 | Trihydroxy-methyl-cholestenoic acid | [M+H]+ | 463.34180 | 463.34297 | 2.5 | C28H46O5 |
| 200 | (+)-22(29)-Hopen-6α-21β-diol | [M+Na]+ | 465.37030 | 465.37036 | 0.1 | C30H50O2 |
| 201 | Mycolipanolic acid (C27) | [M+K]+ | 465.37046 | 465.37036 | -0.2 | C27H54O3 |
| 202 | Artocommunol CA | [M+Cl]- | 467.12669 | 467.12621 | -1.0 | C26H24O6 |
| 203 | N-Acetyl-leukotriene E4 | [M-H]- | 480.24253 | 480.24137 | -2.4 | C25H39NO6S |
| 204 | Deoxytubulosine | [M+Na]+ | 482.27780 | 482.27737 | -0.9 | C29H37N3O2 |
| 205 | Serratanine | [M+Na]+ | 490.37678 | 490.37771 | 1.9 | C30H49N3O |
| 206 | N3'-Acetylgentamicin | [M+H]+ | 497.24533 | 497.24415 | -2.4 | C19H36N4O11 |
| 207 | 3'-Keto-3'-deoxy-ATP | [M-H]- | 503.97282 | 503.97313 | 0.6 | C10H14N5O13P3 |
| 208 | N-tert-Butyloxycarbonyl-deacetyl-leupeptin | [M+Na]+ | 507.32654 | 507.32734 | 1.6 | C23H44N6O5 |
| 209 | Hydrocortisone cypionate | [M+K]+ | 525.26130 | 525.26276 | 2.8 | C29H42O6 |
| 210 | LysoPC(20:5) | [M-H]- | 540.30956 | 540.30797 | -2.9 | C28H48NO7P |
| 211 | 11α-Hemiglutaryloxy-1,25-dihydroxyvitamin D3 | [M+H]+ | 545.38367 | 545.38260 | -2.0 | C33H52O6 |
| 212 | Antimycin A1 | [M+H]+ | 549.28066 | 549.28096 | 0.6 | C28H40N2O9 |
| 213 | Isorenieratene | [M+Na]+ | 551.36482 | 551.36484 | 0.0 | C40H48 |
| 214 | 2-(8-[3]-ladderane-octanyl)-sn-glycero-3-phosphocholine | [M+Na]+ | 552.34245 | 552.34363 | 2.1 | C28H52NO6P |
| 215 | 1-O-(1Z-Tetradecenyl)-2-(9Z-octadecenoyl)-sn-glycerol | [M+Cl]- | 585.46551 | 585.46494 | -1.0 | C35H66O4 |
| 216 | 12-Dihydrodalbinol O-glucoside | [M-H]- | 589.19266 | 589.19241 | -0.4 | C29H34O13 |
| 217 | L-Urobilinogen | [M+H]+ | 597.36466 | 597.36361 | -1.8 | C33H48N4O6 |
| 218 | Cyanidin 3-lathyroside | [M+Cl]- | 616.12005 | 616.11827 | -2.9 | C26H29O15 |
| 219 | PA(12:0/17:2) | [M+Na]+ | 625.38398 | 625.38402 | 0.1 | C32H59O8P |
| 220 | Hederagenin 3-O-arabinoside | [M+Na]+ | 627.38674 | 627.38507 | -2.7 | C35H56O8 |
| 221 | PA(12:0/18:3) | [M+Cl]- | 649.36416 | 649.36446 | 0.5 | C33H59O8P |
| 222 | 3-((3-Cholamidopropyl)dimethylammonium)-1-propanesulfonate | [M+Cl]- | 649.36587 | 649.36446 | -2.2 | C32H58N2O7S |
| 223 | 2S,3R-Didecanoyl-docosane-2,3-diol | [M-H]- | 649.61403 | 649.61469 | 1.0 | C42H82O4 |
| 224 | 12-O-Tetradecanoylphorbol 13-acetate | [M+K]+ | 655.36068 | 655.36054 | -0.2 | C36H56O8 |
| 225 | Geissospermine | [M+Na]+ | 655.36186 | 655.36054 | -2.0 | C40H48N4O3 |
| 226 | 6,8-Di-C-glucopyranosyltricetin | [M+Cl]- | 661.11770 | 661.11671 | -1.5 | C27H30O17 |
| 227 | 20:5 Cholesteryl ester | [M-H]- | 669.56161 | 669.56246 | 1.3 | C47H74O2 |
| 228 | PE(14:1/P-18:1) | [M+H]+ | 672.49627 | 672.49620 | -0.1 | C37H70NO7P |
| 229 | PA(O-16:0/19:1) | [M-H]- | 673.51776 | 673.51685 | -1.4 | C38H75O7P |
| 230 | TG(12:0/12:0/12:0) | [M+Cl]- | 673.51794 | 673.51685 | -1.6 | C39H74O6 |
| 231 | 2-Octaprenylphenol | [M+K]+ | 677.50583 | 677.50538 | -0.7 | C46H70O |
| 232 | Kaempferol 3-(6G-malonylneohesperidoside) | [M-H]- | 679.15159 | 679.15209 | 0.7 | C30H32O18 |
| 233 | PE(12:0/20:4) | [M+H]+ | 684.45988 | 684.46025 | 0.5 | C37H66NO8P |
| 234 | Cer(d18:0/24:0) | [M+Cl]- | 686.62235 | 686.62370 | 2.0 | C42H85NO3 |
| 235 | PA(13:0/22:0) | [M-H]- | 689.51268 | 689.51228 | -0.6 | C38H75O8P |
| 236 | Gabunamine | [M+H]+ | 691.38540 | 691.38388 | -2.2 | C42H50N4O5 |
| 237 | PG(O-16:0/14:1) | [M+Na]+ | 701.47279 | 701.47234 | -0.6 | C36H71O9P |
| 238 | Fusicoccin A | [M+Na]+ | 705.38205 | 705.38183 | -0.3 | C36H58O12 |
| 239 | PI(13:0/12:0) | [M+H]+ | 713.42356 | 713.42520 | 2.3 | C34H65O13P |
| 240 | PG(12:0/18:0) | [M+Na]+ | 717.46771 | 717.46746 | -0.3 | C36H71O10P |
| 241 | PA(O-16:0/20:4) | [M+K]+ | 721.45690 | 721.45883 | 2.7 | C39H71O7P |
| 242 | PS(12:0/19:0) | [M+H]+ | 722.49666 | 722.49480 | -2.6 | C37H72NO10P |
| 243 | PI(12:0/14:0) | [M+H]+ | 727.43921 | 727.44040 | 1.6 | C35H67O13P |

| 244 | SM(d18:0/14:1(OH)) | [M+K]+ | 727.47870 | 727.47766 | -1.4 | C37H73N2O7P |
| --- | --- | --- | --- | --- | --- | --- |
| 245 | PA(O-20:0/20:3) | [M+Na]+ | 763.56121 | 763.56260 | 1.8 | C43H81O7P |
| 246 | PA(O-20:0/22:6) | [M+H]+ | 763.56362 | 763.56260 | -1.3 | C45H79O7P |
| 247 | DG(20:5/24:1/0:0) | [M+K]+ | 763.56374 | 763.56260 | -1.5 | C47H80O5 |
| 248 | PS(12:0/22:0) | [M+H]+ | 764.54361 | 764.54398 | 0.5 | C40H78NO10P |
| 249 | Galactosylceramide (d18:1/18:1) | [M+K]+ | 764.54373 | 764.54398 | 0.3 | C42H79NO8 |
| 250 | PC(12:0/22:6) | [M+Na]+ | 772.48878 | 772.48977 | 1.3 | C42H72NO8P |
| 251 | PS(O-16:0/17:1) | [M+K]+ | 772.48893 | 772.48977 | 1.1 | C39H76NO9P |
| 252 | Coenzyme F420 | [M+H]+ | 774.18657 | 774.18428 | -3.0 | C29H36N5O18P |
| 253 | PI(P-16:0/15:1) | [M+H]+ | 779.50689 | 779.50793 | 1.3 | C40H75O12P |
| 254 | Acanthoside D | [M+K]+ | 781.23157 | 781.23045 | -1.4 | C34H46O18 |
| 255 | PG(13:0/22:2) | [M+Na]+ | 783.51466 | 783.51378 | -1.1 | C41H77O10P |
| 256 | PI(O-16:0/15:0) | [M+H]+ | 783.53819 | 783.53928 | 1.4 | C40H79O12P |
| 257 | PG(15:0/22:4) | [M+H]+ | 785.53271 | 785.53309 | 0.5 | C43H77O10P |
| 258 | PA(O-18:0/22:0) | [M+K]+ | 785.58210 | 785.58128 | -1.0 | C43H87O7P |
| 259 | Fumonisin A1 | [M+Na]+ | 786.38826 | 786.38869 | 0.6 | C36H61NO16 |
| 260 | DGTA(18:1/22:4) | [M+H]+ | 786.62423 | 786.62435 | 0.2 | C48H83NO7 |
| 261 | PC(O-16:0/19:1) | [M+K]+ | 798.57735 | 798.57879 | 1.8 | C43H86NO7P |
| 262 | Formylmethanofuran | [M+Na]+ | 799.26445 | 799.26271 | -2.2 | C35H44N4O16 |
| 263 | PC(15:0/22:2) | [M+H]+ | 800.61638 | 800.61587 | -0.6 | C45H86NO8P |
| 264 | PC(13:0/22:6) | [M+K]+ | 802.47836 | 802.47962 | 1.6 | C43H74NO8P |
| 265 | PC(20:0/P-18:0) | [M+H]+ | 802.66842 | 802.67017 | 2.2 | C46H92NO7P |
| 266 | PA(20:5/22:6) | [M+K]+ | 805.42052 | 805.41979 | -0.9 | C45H67O8P |
| 267 | PS(14:0/22:4) | [M+Na]+ | 806.49425 | 806.49409 | -0.2 | C42H74NO10P |
| 268 | Isoswertisin 6'''-O-feruloyl 2''-O-glucoside | [M+Na]+ | 807.21069 | 807.21035 | -0.4 | C38H40O18 |
| 269 | PA(O-20:0/22:1) | [M+K]+ | 811.59775 | 811.59681 | -1.2 | C45H89O7P |
| 270 | PI(17:0/14:1) | [M+H]+ | 812.52835 | 812.52761 | -0.9 | C40H78NO13P |
| 271 | Bayogenin 3-O-cellobioside | [M+H]+ | 813.46310 | 813.46103 | -2.5 | C42H68O15 |
| 272 | TG(16:1/16:1/17:2) | [M+H]+ | 813.69667 | 813.69570 | -1.2 | C52H92O6 |
| 273 | PI(13:0/18:2) | [M+Na]+ | 815.46810 | 815.46899 | 1.1 | C40H73O13P |
| 274 | PI(13:0/20:5) | [M+H]+ | 815.47051 | 815.46899 | -1.9 | C42H71O13P |
| 275 | Oligomycin D | [M+K]+ | 815.47062 | 815.46899 | -2.0 | C44H72O11 |
| 276 | SQDG(16:0/16:0) | [M+Na]+ | 816.50279 | 816.50283 | 0.0 | C41H77O12S |
| 277 | PS(O-20:0/19:1) | [M+H]+ | 818.62695 | 818.62522 | -2.1 | C45H88NO9P |
| 278 | Digoxin | [M+K]+ | 819.39277 | 819.39185 | -1.1 | C41H64O14 |
| 279 | PG(15:1/22:4) | [M+K]+ | 821.47294 | 821.47464 | 2.1 | C43H75O10P |
| 280 | Leucomycin A6 | [M+Na]+ | 822.42464 | 822.42555 | 1.1 | C40H65NO15 |
| 281 | TG(16:0/14:0/18:1) | [M+Na]+ | 827.70991 | 827.70752 | -2.9 | C51H96O6 |
| 282 | PC(20:3/P-18:1) | [M+K]+ | 832.56170 | 832.56072 | -1.2 | C46H84NO7P |
| 283 | UDP-3-O-(3-hydroxytetradecanoyl)-N-acetylglucosamine | [M+H]+ | 834.28213 | 834.27989 | -2.7 | C31H53N3O19P2 |
| 284 | PS(16:0/22:4) | [M+Na]+ | 834.52556 | 834.52450 | -1.3 | C44H78NO10P |
| 285 | TG(17:1/20:5/20:5) | [M+Na]+ | 933.69426 | 933.69414 | -0.1 | C60H94O6 |
| 286 | Kaempferol 3-(2'''-(E)-p-coumarylsophoroside)-7-glucoside | [M+K]+ | 957.20615 | 957.20785 | 1.8 | C42H46O23 |
| a Cer: Ceramide; GalCer: Galactosylceramide; GlcCer: Glucosylceramide; ; LacCer: Lactosylceramide; MG: Monoacylglycerol; DG: Diacylglycerol; TG: Triacylglycerol; MGDG: Monoacyldiacylglycerol; PA: Phosphatidic acid; PC: Phosphatidylcholine; PE: Phosphatidylethanolamine; PG(P): Glycerophospholipids; PI: Phosphatidylinositol; PS: Phosphatidylserine; SM: Sphingomyelin; CDP: Cytidine diphosphate; UDP: Uridine diphospate; SQMG: sulfoquinovosylmonoacylglycerols | | | | | | |
