# Supplementary material for: Satureja montana L. Essential Oils: Chemical Profiles/Phytochemical Screening, Antimicrobial Activity and O/W NanoEmulsion Formulations
Source: Pharmaceutics. 2019 Dec 19;12(1):7. doi: 10.3390/pharmaceutics12010007 (PMC7022231; doi:10.3390/pharmaceutics12010007)
Supplement: Supplementary file 1 [file pharmaceutics-12-00007-s001.zip › pharmaceutics-623925-SI-final/TableS3.docx]

| Table S3: SEO3 Untargeted ESI FT-ICR Annotations | | | | | | |
| --- | --- | --- | --- | --- | --- | --- |
| No. | Compound (M)^a^ | Ion | Theor. m/z | Exp. m/z | Δppm | Formula |
| 1 | D-Lactic acid | [M-H]- | 89.02442 | 89.02437 | -0.6 | C3H6O3 |
| 2 | Hexenol | [M-H]- | 99.08154 | 99.08165 | 1.1 | C6H12O |
| 3 | Choline | [M]+ | 104.10699 | 104.10713 | 1.3 | C5H14NO |
| 4 | Ethylbenzene | [M+H]+ | 107.08553 | 107.08554 | 0.1 | C8H10 |
| 5 | 3-Cresol | [M+H]+ | 109.06479 | 109.06493 | 1.3 | C7H8O |
| 6 | (S)-1-Phenylethanol | [M+H]+ | 123.08044 | 123.08030 | -1.2 | C8H10O |
| 7 | 2-Methyl-3-buten-1-ol | [M+K]+ | 125.03632 | 125.03624 | -0.7 | C5H10O |
| 8 | 1-Pyrroline-4-hydroxy-2-carboxylate | [M-H]- | 128.03532 | 128.03529 | -0.2 | C5H7NO3 |
| 9 | Diethylene glycol | [M+Na]+ | 129.05221 | 129.05248 | 2.1 | C4H10O3 |
| 10 | Octylamine | [M+H]+ | 130.15903 | 130.15873 | -2.3 | C8H19N |
| 11 | Orthophosphate | [M+Cl]- | 132.94630 | 132.94597 | -2.5 | H3PO4 |
| 12 | 2-Hydroxypyridine | [M+K]+ | 134.00027 | 133.99999 | -2.1 | C5H5NO |
| 13 | Cymene | [M+H]+ | 135.11683 | 135.11673 | -0.7 | C10H14 |
| 14 | ε-Caprolactam | [M+Na]+ | 136.07328 | 136.07357 | 2.1 | C6H11NO |
| 15 | Terpinene | [M+H]+ | 137.13248 | 137.13253 | 0.4 | C10H16 |
| 16 | Sulfoacetate | [M-H]- | 138.97067 | 138.97043 | -1.7 | C2H4O5S |
| 17 | 1-Hexen-1-ol | [M+K]+ | 139.05197 | 139.05227 | 2.2 | C6H12O |
| 18 | (2E,6Z)-nona-2,6-dienal | [M+H]+ | 139.11174 | 139.11213 | 2.8 | C9H14O |
| 19 | Cadaverine | [M+K]+ | 141.07886 | 141.07872 | -1.0 | C5H14N2 |
| 20 | 3-Fluoro-D-alanine | [M+Cl]- | 142.00766 | 142.00761 | -0.3 | C3H6FNO2 |
| 21 | 1,2-Diaminobenzene | [M+Cl]- | 143.03815 | 143.03801 | -1.0 | C6H8N2 |
| 22 | 2,2-dimethyl-hexanoic acid | [M-H]- | 143.10775 | 143.10769 | -0.5 | C8H16O2 |
| 23 | 1-Aminomethylphosphonic acid | [M+Cl]- | 145.97793 | 145.97832 | 2.6 | CH6NO3P |
| 24 | Cytosine | [M+Cl]- | 146.01266 | 146.01278 | 0.8 | C4H5N3O |
| 25 | Carvacrol | [M-H]- | 149.09719 | 149.09738 | 1.3 | C10H14O |
| 26 | 3-Mercapto-2-mercaptomethylpropanoate | [M-H]- | 150.98930 | 150.98959 | 1.9 | C4H8O2S2 |
| 27 | 2-Amino-4-nitrotoluene | [M-H]- | 151.05130 | 151.05123 | -0.5 | C7H8N2O2 |
| 28 | Heptan-1-ol | [M+Cl]- | 151.08952 | 151.08922 | -2.0 | C7H16O |
| 29 | (+)-(S)-Carvone | [M+H]+ | 151.11174 | 151.11212 | 2.5 | C10H14O |
| 30 | Thiocysteine | [M-H]- | 151.98454 | 151.98421 | -2.2 | C3H7NO2S2 |
| 31 | (+)-Camphor | [M+H]+ | 153.12739 | 153.12755 | 1.0 | C10H16O |
| 32 | (+)-Borneol | [M+H]+ | 155.14304 | 155.14305 | 0.1 | C10H18O |
| 33 | (+)-Neomenthol | [M-H]- | 155.14414 | 155.14402 | -0.8 | C10H20O |
| 34 | 2-Methylsulfonylpropane | [M+Cl]- | 156.00173 | 156.00137 | -2.3 | C4H9O2S |
| 35 | Dimercaprol | [M+Cl]- | 158.97106 | 158.97058 | -3.0 | C3H8OS2 |
| 36 | 1,4-Dihydroxynaphthalene | [M-H]- | 159.04515 | 159.04523 | 0.5 | C10H8O2 |
| 37 | (2-Naphthyl)methanol | [M+H]+ | 159.08044 | 159.08073 | 1.8 | C11H10O |
| 38 | (-)-Anabasine | [M+H]+ | 163.12297 | 163.12297 | 0.0 | C10H14N2 |
| 39 | 1-Hexenyl acetate | [M+Na]+ | 165.08860 | 165.08832 | -1.7 | C8H14O2 |
| 40 | Agmatine | [M+Cl]- | 165.09125 | 165.09104 | -1.3 | C5H14N4 |
| 41 | Homocysteinesulfinic acid | [M-H]- | 166.01795 | 166.01818 | 1.4 | C4H9NO4S |
| 42 | 2-Dimethylamino-5,6-dimethylpyrimidin-4-ol | [M+H]+ | 168.11314 | 168.11300 | -0.8 | C8H13N3O |
| 43 | 4-Aminobiphenyl | [M+H]+ | 170.09643 | 170.09675 | 1.9 | C12H11N |
| 44 | Lupinine | [M+H]+ | 170.15394 | 170.15357 | -2.2 | C10H19NO |
| 45 | (+)-Neomatatabiol | [M+H]+ | 171.13796 | 171.13776 | -1.1 | C10H18O2 |
| 46 | 3R-aminononanoic acid | [M-H]- | 172.13430 | 172.13413 | -1.0 | C9H19NO2 |
| 47 | 2,4-Diamino-6-hydroxylaminotoluene | [M+Na]+ | 176.07943 | 176.07922 | -1.2 | C7H11N3O |
| 48 | 7-Methylthioheptanaldoxime | [M+H]+ | 176.11036 | 176.11006 | -1.7 | C8H17NOS |

| 49 | 1,4-Dithiothreitol | [M+Na]+ | 177.00144 | 177.00148 | 0.2 | C4H10O2S2 |
| --- | --- | --- | --- | --- | --- | --- |
| 50 | Proline betaine | [M+Cl]- | 178.06403 | 178.06390 | -0.7 | C7H13NO2 |
| 51 | 1,1-Dimethylethyl benzoate | [M+H]+ | 179.10666 | 179.10642 | -1.3 | C11H14O2 |
| 52 | (-)-5-oxo-1,2-campholide | [M+H]+ | 183.10157 | 183.10117 | -2.2 | C10H14O3 |
| 53 | Ecgonine | [M-H]- | 184.09792 | 184.09806 | 0.8 | C9H15NO3 |
| 54 | 4,10-undecadiynal | [M+Na]+ | 185.09369 | 185.09417 | 2.6 | C11H14O |
| 55 | 1,2-Octanediol | [M+K]+ | 185.09384 | 185.09413 | 1.6 | C8H18O2 |
| 56 | 2,4,6-undecatrienal | [M+Na]+ | 187.10934 | 187.10921 | -0.7 | C11H16O |
| 57 | 10-amino-decanoic acid | [M+H]+ | 188.16451 | 188.16420 | -1.6 | C10H21NO2 |
| 58 | Diaminopimelic acid | [M-H]- | 189.08808 | 189.08842 | 1.8 | C7H14N2O4 |
| 59 | (+)-Bornane-2,5-dione | [M+Na]+ | 189.08860 | 189.08816 | -2.3 | C10H14O2 |
| 60 | 5,8,11-dodecatriynoic acid | [M+H]+ | 189.09101 | 189.09099 | -0.1 | C12H12O2 |
| 61 | (+)-Iridodial | [M+Na]+ | 191.10425 | 191.10378 | -2.4 | C10H16O2 |
| 62 | Linalool oxide | [M+Na]+ | 193.11990 | 193.11982 | -0.4 | C10H18O2 |
| 63 | 6-Hydroxypseudooxynicotine | [M-H]- | 193.09825 | 193.09822 | -0.2 | C10H14N2O2 |
| 64 | 4-Hydroxy-9-fluorenone | [M-H]- | 195.04515 | 195.04463 | -2.7 | C13H8O2 |
| 65 | 4-(2-Phenylethyl)phenol | [M-H]- | 197.09719 | 197.09718 | -0.1 | C14H14O |
| 66 | (R)-2-Methylimino-1-phenylpropan-1-ol | [M+Cl]- | 198.06912 | 198.06928 | 0.8 | C10H13NO |
| 67 | 2-methyl-undecanoic acid | [M-H]- | 199.17035 | 199.17000 | -1.8 | C12H24O2 |
| 68 | L-Methionine (R)-S-oxide | [M+Cl]- | 200.01537 | 200.01580 | 2.1 | C5H11NO3S |
| 69 | 2-Oxo-8-methylthiooctanoic acid | [M-H]- | 203.07474 | 203.07483 | 0.5 | C9H16O3S |
| 70 | Cuparene | [M+H]+ | 203.17943 | 203.18002 | 2.9 | C15H22 |
| 71 | 1,2-Dihydrostilbene | [M+Na]+ | 205.09877 | 205.09844 | -1.6 | C14H14 |
| 72 | 2,5-undecadienal | [M+K]+ | 205.09892 | 205.09880 | -0.6 | C11H18O |
| 73 | 10-undecynoic acid | [M+Na]+ | 205.11990 | 205.12025 | 1.7 | C11H18O2 |
| 74 | 10-hendecenoic acid | [M+Na]+ | 207.13555 | 207.13565 | 0.5 | C11H20O2 |
| 75 | Deoxyvasicinone | [M+Na]+ | 209.06853 | 209.06793 | -2.9 | C11H10N2O |
| 76 | (3R)-6-Hydroxy-3-isopropenyl-heptanoate | [M+Na]+ | 209.11482 | 209.11543 | 2.9 | C10H18O3 |
| 77 | 12-oxo-5E,8E,10Z-dodecatrienoic acid | [M+H]+ | 209.11722 | 209.11768 | 2.2 | C12H16O3 |
| 78 | (+)-cis-3,4-Dihydrophenanthrene-3,4-diol | [M-H]- | 211.07645 | 211.07613 | -1.5 | C14H12O2 |
| 79 | (R)-3-Hydroxydecanoic acid | [M+Na]+ | 211.13047 | 211.13030 | -0.8 | C10H20O3 |
| 80 | 1-Phenyl-5-mercaptotetrazole | [M+Cl]- | 213.00072 | 213.00016 | -2.6 | C7H6N4S |
| 81 | Benzyl nicotinate | [M+H]+ | 214.08626 | 214.08580 | -2.1 | C13H11NO2 |
| 82 | Methyl 2-diazoacetamidohexanoate | [M+H]+ | 215.12644 | 215.12586 | -2.7 | C9H16N3O3 |
| 83 | 12-amino-dodecanoic acid | [M+H]+ | 216.19581 | 216.19629 | 2.2 | C12H25NO2 |
| 84 | Bornyl acetate | [M+Na]+ | 219.13555 | 219.13520 | -1.6 | C12H20O2 |
| 85 | N-Acetyl-b-glucosaminylamine | [M+H]+ | 221.11320 | 221.11283 | -1.7 | C8H16N2O5 |
| 86 | 2-(3'-Methylthio)propylmalic acid | [M+H]+ | 223.06347 | 223.06284 | -2.8 | C8H14O5S |
| 87 | 2E-Decenedioic acid | [M+Na]+ | 223.09408 | 223.09421 | 0.6 | C10H16O4 |
| 88 | Anhalonidine | [M+H]+ | 224.12812 | 224.12790 | -1.0 | C12H17NO3 |
| 89 | Diethyl adipate | [M+Na]+ | 225.10973 | 225.10981 | 0.3 | C10H18O4 |
| 90 | (+)-7-Isomethyljasmonate | [M+H]+ | 225.14852 | 225.14866 | 0.6 | C13H20O3 |
| 91 | Myristic acid | [M-H]- | 227.20165 | 227.20231 | 2.9 | C14H28O2 |
| 92 | Deoxyuridine | [M+H]+ | 229.08190 | 229.08251 | 2.7 | C9H12N2O5 |
| 93 | 4-(Methylnitrosamino)-1-(3-pyridyl)-1-butanone | [M+Na]+ | 230.09000 | 230.09052 | 2.3 | C10H13N3O2 |
| 94 | 13-amino-tridecanoic acid | [M+H]+ | 230.21146 | 230.21097 | -2.1 | C13H27NO2 |
| 95 | 2,4,7-tridecatrienal | [M+K]+ | 231.11457 | 231.11507 | 2.1 | C13H20O |
| 96 | (+)-7-Isojasmonic acid | [M+Na]+ | 233.11482 | 233.11485 | 0.1 | C12H18O3 |
| 97 | (+)-7-epi--9,10-dihydrojasmonic acid | [M+Na]+ | 235.13047 | 235.12991 | -2.3 | C12H20O3 |
| 98 | 10-hydroxy-11-dodecenoic acid | [M+Na]+ | 237.14612 | 237.14572 | -1.7 | C12H22O3 |

| 99 | Succinyl proline | [M+Na]+ | 238.06859 | 238.06838 | -0.9 | C9H13NO5 |
| --- | --- | --- | --- | --- | --- | --- |
| 100 | (+)-12-methyl myristic acid | [M-H]- | 241.21730 | 241.21741 | 0.5 | C15H30O2 |
| 101 | 2,4-Dichlorophenoxyacetate | [M+Na]+ | 242.95862 | 242.95834 | -1.2 | C8H6Cl2O3 |
| 102 | (+)-2-Sterpurene-6-ol | [M+Na]+ | 243.17194 | 243.17140 | -2.2 | C15H24O |
| 103 | 1,3,5-Trihydroxyxanthone | [M+H]+ | 245.04445 | 245.04375 | -2.8 | C13H8O5 |
| 104 | 2,2'-Dihydroxy-4-methoxybenzophenone | [M+H]+ | 245.08084 | 245.08130 | 1.9 | C14H12O4 |
| 105 | (+)-Mayurone | [M+K]+ | 245.13022 | 245.13025 | 0.1 | C14H22O |
| 106 | 2-Aminoacridone | [M+K]+ | 249.04247 | 249.04250 | 0.1 | C13H10N2O |
| 107 | (-)-11-hydroxy-9,10-dihydrojasmonic acid | [M+Na]+ | 251.12538 | 251.12512 | -1.0 | C12H20O4 |
| 108 | 10-keto tridecanoic acid | [M+Na]+ | 251.16177 | 251.16204 | 1.1 | C13H24O3 |
| 109 | Cryptolepine | [M+Na]+ | 255.08927 | 255.08919 | -0.3 | C16H12N2 |
| 110 | Girgensonine | [M+K]+ | 255.08942 | 255.08919 | -0.9 | C13H16N2O |
| 111 | Palmitic Acid | [M-H]- | 255.23295 | 255.23264 | -1.2 | C16H32O2 |
| 112 | 7E,9E,11-Dodecatrienyl acetate | [M+Cl]- | 257.13138 | 257.13115 | -0.9 | C14H22O2 |
| 113 | 3,3'-Dimethylbisphenol A | [M+H]+ | 257.15361 | 257.15378 | 0.7 | C17H20O2 |
| 114 | 3-Deoxyestradiol | [M+H]+ | 257.18999 | 257.19066 | 2.6 | C18H24O |
| 115 | N-Lauroylglycine | [M+H]+ | 258.20637 | 258.20606 | -1.2 | C14H27NO3 |
| 116 | 1,9Z,16-heptadecatrien-4,6-diyn-3,8-diol | [M+H]+ | 259.16926 | 259.16898 | -1.1 | C17H22O2 |
| 117 | 3-Deoxy-D-manno-octulosonate | [M+Na]+ | 261.05809 | 261.05830 | 0.8 | C8H14O8 |
| 118 | Selagine | [M+Na]+ | 265.13113 | 265.13040 | -2.7 | C15H18N2O |
| 119 | N-Undecanoylglycine | [M+Na]+ | 266.17266 | 266.17207 | -2.2 | C13H25NO3 |
| 120 | Ungeremine | [M+H]+ | 267.08899 | 267.08881 | -0.7 | C16H12NO3 |
| 121 | Hexahomomethionine | [M+Cl]- | 268.11435 | 268.11469 | 1.3 | C11H23NO2S |
| 122 | 8-Azaadenosine | [M+H]+ | 269.09928 | 269.09957 | 1.1 | C9H12N6O4 |
| 123 | Goniothalenol | [M+K]+ | 271.03672 | 271.03598 | -2.7 | C13H12O4 |
| 124 | 17-Methyl-18-norandrosta-dien-3-one | [M+H]+ | 271.20564 | 271.20625 | 2.2 | C19H26O |
| 125 | 3-Oxo-steroid | [M-H]- | 271.20674 | 271.20628 | -1.7 | C19H28O |
| 126 | 7-Aminocephalosporanic acid | [M+H]+ | 273.05397 | 273.05459 | 2.3 | C10H12N2O5S |
| 127 | (4S,5S)-(+)-Germacrone 4,5-epoxide | [M+K]+ | 273.12514 | 273.12451 | -2.3 | C15H22O2 |
| 128 | Hexadecatrienoic acid | [M+Na]+ | 273.18250 | 273.18316 | 2.4 | C16H26O2 |
| 129 | 3-methyl-tetradecanedioic acid | [M+H]+ | 273.20604 | 273.20573 | -1.1 | C15H28O4 |
| 130 | 1-(3,4-Dihydroxyphenyl)-1-decene-3,5-dione | [M-H]- | 275.12888 | 275.12906 | 0.6 | C16H20O4 |
| 131 | 2-Ethylhexyl phthalate | [M+H]+ | 279.15909 | 279.15984 | 2.7 | C16H22O4 |
| 132 | 1-(3,4-Dihydroxyphenyl)-5-hydroxy-3-decanone | [M+H]+ | 281.17474 | 281.17487 | 0.5 | C16H24O4 |
| 133 | (11E)-Octadecenoic acid | [M-H]- | 281.24860 | 281.24810 | -1.8 | C18H34O2 |
| 134 | Coformycin | [M-H]- | 283.10479 | 283.10544 | 2.3 | C11H16N4O5 |
| 135 | Cadiamine | [M+H]+ | 283.20162 | 283.20162 | 0.0 | C15H26N2O3 |
| 136 | Stearic acid | [M-H]- | 283.26425 | 283.26486 | 2.1 | C18H36O2 |
| 137 | (-)-Sativan | [M-H]- | 285.11323 | 285.11304 | -0.7 | C17H18O4 |
| 138 | 4-Dodecylphenol | [M+Na]+ | 285.21889 | 285.21893 | 0.2 | C18H30O |
| 139 | Triamterene | [M+Cl]- | 288.07699 | 288.07632 | -2.4 | C12H11N7 |
| 140 | 16-Epivellosimine | [M-H]- | 291.15029 | 291.15100 | 2.5 | C19H20N2O |
| 141 | (1R,2R)-3-oxo-2-pentyl-cyclopentanehexanoic acid | [M+Na]+ | 291.19307 | 291.19317 | 0.4 | C16H28O3 |
| 142 | (+)-Elaeocarpine | [M+Cl]- | 292.11098 | 292.11166 | 2.3 | C16H19NO2 |
| 143 | Acetylsulfamethoxazole | [M-H]- | 294.05540 | 294.05594 | 1.8 | C12H13N3O4S |
| 144 | de-Hypoxanthine futalosine | [M-H]- | 295.08233 | 295.08158 | -2.5 | C14H16O7 |
| 145 | (1R,2R)-3-oxo-2-pentyl-cyclopentaneoctanoic acid | [M+H]+ | 297.24242 | 297.24213 | -1.0 | C18H32O3 |
| 146 | 2-Methyl-4-amino-5-hydroxymethylpyrimidine diphosphate | [M-H]- | 297.99995 | 298.00013 | 0.6 | C6H11N3O7P2 |
| 147 | 2'-Hydroxyfurano[2'',3'':4',3']chalcone | [M+Cl]- | 299.04805 | 299.04776 | -1.0 | C17H12O3 |

| 148 | 1-(4-Hydroxyphenyl)-1-decene-3,5-dione | [M+K]+ | 299.10440 | 299.10507 | 2.2 | C16H20O3 |
| --- | --- | --- | --- | --- | --- | --- |
| 149 | 6-Acetophenazine-1-carboxylic acid | [M+Cl]- | 301.03854 | 301.03782 | -2.4 | C15H10N2O3 |
| 150 | Octadecatrienoic acid | [M+Na]+ | 301.21380 | 301.21336 | -1.5 | C18H30O2 |
| 151 | 11,12,15-trihydroxy palmitic acid | [M-H]- | 303.21770 | 303.21837 | 2.2 | C16H32O5 |
| 152 | (R)-laballenic acid | [M+Na]+ | 303.22945 | 303.22984 | 1.3 | C18H32O2 |
| 153 | N-Acetyl-L-glutamate 5-phosphate | [M+Cl]- | 303.99945 | 303.99910 | -1.1 | C7H12NO8P |
| 154 | (+)-Medicarpin | [M+Cl]- | 305.05861 | 305.05856 | -0.2 | C16H14O4 |
| 155 | 16α-Fluoro-17α-hydroxyandrostenone | [M-H]- | 305.19223 | 305.19182 | -1.3 | C19H27FO2 |
| 156 | 3-Iodo-L-tyrosine | [M-H]- | 305.96326 | 305.96412 | 2.8 | C9H10INO3 |
| 157 | Lunamarine | [M-H]- | 308.09283 | 308.09325 | 1.4 | C18H15NO4 |
| 158 | ε-(γ-Glutamyl)-lysine | [M+Cl]- | 310.11752 | 310.11749 | -0.1 | C11H21N3O5 |
| 159 | 4'-Prenyloxyresveratrol | [M-H]- | 311.12888 | 311.12894 | 0.2 | C19H20O4 |
| 160 | (5α,17β)-3-Methyl-androst-2-en-17-ol | [M+Na]+ | 311.23454 | 311.23477 | 0.7 | C20H32O |
| 161 | (11Z)-8,18-ethanoretinal | [M+H]+ | 311.23694 | 311.23624 | -2.3 | C22H30O |
| 162 | 12-oxo-heptadecatrienoic acid | [M+Cl]- | 313.15760 | 313.15806 | 1.5 | C17H26O3 |
| 163 | 2-Isopropyl-3-(4-hydroxybenzoyl)benzofuran | [M+Cl]- | 315.07935 | 315.08027 | 2.9 | C18H16O3 |
| 164 | Dihydroxyoctadecenoic acid | [M+H]+ | 315.25299 | 315.25314 | 0.5 | C18H34O4 |
| 165 | 9-Riburonosyladenine | [M+Cl]- | 316.04542 | 316.04520 | -0.7 | C10H11N5O5 |
| 166 | (+)-18-Hydroxy-7,16-sacculatadiene-11,12-dial | [M-H]- | 317.21222 | 317.21214 | -0.2 | C20H30O3 |
| 167 | (4E,8E,10E-d18:3)sphingosine | [M+Na]+ | 318.24035 | 318.24095 | 1.9 | C18H33NO2 |
| 168 | 6'-Dehydro-6'-oxoparomamine | [M+H]+ | 322.16088 | 322.16087 | 0.0 | C12H23N3O7 |
| 169 | Succinylcholine | [M+Cl]- | 325.18996 | 325.19082 | 2.6 | C14H30N2O4 |
| 170 | L-α-Acetyl-N,N-dinormethadol | [M+H]+ | 326.21146 | 326.21237 | 2.8 | C21H27NO2 |
| 171 | 5b-Pregnanediol | [M+K]+ | 327.24486 | 327.24500 | 0.4 | C21H36 |
| 172 | 17-Methylandrosta-2,4-dieno[2,3-d]isoxazol-17β-ol | [M+H]+ | 328.22711 | 328.22698 | -0.4 | C21H29NO2 |
| 173 | (5Z,9E,14Z)-icosa-5,9,14-trienoic acid | [M+Na]+ | 329.24510 | 329.24489 | -0.6 | C20H34O2 |
| 174 | (2S,3S)-2-Hydroxytridecane-1,2,3-tricarboxylate | [M-H]- | 331.17623 | 331.17615 | -0.2 | C16H28O7 |
| 175 | 2,3,9,10-Tetrahydroxyberbine | [M+Cl]- | 334.08516 | 334.08531 | 0.5 | C17H17NO4 |
| 176 | Alamarine | [M-H]- | 337.11938 | 337.11841 | -2.9 | C19H18N2O4 |
| 177 | Belladine | [M+Na]+ | 338.17266 | 338.17263 | -0.1 | C19H25NO3 |
| 178 | Evocarpine | [M-H]- | 338.24894 | 338.24979 | 2.5 | C23H33NO |
| 179 | Benzyl viologen | [M+H]+ | 339.18558 | 339.18568 | 0.3 | C24H22N2 |
| 180 | 17-Hydroxy-3-oxo-17α-pregna-1,4-diene-21-carboxylic acid,γ-  lactone | [M-H]- | 339.19657 | 339.19688 | 0.9 | C22H28O3 |
| 181 | 5,7,4'-Trihydroxy-6-prenylisoflavanone | [M+H]+ | 341.13835 | 341.13810 | -0.7 | C20H20O5 |
| 182 | Propafenone | [M+H]+ | 342.20637 | 342.20651 | 0.4 | C21H27NO3 |
| 183 | 11(R)-HEDE | [M+Na]+ | 347.25567 | 347.25536 | -0.9 | C20H36O3 |
| 184 | Alatolide | [M-H]- | 349.16566 | 349.16629 | 1.8 | C19H26O6 |
| 185 | (-)-Tortuosamine | [M+Na]+ | 349.18865 | 349.18878 | 0.4 | C20H26N2O2 |
| 186 | Etretinate | [M-H]- | 353.21222 | 353.21135 | -2.5 | C23H30O3 |
| 187 | 2',4',4''-Trihydroxy-3',6'',6''-  trimethylpyrano[2'',3'':6',5']dihydrochalcone | [M-H]- | 355.15510 | 355.15547 | 1.1 | C21H24O5 |
| 188 | Oxybutynin | [M-H]- | 356.22312 | 356.22309 | -0.1 | C22H31NO3 |
| 189 | 9,10-Dihydrokadsurenone | [M-H]- | 357.17075 | 357.17078 | 0.1 | C21H26O5 |
| 190 | 11,13-dimethoxy-12-hydroxy-9-octadecenoic acid | [M+H]+ | 359.27920 | 359.27913 | -0.2 | C20H38O5 |
| 191 | 2,2,4-Trimethyl-3-(4-fluorophenyl)-2H-1-benzopyran-7-ol acetate | [M+Cl]- | 361.10122 | 361.10021 | -2.8 | C20H19FO3 |
| 192 | 7-Deoxyloganate | [M+H]+ | 361.14931 | 361.14895 | -1.0 | C16H24O9 |
| 193 | (Z)-11β,21-Dihydroxypregna-1,4,17(20)-trien-3-one | [M+Cl]- | 363.17325 | 363.17360 | 1.0 | C21H28O3 |
| 194 | N-palmitoyl taurine | [M+H]+ | 364.25161 | 364.25202 | 1.1 | C18H37NO4S |
| 195 | 1'H-5α-Androst-2-eno[3,2-b]indol-17β-ol | [M+H]+ | 364.26349 | 364.26327 | -0.6 | C25H33NO |

| 196 | Xanthurenate-8-O-β-D-glucoside | [M-H]- | 365.07523 | 365.07627 | 2.8 | C16H16NO9 |
| --- | --- | --- | --- | --- | --- | --- |
| 197 | (2S)-5,7-Dimethoxy-3',4'-methylenedioxyflavanone | [M+K]+ | 367.05785 | 367.05678 | -2.9 | C18H16O6 |
| 198 | Malvidin | [M+H]+ | 367.05791 | 367.05724 | -1.8 | C17H15ClO7 |
| 199 | 1-Dehydro-9-fluoro-11-oxotestololactone | [M+Cl]- | 367.11179 | 367.11182 | 0.1 | C19H21FO4 |
| 200 | Leonuridine | [M+Na]+ | 371.13125 | 371.13095 | -0.8 | C15H24O9 |
| 201 | 3-(2,4-Cyclopentadien-1-ylidene)-5α-androstan-17β-ol | [M+K]+ | 377.22413 | 377.22381 | -0.8 | C24H34O |
| 202 | 13,14-Dihydro PGE1 | [M+Na]+ | 379.24549 | 379.24571 | 0.6 | C20H36O5 |
| 203 | 3'-(2-Hydroxy-3-methylbut-3-enyl)-4,2',4'-trihydroxychalcone | [M+K]+ | 381.07350 | 381.07370 | 0.5 | C19H18O6 |
| 204 | 3',4',5-Trihydroxy-3,6,7-trimethoxyflavone | [M+Na]+ | 383.07374 | 383.07296 | -2.0 | C18H16O8 |
| 205 | 11-deoxy-11-methylene-15-keto-PGD2 | [M+Cl]- | 383.19946 | 383.20013 | 1.7 | C21H32O4 |
| 206 | Actinonin | [M-H]- | 384.25039 | 384.25019 | -0.5 | C19H35N3O5 |
| 207 | 19-epi-Cathenamine | [M+Cl]- | 385.13244 | 385.13189 | -1.4 | C21H22N2O3 |
| 208 | Fluoro-11-oxoprogesterone | [M+K]+ | 385.15758 | 385.15694 | -1.7 | C21H27FO3 |
| 209 | Fulvinervin B | [M+H]+ | 387.15909 | 387.15801 | -2.8 | C25H22O4 |
| 210 | 13,14-Dihydro- lipoxin A4 | [M+Cl]- | 388.20220 | 388.20130 | -2.3 | C20H33O5 |
| 211 | Tuberonic acid glucoside | [M+H]+ | 389.18061 | 389.18010 | -1.3 | C18H28O9 |
| 212 | N-eicosanoyl-ethanolamine | [M+Cl]- | 390.31443 | 390.31557 | 2.9 | C22H45NO2 |
| 213 | (-)-11-hydroxy-9,10-dihydrojasmonic acid 11-β-D-glucoside | [M+H]+ | 391.19626 | 391.19664 | 1.0 | C18H30O9 |
| 214 | Dihydroxy-cholenoic Acid | [M+H]+ | 391.28429 | 391.28413 | -0.4 | C24H38O4 |
| 215 | Dehydrodeguelin,7a,13a-Didehydrodeguelin | [M+H]+ | 393.13326 | 393.13370 | 1.1 | C23H20O6 |
| 216 | Lignoceryl alcohol | [M+K]+ | 393.34933 | 393.34863 | -1.8 | C24H50O |
| 217 | (6RS)-22-hydroxy-23,24,25,26,27-pentanorvitamin D3 6,19-sulfur  dioxide adduct | [M+H]+ | 395.22506 | 395.22565 | 1.5 | C22H34O4S |
| 218 | S-Adenosyl-4-methylthio-2-oxobutanoate | [M+H]+ | 399.12071 | 399.12175 | 2.6 | C15H20N5O6S |
| 219 | Dihydroxy--docosahexaenoic acid | [M+K]+ | 399.19322 | 399.19344 | 0.6 | C22H32O4 |
| 220 | C25 6,7-Epoxy highly branched isoprenoid | [M+Cl]- | 401.35557 | 401.35550 | -0.2 | C25H50O |
| 221 | (S)-N-[3-(3,4-Methylenedioxyphenyl)-2-(mercaptomethyl)-1-  oxoprolyl]-(S)-alanine | [M+Cl]- | 403.07361 | 403.07384 | 0.6 | C16H20N2O6S |
| 222 | 5S-HETE di-endoperoxide | [M+H]+ | 403.23264 | 403.23252 | -0.3 | C20H34O8 |
| 223 | 15-methyl-15R-PGF2α methyl ester | [M+Na]+ | 405.26115 | 405.26157 | 1.0 | C22H38O5 |
| 224 | PC(8:0/0:0) | [M+Na]+ | 406.19651 | 406.19679 | 0.7 | C16H34NO7P |
| 225 | N-stearoyl serine | [M+Cl]- | 406.27296 | 406.27197 | -2.4 | C21H41NO4 |
| 226 | 2,4,2'-Trihydroxy-6'',6''-dimethyl-3'-  prenylpyrano[2'',3'':4',5']chalcone | [M+H]+ | 407.18530 | 407.18594 | 1.6 | C25H26O5 |
| 227 | 4-Hydroxy-N-desmethyltamoxifen | [M+Cl]- | 408.17358 | 408.17334 | -0.6 | C25H27NO2 |
| 228 | 2-Geranyl-tetrahydroxydihydrochalcone | [M-H]- | 409.20205 | 409.20177 | -0.7 | C25H30O5 |
| 229 | Grayanotoxin I | [M+H]+ | 413.25338 | 413.25340 | 0.0 | C22H36O7 |
| 230 | LysoPC(10:0) | [M+H]+ | 413.25369 | 413.25463 | 2.3 | C18H39NO7P |
| 231 | N-stearoyl taurine | [M+Na]+ | 414.26485 | 414.26548 | 1.5 | C20H41NO4S |
| 232 | 3'-Geranyl-2',4',6'-trihydroxychalcone | [M+Na]+ | 415.18798 | 415.18761 | -0.9 | C25H28O4 |
| 233 | Alpinine | [M+H]+ | 416.20676 | 416.20641 | -0.9 | C23H29NO6 |
| 234 | PA(14:0/0:0) | [M+K]+ | 421.17520 | 421.17484 | -0.9 | C17H35O7P |
| 235 | Chitobiose | [M-H]- | 423.16203 | 423.16218 | 0.3 | C16H28N2O11 |
| 236 | (-)-epigallocatechin sulfate | [M+K]+ | 423.98610 | 423.98493 | -2.8 | C15H13O10S |
| 237 | PGD2-dihydroxypropanylamine | [M-H]- | 424.27046 | 424.26977 | -1.6 | C23H39NO6 |
| 238 | (2S)-5,7,2',4'-Tetrahydroxy-8-prenyl-5'-(1,1-dimethylallyl)flavanone | [M+H]+ | 425.19587 | 425.19473 | -2.7 | C25H28O6 |
| 239 | 1,25-dihydroxy-2,4-dinor-1,3-secovitamin D3 | [M+Cl]- | 425.28280 | 425.28181 | -2.3 | C25H42O3 |
| 240 | Hydroxy-γ-tocotrienol | [M+H]+ | 427.32067 | 427.32108 | 1.0 | C28H42O3 |
| 241 | CMP-2-aminoethylphosphonate | [M-H]- | 429.05819 | 429.05909 | 2.1 | C11H20N4O10P2 |

| 242 | (22E)-(24R,25R)-25,26-epoxy-1α,24-dihydroxy-22,23-  didehydrovitamin D3 | [M+H]+ | 429.29994 | 429.30066 | 1.7 | C27H40O4 |
| --- | --- | --- | --- | --- | --- | --- |
| 243 | 17-phenyl-trinor-PGF2α isopropyl ester | [M+H]+ | 431.27920 | 431.28038 | 2.7 | C26H38O5 |
| 244 | PC(4:0/4:0) | [M+Cl]- | 432.15596 | 432.15596 | 0.0 | C16H32NO8P |
| 245 | (10E)-19-methylvitamin D3 | [M+K]+ | 437.31803 | 437.31717 | -2.0 | C28H46O |
| 246 | (22R)-22,25-dihydroxyvitamin D3 | [M+Na]+ | 439.31827 | 439.31943 | 2.6 | C27H44O3 |
| 247 | Dimethamine | [M+K]+ | 447.21569 | 447.21522 | -1.0 | C24H32N4O2 |
| 248 | N-stearoyl tyrosine | [M+H]+ | 448.34214 | 448.34261 | 1.0 | C27H45NO4 |
| 249 | 1α-fluoro-hydroxy-hexadehydrovitamin D3 | [M+K]+ | 451.24092 | 451.24039 | -1.2 | C27H37FO2 |
| 250 | Vitamin D3 butyrate | [M+H]+ | 455.38836 | 455.38777 | -1.3 | C31H50O2 |
| 251 | Isomontanic acid | [M+Cl]- | 459.39743 | 459.39847 | 2.3 | C28H56O2 |
| 252 | PA(20:2/0:0) | [M-H]- | 461.26736 | 461.26787 | 1.1 | C23H43O7P |
| 253 | Plakinamine A | [M+K]+ | 461.32926 | 461.33014 | 1.9 | C29H46N2 |
| 254 | 7-Methylthioheptyl glucosinolate | [M-H]- | 462.09317 | 462.09435 | 2.5 | C15H29NO9S3 |
| 255 | N-arachidonoyl glutamine | [M+K]+ | 471.26197 | 471.26296 | 2.1 | C25H40N2O4 |
| 256 | N-arachidonoyl glutamic acid | [M+K]+ | 472.24598 | 472.24666 | 1.4 | C25H39NO5 |
| 257 | Amorilin | [M-H]- | 475.24900 | 475.24779 | -2.5 | C30H36O5 |
| 258 | (5Z)-4,4-difluoro-1α,25-dihydroxyvitamin D3 | [M+Na]+ | 475.29942 | 475.30050 | 2.3 | C27H42F2O3 |
| 259 | 13'-carboxy-α-tocotrienol | [M+Na]+ | 476.28971 | 476.28969 | 0.0 | C29H41O4 |
| 260 | (-)-Asbestinine 2 | [M+H]+ | 477.32107 | 477.32113 | 0.1 | C28H44O6 |
| 261 | N-(4-benzenesulfonamide) arachidonoyl amine | [M+Na]+ | 481.24953 | 481.24850 | -2.2 | C26H38N2O3S |
| 262 | MG(0:0/24:0/0:0) | [M+K]+ | 481.36537 | 481.36677 | 2.9 | C27H54O4 |
| 263 | N-Acetyl-leukotriene E4 | [M+H]+ | 482.25709 | 482.25818 | 2.3 | C25H39NO6S |
| 264 | Fusicoccin H | [M+H]+ | 483.29524 | 483.29669 | 3.0 | C26H42O8 |
| 265 | 24-(dimethoxyphosphoryl)-25,26,27-trinorvitamin D3 | [M+Cl]- | 485.25930 | 485.25871 | -1.2 | C26H43O4P |
| 266 | (6R)-25-hydroxyvitamin D3 6,19-sulfur dioxide adduct | [M+Na]+ | 487.28525 | 487.28437 | -1.8 | C27H44O4S |
| 267 | Hydroxyandrostane-one-D-glucuronide | [M+Na]+ | 489.24589 | 489.24473 | -2.4 | C25H38O8 |
| 268 | 20-Hydroxy-leukotriene E4 | [M+Cl]- | 490.20356 | 490.20391 | 0.7 | C23H37NO6S |
| 269 | LysoPC(14:0) | [M+Na]+ | 490.29041 | 490.29148 | 2.2 | C22H46NO7P |
| 270 | Ximaosteroid D | [M+H]+ | 491.30033 | 491.30096 | 1.3 | C28H42O7 |
| 271 | Epothilone D | [M+H]+ | 492.27782 | 492.27733 | -1.0 | C27H41NO5S |
| 272 | 4,2'-Dihydroxy-3,4',6'-trimethoxychalcone 4-glucoside | [M+H]+ | 493.17044 | 493.16947 | -2.0 | C24H28O11 |
| 273 | PA(20:4/0:0) | [M+Cl]- | 493.21274 | 493.21374 | 2.0 | C23H39O7P |
| 274 | 3-Sulfodeoxycholic acid | [M+K]+ | 497.19698 | 497.19724 | 0.5 | C23H38O7S |
| 275 | Phellodensin F | [M-H]- | 501.17662 | 501.17739 | 1.5 | C26H30O10 |
| 276 | Carpaine | [M+Na]+ | 501.36628 | 501.36683 | 1.1 | C28H50N2O4 |
| 277 | 20-acetoxy-clavulone I | [M-H]- | 503.22866 | 503.22866 | 0.0 | C27H36O9 |
| 278 | PG(18:4/0:0) | [M+H]+ | 505.25610 | 505.25579 | -0.6 | C24H41O9P |
| 279 | Oxethazaine | [M+K]+ | 506.27795 | 506.27837 | 0.8 | C28H41N3O3 |
| 280 | Dihydroxy-11-(3-hydroxy-1-propynyl)-9,11-didehydrovitamin D3 | [M+K]+ | 507.28712 | 507.28737 | 0.5 | C30H44O4 |
| 281 | N-oleoyl tryptophan | [M+K]+ | 507.29835 | 507.29871 | 0.7 | C29H44N2O3 |
| 282 | LysoPE(0:0/18:3) | [M+Cl]- | 510.23929 | 510.23829 | -2.0 | C23H42NO7P |
| 283 | (20S)-1α,25-dihydroxy-20-methoxy-26,27-dimethylvitamin D3 | [M+K]+ | 513.33407 | 513.33310 | -1.9 | C30H50O4 |
| 284 | 24,24-difluoro-1α,25-dihydroxy-26,27-dimethyl-24a-homovitamin  D3 | [M+Na]+ | 517.34637 | 517.34489 | -2.9 | C30H48F2O3 |
| 285 | Thr-Pro-Arg-Lys | [M+Na]+ | 523.29630 | 523.29619 | -0.2 | C21H40N8O6 |
| 286 | LysoPE(0:0/20:3) | [M+Na]+ | 526.29041 | 526.28905 | -2.6 | C25H46NO7P |
| 287 | Patuletin 3,3'-di-O-sulfate | [M+Cl]- | 526.93625 | 526.93774 | 2.8 | C16H12O14S2 |
| 288 | Eudesobovatol A | [M+Na]+ | 527.31318 | 527.31378 | 1.1 | C33H44O4 |
| 289 | DG(14:1/16:1/0:0) | [M-H]- | 535.43680 | 535.43622 | -1.1 | C33H60O5 |

| 290 | 11-Deoxocucurbitacin I | [M+K]+ | 539.27695 | 539.27839 | 2.7 | C30H44O6 |
| --- | --- | --- | --- | --- | --- | --- |
| 291 | 2-methyl-32,35-anhydrobacteriohopanetetrol | [M-H]- | 541.46262 | 541.46362 | 1.8 | C36H62O3 |
| 292 | Westiellamide | [M-H]- | 545.30931 | 545.30773 | -2.9 | C27H42N6O6 |
| 293 | O-6-deoxy-L-galactopyranosyl-(1->2)-O-b-D-galactopyranosyl-(1-  >3)-2-(acetylamino)-1,5-anhydro-2-deoxy-D-arabino-Hexenitol | [M+Cl]- | 546.15951 | 546.15929 | -0.4 | C20H33NO14 |
| 294 | Dihydrozeatin-9-N-glucoside-O-glucoside | [M+H]+ | 546.24058 | 546.24042 | -0.3 | C22H35N5O11 |
| 295 | PS(20:4/0:0) | [M+H]+ | 546.28265 | 546.28327 | 1.1 | C26H44NO9P |
| 296 | (24R)-11α,20,24-trihydroxyecdysone | [M+Cl]- | 547.26793 | 547.26927 | 2.4 | C27H44O9 |
| 297 | Gossypetin 8-glucoside-3-sulfate | [M-H]- | 559.03993 | 559.04150 | 2.8 | C21H20O16S |
| 298 | 35-aminobacteriohopane-tetrol | [M-H]- | 560.46843 | 560.46784 | -1.1 | C35H63NO4 |
| 299 | 1α,25-dihydroxy-25,25-diphenyl-26,27-dinorvitamin D3 | [M+Na]+ | 563.34957 | 563.35048 | 1.6 | C37H48O3 |
| 300 | PG(20:4/0:0) | [M+Cl]- | 567.24952 | 567.25059 | 1.9 | C26H45O9P |
| 301 | Hydroxy-6R-(S-glutamylcysteinyl)-eicosatetraenoic acid | [M+H]+ | 569.28911 | 569.29059 | 2.6 | C28H44N2O8S |
| 302 | Dihydroxyneurosporene | [M-H]- | 573.46770 | 573.46803 | 0.6 | C40H62O2 |
| 303 | Adonitoxigenin 3-O-L-rhamnoside | [M+Cl]- | 585.24720 | 585.24585 | -2.3 | C29H42O10 |
| 304 | OH-Demethylspheroidenone | [M+H]+ | 587.44587 | 587.44719 | 2.2 | C40H58O3 |
| 305 | DG(15:0/18:4/0:0) | [M+Na]+ | 597.44895 | 597.45026 | 2.2 | C36H62O5 |
| 306 | 12S-acetoxy-punaglandin 2 | [M-H]- | 599.22646 | 599.22736 | 1.5 | C29H41ClO11 |
| 307 | Oscillatoxin A | [M+Na]+ | 601.29832 | 601.29654 | -3.0 | C31H46O10 |
| 308 | (+)-23-methyl-tetracosanoic acid | [M-H]- | 605.62421 | 605.62452 | 0.5 | C41H82O2 |
| 309 | PA(O-16:0/13:0) | [M+Na]+ | 615.43601 | 615.43632 | 0.5 | C32H65O7P |
| 310 | DG(15:0/18:2/0:0) | [M+K]+ | 617.45418 | 617.45539 | 2.0 | C36H66O5 |
| 311 | PA(12:0/18:1) | [M+H]+ | 619.43333 | 619.43399 | 1.1 | C33H63O8P |
| 312 | 1',2'-Dihydro-1',2'-dihydroxy-4-ketotoruelene | [M+K]+ | 621.37046 | 621.36878 | -2.7 | C40H54O3 |
| 313 | (3S,5R,8R,3'R)-mutatoxanthin | [M+K]+ | 623.38611 | 623.38735 | 2.0 | C40H56O3 |
| 314 | PS(12:0/12:0) | [M+H]+ | 624.38711 | 624.38765 | 0.9 | C30H58NO10P |
| 315 | PG(13:0/12:0) | [M+H]+ | 625.40751 | 625.40846 | 1.5 | C31H61O10P |
| 316 | DG(14:0/20:4/0:0) | [M+K]+ | 627.43853 | 627.43793 | -1.0 | C37H64O5 |
| 317 | PA(12:0/19:1) | [M+H]+ | 633.44898 | 633.44941 | 0.7 | C34H65O8P |
| 318 | 4-Hydroxymytiloxanthin | [M+Na]+ | 637.38635 | 637.38744 | 1.7 | C40H54O5 |
| 319 | DG(15:0/20:4/0:0) | [M+Cl]- | 637.46043 | 637.45923 | -1.9 | C38H66O5 |
| 320 | (2S,2'S)-Oscillol | [M+K]+ | 639.38102 | 639.38168 | 1.0 | C40H56O4 |
| 321 | PE(12:0/15:1) | [M+Na]+ | 642.41053 | 642.41148 | 1.5 | C32H62NO8P |
| 322 | PA(O-16:0/14:1) | [M+K]+ | 643.40995 | 643.41187 | 3.0 | C33H65O7P |
| 323 | 6-O-(Glcb)-(25R)-5α-spirostan-triol | [M+Na]+ | 649.39222 | 649.39050 | -2.7 | C34H58O10 |
| 324 | DG(17:2/22:6/0:0) | [M-H]- | 649.48375 | 649.48568 | 3.0 | C42H66O5 |
| 325 | PI(20:0/0:0) | [M+Na]+ | 651.34798 | 651.34719 | -1.2 | C29H57O12P |
| 326 | PA(13:0/18:2) | [M+Na]+ | 653.41528 | 653.41353 | -2.7 | C34H63O8P |
| 327 | Pandaroside B | [M+Na]+ | 657.36092 | 657.35955 | -2.1 | C35H54O10 |
| 328 | Spongipregnoloside A | [M+Cl]- | 659.32036 | 659.31845 | -2.9 | C33H52O11 |
| 329 | 12-O-Palmitoyl-16-hydroxyphorbol 13-acetate | [M+H]+ | 661.43101 | 661.43282 | 2.7 | C38H60O9 |
| 330 | DG(17:2/20:5/0:0) | [M+K]+ | 663.43853 | 663.43873 | 0.3 | C40H64O5 |
| 331 | Glycinoprenol-9 | [M+Cl]- | 671.59032 | 671.58966 | -1.0 | C45H80O |
| 332 | PA(13:0/22:6) | [M+H]+ | 679.43333 | 679.43485 | 2.2 | C38H63O8P |
| 333 | PE(12:0/18:3) | [M+Na]+ | 680.42618 | 680.42692 | 1.1 | C35H64NO8P |
| 334 | 8-Hydroxyluteolin 4'-methyl ether 7-(6'''-acetylallosyl-(1->2)-  glucoside | [M-H]- | 681.16724 | 681.16520 | -3.0 | C30H34O18 |
| 335 | PA(P-16:0/17:2) | [M+K]+ | 681.42560 | 681.42733 | 2.5 | C36H67O7P |
| 336 | PA(O-16:0/18:2) | [M+Cl]- | 693.46314 | 693.46259 | -0.8 | C37H71O7P |
| 337 | Ergosteryl oleate | [M+Cl]- | 695.55393 | 695.55385 | -0.1 | C46H76O2 |

| 338 | Thalicarpine | [M+H]+ | 697.34834 | 697.34697 | -2.0 | C41H48N2O8 |
| --- | --- | --- | --- | --- | --- | --- |
| 339 | PC(10:0/20:0) | [M-H]- | 704.52358 | 704.52256 | -1.4 | C38H76NO8P |
| 340 | Kaempferol 3-[2''',3''',4'''-triacetyl-α-L-arabinopyranosyl-(1->6)-  glucoside] | [M-H]- | 705.16724 | 705.16715 | -0.1 | C32H34O18 |
| 341 | 1-tetradecanoyl-2-hexadecanoyl-sn-glycero-3-phosphosulfocholine | [M+H]+ | 709.48365 | 709.48191 | -2.5 | C37H73O8PS |
| 342 | PS(P-16:0/16:1) | [M+H]+ | 718.50175 | 718.49966 | -2.9 | C38H72NO9P |
| 343 | PE(13:0/22:6) | [M+H]+ | 722.47553 | 722.47557 | 0.1 | C40H68NO8P |
| 344 | PA(15:0/20:3) | [M+K]+ | 723.43617 | 723.43825 | 2.9 | C38H69O8P |
| 345 | Octaprenyl diphosphate | [M+H]+ | 723.45130 | 723.45243 | 1.6 | C40H68O7P2 |
| 346 | PE(12:0/20:3) | [M+K]+ | 724.43141 | 724.43309 | 2.3 | C37H68NO8P |
| 347 | Cyclic di-3',5'-guanylate | [M+Cl]- | 725.06427 | 725.06455 | 0.4 | C20H24N10O14P2 |
| 348 | Pseudoaconitine | [M+K]+ | 728.30429 | 728.30510 | 1.1 | C36H51NO12 |
| 349 | PA(16:0/22:2) | [M+H]+ | 729.54288 | 729.54285 | 0.0 | C41H77O8P |
| 350 | Myxochromide S2 | [M+H]+ | 737.42324 | 737.42404 | 1.1 | C39H56N6O8 |
| 351 | 1'-Hydroxy-4-keto-γ-carotene glucoside | [M+H]+ | 747.51943 | 747.51860 | -1.1 | C47H70O7 |
| 352 | PE(20:5/P-18:1) | [M+H]+ | 748.52757 | 748.52604 | -2.0 | C43H74NO7P |
| 353 | 1-Hexadecanoyl-2-(9Z-octadecenoyl)-sn-glycero-3-phospho-sn-  glycerol | [M+H]+ | 749.53271 | 749.53452 | 2.4 | C40H77O10P |
| 354 | PS(O-16:0/18:0) | [M+H]+ | 750.56435 | 750.56628 | 2.6 | C40H80NO9P |
| 355 | SM(d16:1/20:1) | [M+Na]+ | 751.57245 | 751.57433 | 2.5 | C41H81N2O6P |
| 356 | PG(17:0/14:1) | [M+K]+ | 762.46819 | 762.46870 | 0.7 | C37H74NO10P |
| 357 | PA(19:0/22:6) | [M+H]+ | 763.52723 | 763.52942 | 2.9 | C44H75O8P |
| 358 | PC(13:0/22:6) | [M+H]+ | 764.52248 | 764.52335 | 1.1 | C43H74NO8P |
| 359 | PG(14:0/22:6) | [M+H]+ | 767.48576 | 767.48463 | -1.5 | C42H71O10P |
| 360 | PS(12:0/20:3) | [M+K]+ | 768.42124 | 768.42120 | -0.1 | C38H68NO10P |
| 361 | PG(O-18:0/17:2) | [M+Na]+ | 769.53539 | 769.53626 | 1.1 | C41H79O9P |
| 362 | PI(P-16:0/13:0) | [M+Na]+ | 775.47319 | 775.47186 | -1.7 | C38H73O12P |
| 363 | PG(15:1/22:4) | [M-H]- | 781.50251 | 781.50281 | 0.4 | C43H75O10P |
| 364 | PA(20:0/22:0) | [M-H]- | 787.62223 | 787.62001 | -2.8 | C45H89O8P |
| 365 | UDP-N-acetyl-D-galactosamine 4,6-bissulfate | [M+Cl]- | 801.96460 | 801.96641 | 2.3 | C17H27N3O23P2S2 |
| 366 | PC(15:0/20:3) | [M+Cl]- | 804.53156 | 804.53142 | -0.2 | C43H80NO8P |
| 367 | PC(16:0/22:6) | [M+H]+ | 806.56943 | 806.56876 | -0.8 | C46H80NO8P |
| 368 | PA(20:3/22:6) | [M+K]+ | 809.45182 | 809.45021 | -2.0 | C45H71O8P |
| 369 | PG(17:0/22:4) | [M-H]- | 811.54946 | 811.55162 | 2.7 | C45H81O10P |
| 370 | PC(P-20:0/19:1) | [M-H]- | 812.65386 | 812.65260 | -1.5 | C47H92NO7P |
| 371 | PI(12:0/20:4) | [M+Na]+ | 825.45245 | 825.45157 | -1.1 | C41H71O13P |
| 372 | PI(12:0/21:0) | [M+H]+ | 825.54876 | 825.54864 | -0.1 | C42H81O13P |
| 373 | UDP-3-(3R-hydroxy-tetradecanoyl)-αD-glucosamine | [M+Cl]- | 826.23369 | 826.23409 | 0.5 | C29H51N3O18P2 |
| 374 | Cyanidin 3-O-[(6-O-malonyl-2-O-β-D-xylopyranosyl)-β-D-  glucopyranoside]-7-O-β-D-glucopyranoside | [M-H]- | 828.19659 | 828.19833 | 2.1 | C35H41O23 |
| 375 | PI(13:0/22:1) | [M-H]- | 849.54985 | 849.54962 | -0.3 | C44H83O13P |
| 376 | PC(16:1/22:2) | [M+K]+ | 850.57226 | 850.57431 | 2.4 | C46H86NO8P |
| 377 | PI(O-16:0/18:4) | [M+K]+ | 855.47842 | 855.47918 | 0.9 | C43H77O12P |
| 378 | PG(P-20:0/21:0) | [M+K]+ | 871.61888 | 871.62013 | 1.4 | C47H93O9P |
| 379 | Cyanidin 3-(6''-(E)-p-coumarylsambubioside)-5-glucoside | [M-H]- | 888.23297 | 888.23229 | -0.8 | C41H45O22 |
| 380 | PGP(18:0/18:0) | [M+Cl]- | 893.50812 | 893.50753 | -0.7 | C42H84O13P2 |
| 381 | PG(22:0/22:0) | [M+Na]+ | 913.68681 | 913.68496 | -2.0 | C50H99O10P |
| 382 | Perillyl-CoA | [M-H]- | 914.19675 | 914.19644 | -0.3 | C31H48N7O17P3S |
| 383 | PE(24:0/24:1) | [M+H]+ | 914.75723 | 914.75930 | 2.3 | C53H104NO8P |
| 384 | TG(16:0/20:5/20:5) | [M+K]+ | 937.66820 | 937.66899 | 0.8 | C59H94O6 |

| 385 | TG(17:0/20:5/22:6) | [M+H]+ | 939.74362 | 939.74375 | 0.1 | C62H98O6 |
| --- | --- | --- | --- | --- | --- | --- |
| 386 | Galabiosylceramide (d18:1/20:0) | [M+Na]+ | 940.66956 | 940.66809 | -1.6 | C50H95NO13 |
| 387 | PC(18:0/26:0) | [M+K]+ | 940.71312 | 940.71076 | -2.5 | C52H104NO8P |
| 388 | PI(17:0/22:1) | [M+Cl]- | 941.58913 | 941.59159 | 2.6 | C48H91O13P |
| 389 | TG(17:0/20:4/22:6) | [M+H]+ | 941.75927 | 941.75785 | -1.5 | C62H100O6 |
| 390 | PI(18:0/22:1) | [M+Na]+ | 943.62460 | 943.62531 | 0.7 | C49H93O13P |
| 391 | TG(16:0/19:0/22:4) | [M+Na]+ | 947.80381 | 947.80564 | 1.9 | C60H108O6 |
| 392 | TG(17:1/20:5/20:5) | [M+K]+ | 949.66820 | 949.66830 | 0.1 | C60H94O6 |
| 393 | PI(20:4/22:6) | [M+Na]+ | 953.51505 | 953.51411 | -1.0 | C51H79O13P |
| 394 | PI(O-20:0/22:2) | [M+Na]+ | 955.66099 | 955.66380 | 2.9 | C51H97O12P |
| 395 | PC(22:0/24:1) | [M+K]+ | 966.72877 | 966.72713 | -1.7 | C54H106NO8P |
| 396 | PI(P-20:0/22:2) | [M+K]+ | 969.61927 | 969.61811 | -1.2 | C51H95O12P |
| 397 | TG(20:5/20:5/22:6) | [M+H]+ | 971.71232 | 971.71249 | 0.2 | C65H94O6 |
| 398 | Malvidin 3-(6''-p-coumarylglucoside)-5-dimalonylglucoside | [M+Cl]- | 1008.19439 | 1008.19395 | -0.4 | C44H45O25 |
| 399 | Demissine | [M+H]+ | 1018.55812 | 1018.55982 | 1.7 | C50H83NO20 |
| 400 | CDP-DG(18:1/22:3) | [M-H]- | 1056.56962 | 1056.57210 | 2.4 | C52H89N3O15P2 |
| 401 | Docosanoyl-CoA | [M-H]- | 1088.43150 | 1088.43417 | 2.5 | C43H78N7O17P3S |
| 402 | Icosatetraenoyl-CoA | [M+K]+ | 1092.30803 | 1092.30815 | 0.1 | C41H66N7O17P3S |
| a Cer: Ceramide; GalCer: Galactosylceramide; GlcCer: Glucosylceramide; ; LacCer: Lactosylceramide; MG: Monoacylglycerol; DG: Diacylglycerol; TG: Triacylglycerol; MGDG: Monoacyldiacylglycerol; PA: Phosphatidic acid; PC: Phosphatidylcholine; PE: Phosphatidylethanolamine; PG(P): Glycerophospholipids; PI: Phosphatidylinositol; PS: Phosphatidylserine; SM: Sphingomyelin; CDP: Cytidine diphosphate; UDP: Uridine diphospate; SQMG: sulfoquinovosylmonoacylglycerols | | | | | | |
