# Supplementary material for: Satureja montana L. Essential Oils: Chemical Profiles/Phytochemical Screening, Antimicrobial Activity and O/W NanoEmulsion Formulations
Source: Pharmaceutics. 2019 Dec 19;12(1):7. doi: 10.3390/pharmaceutics12010007 (PMC7022231; doi:10.3390/pharmaceutics12010007)
Supplement: Supplementary file 1 [file pharmaceutics-12-00007-s001.zip › pharmaceutics-623925-SI-final/TableS4.docx]

| Table S4: SEOT Untargeted ESI FT-ICR Annotations | | | | | | |
| --- | --- | --- | --- | --- | --- | --- |
| No. | Compound (M)^a^ | Ion | Theor. m/z | Exp. m/z | Δppm | Formula |
| 1 | Butanal | [M+H]+ | 73.06479 | 73.06498 | 2.6 | C4H8O |
| 2 | Glycerone | [M-H]- | 89.02442 | 89.02442 | 0.0 | C3H6O3 |
| 3 | Aniline | [M+H]+ | 94.06513 | 94.06500 | -1.3 | C6H7N |
| 4 | Sulfate | [M-H]- | 96.96010 | 96.96037 | 2.8 | H2SO4 |
| 5 | Choline | [M]+ | 104.10699 | 104.10701 | -0.2 | C5H14NO |
| 6 | p-Xylene | [M+H]+ | 107.08553 | 107.08561 | 0.8 | C8H10 |
| 7 | Benzyl alcohol | [M+H]+ | 109.06479 | 109.06494 | 1.4 | C7H8O |
| 8 | 2,4-heptadienal | [M+H]+ | 111.08044 | 111.08059 | 1.3 | C7H10O |
| 9 | ε-Caprolactam | [M+H]+ | 114.09134 | 114.09136 | 0.2 | C6H11NO |
| 10 | Benzoate | [M-H]- | 121.02950 | 121.02922 | -2.3 | C7H6O2 |
| 11 | Phenylacetaldehyde | [M+H]+ | 121.06479 | 121.06496 | 1.4 | C8H8O |
| 12 | Cumene | [M+H]+ | 121.10118 | 121.10146 | 2.3 | C9H12 |
| 13 | N-Methyl-2-pyrrolidinone | [M+Na]+ | 122.05763 | 122.05759 | -0.4 | C5H9NO |
| 14 | Orcinol | [M+H]+ | 125.05971 | 125.05935 | -2.8 | C7H8O2 |
| 15 | 2,4-octadienal | [M+H]+ | 125.09609 | 125.09610 | 0.1 | C8H12O |
| 16 | Octylamine | [M+H]+ | 130.15903 | 130.15937 | 2.6 | C8H19N |
| 17 | Tolylacetonitrile | [M+H]+ | 132.08078 | 132.08060 | -1.3 | C9H9N |
| 18 | L-Leucine | [M+H]+ | 132.10191 | 132.10162 | -2.2 | C6H13NO2 |
| 19 | Tetralin | [M+H]+ | 133.10118 | 133.10125 | 0.5 | C10H12 |
| 20 | Cyclohexylamine | [M+Cl]- | 134.07420 | 134.07414 | -0.5 | C6H13N |
| 21 | p-Cymene | [M+H]+ | 135.11683 | 135.11683 | 0.0 | C10H14 |
| 22 | Terpinene | [M+H]+ | 137.13248 | 137.13238 | -0.7 | C10H16 |
| 23 | 2,4,6-Triaminotoluene | [M+H]+ | 138.10257 | 138.10236 | -1.5 | C7H11N3 |
| 24 | Nona-2,6-dienal | [M+H]+ | 139.11174 | 139.11198 | 1.7 | C9H14O |
| 25 | 1-Aminocyclopropane-1-carboxylate | [M+K]+ | 140.01084 | 140.01079 | -0.3 | C4H7NO2 |
| 26 | Nervonyl carnitine | [M+K]+ | 141.09143 | 141.09168 | 1.7 | C6H16N |
| 27 | Cycloheptanecarboxylic acid | [M+H]+ | 143.10666 | 143.10674 | 0.6 | C8H14O2 |
| 28 | 1-Phenylethylamine | [M+Na]+ | 144.07837 | 144.07861 | 1.7 | C8H11N |
| 29 | L-Lysinamide | [M-H]- | 144.11424 | 144.11386 | -2.6 | C6H15N3O |
| 30 | 3-Methyloxindole | [M+H]+ | 148.07569 | 148.07566 | -0.2 | C9H9NO |
| 31 | γ-Coniceine | [M+Na]+ | 148.10967 | 148.10948 | -1.3 | C8H15N |
| 32 | p-Cumic aldehyde | [M+H]+ | 149.09609 | 149.09638 | 1.9 | C10H12O |
| 33 | Carvacrol | [M-H]- | 149.09719 | 149.09718 | -0.1 | C10H14O |
| 34 | Galegine | [M+Na]+ | 150.10017 | 150.10059 | 2.8 | C6H13N3 |
| 35 | 3-Methyl-2-oxobutanoic acid | [M+Cl]- | 151.01675 | 151.01664 | -0.7 | C5H8O3 |
| 36 | (+)-Camphor | [M+H]+ | 153.12739 | 153.12751 | 0.8 | C10H16O |
| 37 | Bis(3-aminopropyl)amine | [M+Na]+ | 154.13147 | 154.13142 | -0.3 | C6H17N3 |
| 38 | Boschnialactone | [M+H]+ | 155.10666 | 155.10702 | 2.3 | C9H14O2 |
| 39 | Umbelliferone | [M+H]+ | 163.03897 | 163.03882 | -0.9 | C9H6O3 |
| 40 | 2,4-Dimethyl-2E,4E-heptadien-1-ol | [M+Na]+ | 163.10934 | 163.10960 | 1.6 | C9H16O |
| 41 | (R)-2-Methylimino-1-phenylpropan-1-ol | [M+H]+ | 164.10699 | 164.10700 | 0.1 | C10H13NO |
| 42 | p-Cumate | [M+H]+ | 165.09101 | 165.09100 | 0.0 | C10H12O2 |
| 43 | (+)-Bornane-2,5-dione | [M-H]- | 165.09210 | 165.09220 | 0.6 | C10H14O2 |
| 44 | 4-Trimethylammoniobutanal | [M+Cl]- | 165.09259 | 165.09220 | -2.4 | C7H16NO |
| 45 | Jasmone | [M+H]+ | 165.12739 | 165.12724 | -0.9 | C11H16O |
| 46 | Phthalate | [M+H]+ | 167.03389 | 167.03362 | -1.6 | C8H6O4 |
| 47 | 6-Oxocineole | [M-H]- | 167.10775 | 167.10749 | -1.6 | C10H16O2 |
| 48 | N-Cyclopropylammeline | [M+H]+ | 168.08799 | 168.08799 | 0.0 | C6H9N5O |
| 49 | Tetraethylammonium | [M+K]+ | 169.12273 | 169.12228 | -2.7 | C8H20N |
| 50 | Nitramine | [M+H]+ | 170.15394 | 170.15412 | 1.1 | C10H19NO |
| 51 | Dihydroxyfumarate | [M+Na]+ | 170.99001 | 170.99021 | 1.2 | C4H4O6 |
| 52 | 1,4-Diguanidinobutane | [M-H]- | 171.13637 | 171.13624 | -0.7 | C6H16N6 |
| 53 | N-Methylanthranilamide | [M+Na]+ | 173.06853 | 173.06851 | -0.1 | C8H10N2O |
| 54 | Eugenol methyl ether | [M+H]+ | 179.10666 | 179.10631 | -1.9 | C11H14O2 |
| 55 | 3-(4-Hydroxyphenyl)pyruvate | [M+H]+ | 181.04954 | 181.04928 | -1.4 | C9H8O4 |
| 56 | 3-tert-Butyl-5-methylcatechol | [M+H]+ | 181.12231 | 181.12201 | -1.6 | C11H16O2 |
| 57 | Fagomine | [M+Cl]- | 182.05894 | 182.05904 | 0.5 | C6H13NO3 |
| 58 | 5-Oxo-1,2-campholide | [M+H]+ | 183.10157 | 183.10188 | 1.7 | C10H14O3 |
| 59 | Butyro-betaine | [M+K]+ | 183.10200 | 183.10188 | -0.6 | C8H18NO |
| 60 | Geranyl formate | [M+H]+ | 183.13796 | 183.13760 | -1.9 | C11H18O2 |
| 61 | Trientine | [M+K]+ | 185.11631 | 185.11622 | -0.5 | C6H18N4 |
| 62 | 8-Amino-7-oxononanoate | [M+H]+ | 188.12812 | 188.12790 | -1.2 | C9H17NO3 |
| 63 | n-Decanohydroxamic acid | [M+H]+ | 188.16451 | 188.16430 | -1.1 | C10H21NO2 |
| 64 | 7,8-Diaminononanoate | [M+H]+ | 189.15975 | 189.15934 | -2.2 | C9H20N2O2 |
| 65 | 2-Isopropylmaleate | [M+Cl]- | 193.02731 | 193.02736 | 0.3 | C7H10O4 |

| 66 | Zingerone | [M-H]- | 193.08702 | 193.08659 | -2.2 | C11H14O3 |
| --- | --- | --- | --- | --- | --- | --- |
| 67 | Linalool oxide | [M+Na]+ | 193.11990 | 193.11960 | -1.6 | C10H18O2 |
| 68 | 3-(3,4-Dihydroxyphenyl)pyruvate | [M-H]- | 195.02990 | 195.03001 | 0.6 | C9H8O5 |
| 69 | L-Indospicine | [M+Na]+ | 196.10565 | 196.10566 | 0.1 | C7H15N3O2 |
| 70 | Suberic acid | [M+Na]+ | 197.07843 | 197.07846 | 0.2 | C8H14O4 |
| 71 | L-Cladinose | [M+Na]+ | 199.09408 | 199.09369 | -2.0 | C8H16O4 |
| 72 | Dodecanoic acid | [M-H]- | 199.17035 | 199.17024 | -0.6 | C12H24O2 |
| 73 | Tussilagine | [M+H]+ | 200.12812 | 200.12799 | -0.6 | C10H17NO3 |
| 74 | N-Methylnicotinium | [M+Na]+ | 200.12839 | 200.12799 | -2.0 | C11H17N2 |
| 75 | Capryloylglycine | [M+H]+ | 202.14377 | 202.14378 | 0.0 | C10H19NO3 |
| 76 | L-Methionine sulfoximine | [M+Na]+ | 203.04608 | 203.04662 | 2.6 | C5H12N2O3S |
| 77 | Chlorphenesin | [M+H]+ | 203.04695 | 203.04662 | -1.6 | C9H11ClO3 |
| 78 | α-Curcumene | [M+H]+ | 203.17943 | 203.17902 | -2.0 | C15H22 |
| 79 | (5-Phenyl-1,2,4-triazol-3-yl)urea | [M+H]+ | 204.08799 | 204.08842 | 2.1 | C9H9N5O |
| 80 | Diethyl (2R,3R)-2-methyl-3-hydroxysuccinate | [M+H]+ | 205.10705 | 205.10691 | -0.7 | C9H16O5 |
| 81 | 5-exo-Hydroxy-1,2-campholide | [M+Na]+ | 207.09917 | 207.09897 | -0.9 | C10H16O3 |
| 82 | 10-Oxodecanoate | [M+Na]+ | 209.11482 | 209.11487 | 0.3 | C10H18O3 |
| 83 | Azelaic acid | [M+Na]+ | 211.09408 | 211.09349 | -2.8 | C9H16O4 |
| 84 | 10-Hydroxydecanoic acid | [M+Na]+ | 211.13047 | 211.13073 | 1.3 | C10H20O3 |
| 85 | Cucurbic acid | [M+H]+ | 213.14852 | 213.14791 | -2.9 | C12H20O3 |
| 86 | O-Propanoylcarnitine | [M+H]+ | 218.13868 | 218.13920 | 2.4 | C10H19NO4 |
| 87 | 3,4-Dihydroxyfluorene | [M+Na]+ | 221.05730 | 221.05769 | 1.8 | C13H10O2 |
| 88 | 2,6-Dioxo-6-phenylhexanoate | [M+H]+ | 221.08084 | 221.08066 | -0.8 | C12H12O4 |
| 89 | Tridecanal | [M+Na]+ | 221.18759 | 221.18810 | 2.3 | C13H26O |
| 90 | 2-Succinylbenzoate | [M+H]+ | 223.06010 | 223.05947 | -2.8 | C11H10O5 |
| 91 | Sebacic acid | [M+Na]+ | 225.10973 | 225.11011 | 1.7 | C10H18O4 |
| 92 | 6-Benzylaminopurine | [M+H]+ | 226.10872 | 226.10940 | 3.0 | C12H11N5 |
| 93 | Myristoleic acid | [M+H]+ | 227.20056 | 227.20061 | 0.2 | C14H26O2 |
| 94 | Myristic acid | [M-H]- | 227.20165 | 227.20199 | 1.5 | C14H28O2 |
| 95 | N-Acetoxy-4-aminobiphenyl | [M+H]+ | 228.10191 | 228.10195 | 0.2 | C14H13NO2 |
| 96 | N-Decanoylglycine | [M+H]+ | 230.17507 | 230.17496 | -0.5 | C12H23NO3 |
| 97 | Xestoaminol C | [M+H]+ | 230.24784 | 230.24731 | -2.3 | C14H31NO |
| 98 | α-Ionone | [M+K]+ | 231.11457 | 231.11526 | 3.0 | C13H20O |
| 99 | 1,3,7-Trimethyluric acid | [M+Na]+ | 233.06451 | 233.06505 | 2.3 | C8H10N4O3 |
| 100 | Tert-butyloxycarbonyl-L-asparagine | [M+H]+ | 233.11320 | 233.11258 | -2.7 | C9H16N2O5 |
| 101 | (R)-(Homo)3-citrate | [M+H]+ | 235.08123 | 235.08066 | -2.4 | C9H14O7 |
| 102 | Pyrimidine nucleoside | [M+Na]+ | 236.07675 | 236.07726 | 2.1 | C9H13N2O4 |
| 103 | 6-Succinoaminopurine | [M+H]+ | 236.07782 | 236.07726 | -2.4 | C9H9N5O3 |
| 104 | Lophophorine | [M+H]+ | 236.12812 | 236.12829 | 0.7 | C13H17NO3 |
| 105 | 3-Oxododecanoic acid | [M+Na]+ | 237.14612 | 237.14608 | -0.1 | C12H22O3 |
| 106 | Gigantine | [M+H]+ | 238.14377 | 238.14327 | -2.1 | C13H19NO3 |
| 107 | Halfordinol | [M+H]+ | 239.08150 | 239.08215 | 2.7 | C14H10N2O2 |
| 108 | Undecanedioic acid | [M+Na]+ | 239.12538 | 239.12469 | -2.9 | C11H20O4 |
| 109 | Lipoamide | [M+Cl]- | 240.02891 | 240.02932 | 1.7 | C8H15NOS2 |
| 110 | 3-Hydroxysebacic acid | [M+Na]+ | 241.10464 | 241.10459 | -0.2 | C10H18O5 |
| 111 | (+)-12-methyl myristic acid | [M-H]- | 241.21730 | 241.21736 | 0.2 | C15H30O2 |
| 112 | 2,2-Dichloroethenyl dimethyl phosphate | [M+Na]+ | 242.93512 | 242.93514 | 0.1 | C4H7Cl2O4P |
| 113 | Falcarinone | [M+H]+ | 243.17434 | 243.17426 | -0.3 | C17H22O |
| 114 | Uridine | [M+H]+ | 245.07681 | 245.07701 | 0.8 | C9H12N2O6 |
| 115 | Apiole | [M+Na]+ | 245.07843 | 245.07780 | -2.6 | C12H14O4 |
| 116 | 2-Methylbutyroylcarnitine | [M+H]+ | 246.16998 | 246.17033 | 1.4 | C12H23NO4 |
| 117 | Citrinin | [M+H]+ | 251.09140 | 251.09169 | 1.2 | C13H14O5 |
| 118 | Butenylcarnitine | [M+Na]+ | 252.12063 | 252.12069 | 0.2 | C11H19NO4 |
| 119 | N-Ribosylnicotinamide | [M-H]- | 254.09082 | 254.09142 | 2.4 | C11H15N2O5 |
| 120 | Dyphylline | [M+H]+ | 255.10878 | 255.10858 | -0.8 | C10H14N4O4 |
| 121 | Palmitic acid | [M-H]- | 255.23295 | 255.23277 | -0.7 | C16H32O2 |
| 122 | Bakkenolide A | [M+Na]+ | 257.15120 | 257.15090 | -1.2 | C15H22O2 |
| 123 | 3-Dimethylallyl-4-hydroxymandelic acid | [M+Na]+ | 259.09408 | 259.09365 | -1.7 | C13H16O4 |
| 124 | Capsidiol | [M+Na]+ | 259.16685 | 259.16658 | -1.0 | C15H24O2 |
| 125 | 2-Heptyl-4-hydroxyquinoline-N-oxide | [M+H]+ | 260.16451 | 260.16437 | -0.5 | C16H21NO2 |
| 126 | 7-oxo-11E,13-Tetradecadienoic acid | [M+Na]+ | 261.14612 | 261.14670 | 2.2 | C14H22O3 |
| 127 | Centarol | [M+Na]+ | 261.18250 | 261.18321 | 2.7 | C15H26O2 |
| 128 | 3-Methylcholanthrene | [M+H]+ | 269.13248 | 269.13310 | 2.3 | C21H16 |
| 129 | 2-Deoxystreptidine | [M+Na]+ | 269.13326 | 269.13310 | -0.6 | C8H18N6O3 |
| 130 | Methyl palmitate | [M-H]- | 269.24860 | 269.24831 | -1.1 | C17H34O2 |
| 131 | 2R-aminohexadecanoic acid | [M-H]- | 270.24385 | 270.24329 | -2.1 | C16H33NO2 |
| 132 | (-)-Sparteine | [M+K]+ | 273.17276 | 273.17244 | -1.2 | C15H26N2 |

| 133 | 4,4'-Dihydroxy-3,5-dimethoxydihydrostilbene | [M+H]+ | 275.12779 | 275.12708 | -2.6 | C16H18O4 |
| --- | --- | --- | --- | --- | --- | --- |
| 134 | Podocarpic acid | [M+H]+ | 275.16417 | 275.16435 | 0.7 | C17H22O3 |
| 135 | Streptamine phosphate | [M+Na]+ | 281.05091 | 281.05114 | 0.8 | C6H15N2O7P |
| 136 | Xylobiose | [M-H]- | 281.08781 | 281.08845 | 2.3 | C10H18O9 |
| 137 | 8-Oxocoformycin | [M-H]- | 281.08914 | 281.08845 | -2.5 | C11H14N4O5 |
| 138 | 2-N-Undecyltetrahydrothiophene | [M+K]+ | 281.16998 | 281.16945 | -1.9 | C15H30S |
| 139 | all-trans-Dehydroretinal | [M+H]+ | 283.20564 | 283.20484 | -2.8 | C20H26O |
| 140 | Stearic acid | [M-H]- | 283.26425 | 283.26442 | 0.6 | C18H36O2 |
| 141 | Retinal | [M+H]+ | 285.22129 | 285.22137 | 0.3 | C20H28O |
| 142 | Retinol | [M+H]+ | 287.23694 | 287.23736 | 1.5 | C20H30O |
| 143 | N-Succinyl-LL-2,6-diaminoheptanedioate | [M-H]- | 289.10412 | 289.10460 | 1.6 | C11H18N2O7 |
| 144 | 12-Oxo-phytodienoic acid | [M+H]+ | 293.21112 | 293.21082 | -1.0 | C18H28O3 |
| 145 | D-Fructose 6-phosphate | [M+Cl]- | 294.99912 | 294.99906 | -0.2 | C6H13O9P |
| 146 | 7-Methylxanthosine | [M-H]- | 298.09188 | 298.09179 | -0.3 | C11H15N4O6 |
| 147 | Stearidonic acid | [M+Na]+ | 299.19815 | 299.19877 | 2.1 | C18H28O2 |
| 148 | Benzarone | [M+Cl]- | 301.06370 | 301.06300 | -2.3 | C17H14O3 |
| 149 | 11,11-Difluoro-9Z-dodecenyl acetate | [M+K]+ | 301.13760 | 301.13733 | -0.9 | C14H24F2O2 |
| 150 | γ-Linolenic acid | [M+Na]+ | 301.21380 | 301.21461 | 2.7 | C18H30O2 |
| 151 | MG(0:0/14:0/0:0) | [M-H]- | 301.23843 | 301.23753 | -3.0 | C17H34O4 |
| 152 | α-Ethyl-α,β-diphenyl-2-pyridineethanol | [M-H]- | 302.15504 | 302.15444 | -2.0 | C21H21NO |
| 153 | Abietate | [M+H]+ | 303.23186 | 303.23218 | 1.1 | C20H30O2 |
| 154 | Prosafrinine | [M+Na]+ | 306.24035 | 306.24016 | -0.6 | C17H33NO2 |
| 155 | Hydroxyestradiol-17β | [M+Na]+ | 311.16177 | 311.16103 | -2.4 | C18H24O3 |
| 156 | 3-methyl-tetradecanedioic acid | [M+K]+ | 311.16192 | 311.16103 | -2.9 | C15H28O4 |
| 157 | 1-Dodecanoyl-sn-glycerol | [M+K]+ | 313.17757 | 313.17719 | -1.2 | C15H30O4 |
| 158 | 2-chlorohexadecanol | [M+K]+ | 315.18515 | 315.18583 | 2.1 | C16H33ClO |
| 159 | [6]-Paradol | [M+K]+ | 317.15135 | 317.15166 | 1.0 | C17H26O3 |
| 160 | Gibberellin A12 aldehyde | [M+H]+ | 317.21112 | 317.21153 | 1.3 | C20H28O3 |
| 161 | (4E,8E,10E-d18:3)sphingosine | [M+Na]+ | 318.24035 | 318.23971 | -2.0 | C18H33NO2 |
| 162 | N-hydroxy arachidonoyl amine | [M+H]+ | 320.25841 | 320.25887 | 1.4 | C20H33NO2 |
| 163 | Tetraneurin E | [M-H]- | 323.15001 | 323.15019 | 0.6 | C17H24O6 |
| 164 | Triphenyl phosphate | [M-H]- | 325.06352 | 325.06445 | 2.9 | C18H15O4P |
| 165 | L-α-Acetyl-N,N-dinormethadol | [M+H]+ | 326.21146 | 326.21083 | -1.9 | C21H27NO2 |
| 166 | Farnesylcysteine | [M+H]+ | 326.21483 | 326.21419 | -2.0 | C18H31NO2S |
| 167 | 8-(3,3-Dimethylallyl)spatheliachromene | [M+H]+ | 327.15909 | 327.15959 | 1.5 | C20H22O4 |
| 168 | 16β-Fluoroandrost-4-ene-3,17-dione | [M+Na]+ | 327.17308 | 327.17401 | 2.8 | C19H25FO2 |
| 169 | Promegestone | [M+H]+ | 327.23186 | 327.23120 | -2.0 | C22H30O2 |
| 170 | 3-Methyl-19-nor-17α-pregna-1,3,5(10)-trien-17-ol | [M+Cl]- | 333.19907 | 333.19947 | 1.2 | C21H30O |
| 171 | 13-Hydroperoxyoctadeca-9,11-dienoic acid | [M+Na]+ | 335.21928 | 335.21940 | 0.4 | C18H32O4 |
| 172 | Steroid O-sulfate | [M+H]+ | 337.14681 | 337.14676 | -0.1 | C18H24O4S |
| 173 | Discadenine | [M+Cl]- | 339.13418 | 339.13351 | -2.0 | C14H20N6O2 |
| 174 | Benzyl viologen | [M+H]+ | 339.18558 | 339.18539 | -0.5 | C24H22N2 |
| 175 | Tetrabenazine | [M+Na]+ | 340.18831 | 340.18745 | -2.5 | C19H27NO3 |
| 176 | 2,6 dimethylheptanoyl carnitine | [M+K]+ | 340.18847 | 340.18745 | -3.0 | C16H31NO4 |
| 177 | N2,N5-Dibenzoyl-L-ornithine | [M+H]+ | 341.14958 | 341.14978 | 0.6 | C19H20N2O4 |
| 178 | 15S-hydroperoxy-eicosadienoic acid | [M+H]+ | 341.26864 | 341.26786 | -2.3 | C20H36O4 |
| 179 | Propafenone | [M+H]+ | 342.20637 | 342.20699 | 1.8 | C21H27NO3 |
| 180 | Hydroxy-eicosatetraenoate | [M+Na]+ | 343.22437 | 343.22486 | 1.4 | C20H32O3 |
| 181 | α,α'-Diethyl-4,4'-bis(2-propynyloxy)stilbene | [M+H]+ | 345.18491 | 345.18490 | 0.0 | C24H24O2 |
| 182 | (Hydroxymethyl)-androstane-diol | [M+Na]+ | 345.24002 | 345.24095 | 2.7 | C20H34O3 |
| 183 | Thebaine | [M+Cl]- | 346.12155 | 346.12114 | -1.2 | C19H21NO3 |
| 184 | Trimethyl-(fluorophenyl)-benzopyranol acetate | [M+Na]+ | 349.12104 | 349.12197 | 2.7 | C20H19FO3 |
| 185 | Schizonepetoside E | [M+H]+ | 349.18569 | 349.18527 | -1.2 | C16H28O8 |
| 186 | 10-nitro-octadecenoic acid | [M+Na]+ | 350.23018 | 350.23115 | 2.8 | C18H33NO4 |
| 187 | Epoxypregn-ene-3,20-dione | [M+Na]+ | 351.19307 | 351.19357 | 1.4 | C21H28O3 |
| 188 | Anacrotine | [M+H]+ | 352.17546 | 352.17512 | -1.0 | C18H25NO6 |
| 189 | 4,8 dimethylnonanoyl carnitine | [M+Na]+ | 352.24583 | 352.24582 | 0.0 | C18H35NO4 |
| 190 | 2-Caffeoylisocitrate | [M+H]+ | 355.06597 | 355.06622 | 0.7 | C15H14O10 |
| 191 | (S)-N-Methylcanadine | [M+H]+ | 355.17781 | 355.17809 | 0.8 | C21H24NO4 |
| 192 | sn-glycero-3-Phospho-1-inositol | [M+Na]+ | 357.05572 | 357.05650 | 2.2 | C9H19O11P |
| 193 | Aphidicolin | [M+Na]+ | 361.23493 | 361.23545 | 1.4 | C20H34O4 |
| 194 | Epoxy-fluoro-11β-hydroxypregn-4-ene-3,20-dione | [M+H]+ | 363.19661 | 363.19622 | -1.1 | C21H27FO4 |
| 195 | Dioctyl adipate | [M+H]+ | 371.31559 | 371.31513 | -1.2 | C22H42O4 |
| 196 | 3,3-Difluoro-5α-androstan-17β-yl acetate | [M+Na]+ | 377.22626 | 377.22715 | 2.4 | C21H32F2O2 |
| 197 | Cinncassiol C3 | [M+H]+ | 383.20643 | 383.20732 | 2.3 | C20H30O7 |
| 198 | 16-Methoxy-2,3-dihydro-3-hydroxytabersonine | [M+H]+ | 385.21218 | 385.21184 | -0.9 | C22H28N2O4 |
| 199 | Tetrahydrocorticosterone | [M+Cl]- | 385.21511 | 385.21452 | -1.5 | C21H34O4 |

| 200 | (7R)-7-(4-Carboxybutanamido)cephalosporanate | [M+H]+ | 387.08566 | 387.08589 | 0.6 | C15H18N2O8S |
| --- | --- | --- | --- | --- | --- | --- |
| 201 | Nitidine | [M+K]+ | 387.08674 | 387.08589 | -2.2 | C21H18NO4 |
| 202 | α-Linoleoylcholine | [M+Na]+ | 389.32643 | 389.32564 | -2.0 | C23H44NO2 |
| 203 | Aniflorine | [M+K]+ | 390.12145 | 390.12193 | 1.2 | C20H21N3O3 |
| 204 | PC(7:0/0:0) | [M+Na]+ | 392.18086 | 392.18075 | -0.3 | C15H32NO7P |
| 205 | N-stearoyl taurine | [M+H]+ | 392.28291 | 392.28302 | 0.3 | C20H41NO4S |
| 206 | Undecylprodigiosin | [M+H]+ | 394.28529 | 394.28441 | -2.2 | C25H35N3O |
| 207 | Tetradecanoylcarnitine | [M+Na]+ | 394.29278 | 394.29198 | -2.0 | C21H41NO4 |
| 208 | 4α-Methyl-5α-cholest-7-en-3-one | [M-H]- | 397.34759 | 397.34700 | -1.5 | C28H46O |
| 209 | N-palmitoyl taurine | [M+K]+ | 402.20749 | 402.20726 | -0.6 | C18H37NO4S |
| 210 | Cypridina luciferin | [M-H]- | 404.22043 | 404.22128 | 2.1 | C22H27N7O |
| 211 | Ginkgolide A | [M-H]- | 407.13476 | 407.13508 | 0.8 | C20H24O9 |
| 212 | didesmethyl tocotrienol | [M+K]+ | 407.23469 | 407.23565 | 2.4 | C25H36O2 |
| 213 | Lasiocarpine | [M+H]+ | 412.23298 | 412.23358 | 1.5 | C21H33NO7 |
| 214 | 9-Fluoroprednisolone | [M+Cl]- | 413.15365 | 413.15432 | 1.6 | C21H27FO5 |
| 215 | Lithocholic acid | [M+K]+ | 415.26090 | 415.26187 | 2.3 | C24H40O3 |
| 216 | (6RS)-22-hydroxy-23,24,25,26,27-pentanorvitamin D3 6,19-sulfur  dioxide adduct | [M+Na]+ | 417.20700 | 417.20679 | -0.5 | C22H34O4S |
| 217 | Diisononyl phthalate | [M+H]+ | 419.31559 | 419.31535 | -0.6 | C26H42O4 |
| 218 | DG(18:0e/2:0/0:0) | [M+Cl]- | 421.30901 | 421.30899 | -0.1 | C23H46O4 |
| 219 | Cortisone acetate | [M+Na]+ | 425.19346 | 425.19466 | 2.8 | C23H30O6 |
| 220 | Oleandolide | [M+K]+ | 425.19361 | 425.19466 | 2.5 | C20H34O7 |
| 221 | Dimethyl-(methoxyphenyl)-ethyl-hydroxy-benzopyran-methanol  diacetate | [M+H]+ | 425.19587 | 425.19466 | -2.8 | C25H28O6 |
| 222 | Glutinone | [M+H]+ | 425.37779 | 425.37721 | -1.4 | C30H48O |
| 223 | Aspidoalbine | [M+H]+ | 429.23840 | 429.23794 | -1.1 | C24H32N2O5 |
| 224 | Glycoperine | [M+K]+ | 430.08988 | 430.09103 | 2.7 | C19H21NO8 |
| 225 | Leu-leu-tyr | [M+Na]+ | 430.23124 | 430.23205 | 1.9 | C21H33N3O5 |
| 226 | 4α-hydroxymethyl-5α-cholesta-8,24-dien-3β-ol | [M+Na]+ | 436.33118 | 436.33133 | 0.4 | C28H45O2 |
| 227 | Polhovolide | [M+H]+ | 437.21699 | 437.21753 | 1.2 | C23H32O8 |
| 228 | 1α,25-dihydroxy-3-deoxy-3-thiavitamin D3 | [M+Na]+ | 441.27977 | 441.28054 | 1.7 | C26H42O2S |
| 229 | Palmitoyl glucuronide | [M+Na]+ | 441.28227 | 441.28186 | -0.9 | C22H42O7 |
| 230 | 4,4'-Diapophytofluene | [M+K]+ | 445.32311 | 445.32262 | -1.1 | C30H46 |
| 231 | 13'-Hydroxy-gama-tocotrienol | [M+Na]+ | 449.30262 | 449.30157 | -2.3 | C28H42O3 |
| 232 | MG(0:0/22:2/0:0) | [M+K]+ | 449.30277 | 449.30157 | -2.7 | C25H46O4 |
| 233 | methyl 5-hydroperoxy-bisepidioxy-eicosadienoate | [M+K]+ | 453.18853 | 453.18788 | -1.4 | C21H34O8 |
| 234 | N-arachidonoyl glutamic acid | [M+Na]+ | 456.27204 | 456.27290 | 1.9 | C25H39NO5 |
| 235 | Stoloniferone J | [M-H]- | 457.33233 | 457.33193 | -0.9 | C29H46O4 |
| 236 | Chitobiose | [M+Cl]- | 459.13871 | 459.13780 | -2.0 | C16H28N2O11 |
| 237 | Melithiazol A | [M+K]+ | 461.09656 | 461.09727 | 1.5 | C20H26N2O4S2 |
| 238 | D-Glucosyldihydrosphingosine | [M+H]+ | 464.35818 | 464.35855 | 0.8 | C24H49NO7 |
| 239 | Kaempferol 3-p-coumarate | [M+Cl]- | 467.05392 | 467.05427 | 0.8 | C24H16O8 |
| 240 | Eriotriochin | [M+H]+ | 467.20643 | 467.20738 | 2.0 | C27H30O7 |
| 241 | Staurosporine | [M+H]+ | 467.20777 | 467.20738 | -0.8 | C28H26N4O3 |
| 242 | PG(15:1/0:0) | [M+H]+ | 469.25610 | 469.25748 | 2.9 | C21H41O9P |
| 243 | 4,4'-Diaponeurosporenic acid | [M+K]+ | 471.26599 | 471.26688 | 1.9 | C30H40O2 |
| 244 | (20R,24R)-20-fluoro-1α,24-dihydroxy-26,27-cyclovitamin D3 | [M+K]+ | 471.26713 | 471.26688 | -0.5 | C27H41FO3 |
| 245 | 4,4-difluoro-1α,25-dihydroxyvitamin D3 | [M+Na]+ | 475.29942 | 475.30054 | 2.4 | C27H42F2O3 |
| 246 | 10-Deoxymethymycin | [M+Na]+ | 476.29826 | 476.29850 | 0.5 | C25H43NO6 |
| 247 | (6R)-vitamin D3 6,19-sulfur dioxide adduct | [M+K]+ | 487.26428 | 487.26291 | -2.8 | C27H44O3S |
| 248 | PA(20:1/0:0) | [M+Na]+ | 487.27951 | 487.28046 | 1.9 | C23H45O7P |
| 249 | PA(20:4e/2:0) | [M+H]+ | 487.28192 | 487.28046 | -3.0 | C25H43O7P |
| 250 | C17 sphingosine-1-phosphocholine | [M+K]+ | 489.28542 | 489.28628 | 1.8 | C22H47N2O5P |
| 251 | Ximaosteroid D | [M+H]+ | 491.30033 | 491.29911 | -2.5 | C28H42O7 |
| 252 | PG(18:4/0:0) | [M+H]+ | 505.25610 | 505.25716 | 2.1 | C24H41O9P |
| 253 | Brosimone H | [M+H]+ | 505.25847 | 505.25716 | -2.6 | C31H36O6 |
| 254 | Propapyriogenin A2 | [M+Na]+ | 507.30810 | 507.30918 | 2.1 | C30H44O5 |
| 255 | Scymnol | [M+K]+ | 507.30825 | 507.30918 | 1.8 | C27H48O6 |
| 256 | N-stearoyl tryptophan | [M+K]+ | 509.31400 | 509.31504 | 2.0 | C29H46N2O3 |
| 257 | Ajugalactone | [M+H]+ | 517.27959 | 517.28095 | 2.6 | C29H40O8 |
| 258 | Dracorubin | [M+Cl]- | 523.13178 | 523.13214 | 0.7 | C32H24O5 |
| 259 | O-1,4-α-L-Dihydrostreptosyl-streptidine 6-phosphate | [M+Cl]- | 523.13259 | 523.13214 | -0.9 | C14H29N6O11P |
| 260 | Norselic acid E | [M+Cl]- | 529.27263 | 529.27292 | 0.6 | C31H42O5 |
| 261 | 2-Octaprenyl-3-methyl-6-methoxy-1,4-benzoquinol | [M+Cl]- | 599.42365 | 599.42538 | 2.9 | C38H60O3 |
| 262 | 11-(4-acetoxymethylphenyl)-1α,25-dihydroxy-9,11-didehydrovitamin  D3 | [M+K]+ | 601.32898 | 601.32861 | -0.6 | C36H50O5 |

| 263 | Kaempferol 3-(2''-galloyl-α-L-arabinopyranoside) | [M+K]+ | 609.06411 | 609.06588 | 2.9 | C27H22O14 |
| --- | --- | --- | --- | --- | --- | --- |
| 264 | Peonidin 3-(6''-p-coumarylglucoside) | [M+H]+ | 610.16809 | 610.16640 | -2.8 | C31H29O13 |
| 265 | Glutathione disulfide | [M+H]+ | 613.15924 | 613.15952 | 0.5 | C20H32N6O12S2 |
| 266 | Formononetin 7-O-rutinoside | [M+K]+ | 615.14745 | 615.14644 | -1.6 | C28H32O13 |
| 267 | Isorhamnetin 3-(6''-galloylglucoside) | [M+Na]+ | 653.11131 | 653.11255 | 1.9 | C29H26O16 |
| 268 | PE(14:0/15:0) | [M+Na]+ | 672.45748 | 672.45718 | -0.4 | C34H68NO8P |
| 269 | PA(O-16:0/17:2) | [M+K]+ | 683.44125 | 683.44102 | -0.3 | C36H69O7P |
| 270 | PA(O-20:0/14:1) | [M+Na]+ | 683.49861 | 683.49919 | 0.8 | C37H73O7P |
| 271 | PA(O-18:0/18:4) | [M+H]+ | 683.50102 | 683.49919 | -2.7 | C39H71O7P |
| 272 | DG(16:0/22:4/0:0) | [M+K]+ | 683.50113 | 683.49919 | -2.8 | C41H72O5 |
| 273 | Vitexin 3''',4'''-Di-O-acetyl 2''-O-rhamnoside | [M+Na]+ | 685.17391 | 685.17489 | 1.4 | C31H34O16 |
| 274 | Embigenin 2''-(2'''-acetylrhamnoside) | [M+K]+ | 687.16858 | 687.17035 | 2.6 | C31H36O15 |
| 275 | Platycarpanetin 7-O-laminaribioside | [M+Na]+ | 689.16882 | 689.17033 | 2.2 | C30H34O17 |
| 276 | Pelargonidin 3-O-[2-O-(β-D-xylopyranosyl)-6-O-(malonyl)-beta-D-  galactopyranoside] | [M+K]+ | 690.11928 | 690.11996 | 1.0 | C29H31O17 |
| 277 | 1,2-ditetradecanoyl-sn-glycero-3-phosphosulfocholine | [M+Na]+ | 703.43430 | 703.43383 | -0.7 | C35H69O8PS |
| 278 | Quercetin 3-(2''-p-hydroxybenzoyl-4''-p-coumarylrhamnoside) | [M+H]+ | 715.16575 | 715.16531 | -0.6 | C37H30O15 |
| 279 | Tetrahydropteroyltri-L-glutamate | [M+K]+ | 742.21933 | 742.21751 | -2.4 | C29H37N9O12 |
| 280 | PS(P-16:0/17:2) | [M+Na]+ | 752.48369 | 752.48347 | -0.3 | C39H72NO9P |
| 281 | PS(14:0/20:4) | [M+H]+ | 756.48101 | 756.48185 | 1.1 | C40H70NO10P |
| 282 | P1,P2-Bis(5'-adenosyl) triphosphate | [M+H]+ | 757.08921 | 757.09044 | 1.6 | C20H27N10O16P3 |
| 283 | Okanin 4'-O-(2''-O-caffeoyl-6''-O-p-coumaroylglucoside) | [M+H]+ | 759.19196 | 759.19363 | 2.2 | C39H34O16 |
| 284 | Kaempferol 7-methyl ether 3-[3-hydroxy-3-methylglutaryl-(1->6)]-  [apiosyl-(1->2)-galactoside] | [M+Na]+ | 761.18995 | 761.18953 | -0.6 | C33H38O19 |
| 285 | Cinchonain-1a-(4β->8)-catechin | [M+Na]+ | 763.16334 | 763.16219 | -1.5 | C39H32O15 |
| 286 | Kaempferol 3-(2''-p-coumaryl-rhamnoside)-7-rhamnoside | [M+K]+ | 763.16349 | 763.16219 | -1.7 | C36H36O16 |
| 287 | Quercetin 3-(2''-galoylrutinoside) | [M+H]+ | 763.17162 | 763.17178 | 0.2 | C34H34O20 |
| 288 | Amaranthin | [M+K]+ | 765.13874 | 765.13818 | -0.7 | C30H34N2O19 |
| 289 | PC(14:0/20:0) | [M+Na]+ | 784.58268 | 784.58053 | -2.7 | C42H84NO8P |
| 290 | Heme-thiolate(P-450) | [M+Na]+ | 786.21449 | 786.21552 | 1.3 | C40H43FeN4O6S |
| 291 | PI(O-16:0/15:1) | [M+Na]+ | 803.50449 | 803.50404 | -0.6 | C40H77O12P |
| 292 | PG(O-16:0/22:6) | [M+Na]+ | 803.51974 | 803.51951 | -0.3 | C44H77O9P |
| 293 | PG(13:0/22:0) | [M+K]+ | 803.51989 | 803.51951 | -0.5 | C41H81O10P |
| 294 | PC(18:3/P-18:1) | [M+K]+ | 804.53040 | 804.53068 | 0.3 | C44H80NO7P |
| 295 | Okadaic acid | [M+H]+ | 805.47327 | 805.47096 | -2.9 | C44H68O13 |
| 296 | N-Acetyl-leu-leu-leu-leu-leu-tyr-amide | [M+K]+ | 826.48392 | 826.48456 | 0.8 | C41H69N7O8 |
| 297 | Lactosylceramide (d18:1/12:0) | [M+Na]+ | 828.54436 | 828.54239 | -2.4 | C42H79NO13 |
| 298 | PS(17:0/22:2) | [M+H]+ | 830.59056 | 830.58821 | -2.8 | C45H84NO10P |
| 299 | β-Alanyl-CoA | [M+H]+ | 839.15960 | 839.15940 | -0.2 | C24H41N8O17P3S |
| 300 | Delphinidin 3-(2-xylosylgalactoside)-5-(6-acetylglucoside) | [M+K]+ | 840.17211 | 840.17343 | 1.6 | C34H41O22 |
| 301 | Delphinidin 3-(6''-O-4-malyl-glucoside)-5-(6'''-O-1-malyl-glucoside) | [M+K]+ | 898.14120 | 898.13951 | -1.9 | C35H39O25 |
| 302 | Pelargonidin 3-rutinoside-7-(6-(p-hydroxybenzoyl)glucoside) | [M+K]+ | 900.20849 | 900.20738 | -1.2 | C40H45O21 |
| 303 | Salviamalvin | [M+Na]+ | 908.23455 | 908.23635 | 2.0 | C42H45O21 |
| 304 | PE(22:0/24:1) | [M+Na]+ | 908.70788 | 908.70716 | -0.8 | C51H100NO8P |
| 305 | 6-Carboxyhexanoyl-CoA | [M+H]+ | 910.18548 | 910.18388 | -1.8 | C28H46N7O19P3S |
| 306 | (S)-Hydroxyoctanoyl-CoA | [M+H]+ | 910.22187 | 910.22347 | 1.8 | C29H50N7O18P3S |
| 307 | PE(22:0/24:0) | [M+Na]+ | 910.72353 | 910.72588 | 2.6 | C51H102NO8P |
| 308 | Kaempferol 3-(6'''-rhamnosyl-2'''-(6-malyl-glucosyl)-glucoside) | [M+K]+ | 911.18541 | 911.18791 | 2.7 | C37H44O24 |
| 309 | PC(18:0/24:0) | [M+K]+ | 912.68182 | 912.68111 | -0.8 | C50H100NO8P |
| 310 | p-Coumaroyl-CoA | [M+H]+ | 914.15926 | 914.16004 | 0.8 | C30H42N7O18P3S |
| 311 | TG(20:0/20:0/20:0) | [M+H]+ | 975.93142 | 975.93319 | 1.8 | C63H122O6 |
| 312 | Ins-1-P-Cer(t18:0/26:0) | [M+K]+ | 976.66147 | 976.66168 | 0.2 | C50H100NO12P |
| 313 | PI(20:2/22:4) | [M+K]+ | 977.55159 | 977.55320 | 1.6 | C51H87O13P |
| 314 | N-Acetyl-D-glucosaminyl-N-acetylmuramoyl-L-Ala-D-glutamyl-6-  carboxy-L-lysyl-D-alanine | [M+K]+ | 978.35521 | 978.35633 | 1.1 | C37H61N7O21 |
| 315 | PC(24:0/24:1) | [M+Na]+ | 978.78613 | 978.78328 | -2.9 | C56H110NO8P |
| 316 | Quercetin 3-(6''''-ferulylsophorotrioside) | [M+Na]+ | 987.23769 | 987.23978 | 2.1 | C43H48O25 |
| 317 | Isorhamnetin 3-rhamnosyl-(1->2)-gentiobiosyl-(1->6)-glucoside | [M+K]+ | 987.23784 | 987.23978 | 2.0 | C40H52O26 |
| 318 | Peonidin 3-ferulyldiglucoside-5-glucoside | [M+K]+ | 1002.24019 | 1002.23991 | -0.3 | C44H51O24 |
| 319 | α-Semegma mycolic acid | [M+H]+ | 1124.16578 | 1124.16612 | 0.3 | C77H150O3 |
| 320 | NeuAcα2-3Galβ-Cer(d18:1/24:0) | [M+Na]+ | 1125.77476 | 1125.77629 | 1.4 | C59H110N2O16 |

| 321 | Quercetin 3-(2''-sinapoylglucoside)-3'-(6''-sinapoylglucoside)-4'-  glucoside | [M+H]+ | 1201.32422 | 1201.32495 | 0.6 | C55H60O30 |
| --- | --- | --- | --- | --- | --- | --- |
| a Cer: Ceramide; GalCer: Galactosylceramide; GlcCer: Glucosylceramide; ; LacCer: Lactosylceramide; MG: Monoacylglycerol; DG: Diacylglycerol; TG: Triacylglycerol; MGDG: Monoacyldiacylglycerol; PA: Phosphatidic acid; PC: Phosphatidylcholine; PE: Phosphatidylethanolamine; PG(P): Glycerophospholipids; PI: Phosphatidylinositol; PS: Phosphatidylserine; SM: Sphingomyelin; CDP: Cytidine diphosphate; UDP: Uridine diphospate; SQMG: sulfoquinovosylmonoacylglycerols | | | | | | |
